# Dynamic Covalent Michael Acceptors to Penetrate Cells: Thiol‐Mediated Uptake with Tetrel‐Centered Exchange Cascades, Assisted by Halogen‐Bonding Switches

**DOI:** 10.1002/anie.202213433

**Published:** 2022-11-17

**Authors:** Inga Shybeka, John R. J. Maynard, Saidbakhrom Saidjalolov, Dimitri Moreau, Naomi Sakai, Stefan Matile

**Affiliations:** ^1^ School of Chemistry and Biochemistry National Centre of Competence in Research (NCCR) Chemical Biology University of Geneva Geneva Switzerland

**Keywords:** Cellular Uptake, Dynamic Covalent Chemistry, Exchange Cascades, Reversible Michael Acceptors, Thiol-Mediated Uptake

## Abstract

Chalcogen‐centered cascade exchange chemistry is increasingly understood to account for thiol‐mediated uptake, that is, the ability of reversibly thiol‐reactive agents to penetrate cells. Here, reversible Michael acceptors are shown to enable and inhibit thiol‐mediated uptake, including the cytosolic delivery of proteins. Dynamic cyano‐cinnamate dimers rival the best chalcogen‐centered inhibitors. Patterns generated in inhibition heatmaps reveal contributions from halogen‐bonding switches that occur independent from the thyroid transporter MCT8. The uniqueness of these patterns supports that the entry of tetrel‐centered exchangers into cells differs from chalcogen‐centered systems. These results expand the chemical space of thiol‐mediated uptake and support the existence of a universal exchange network to bring matter into cells, abiding to be decoded for drug delivery and drug discovery in the broadest sense.

## Graphical abstract

Introduced as the first explicitly tetrel‐centered cascade exchangers, Michael acceptors are shown to enable and inhibit thiol‐mediated uptake, generate distinct patterns in heatmaps to decode uptake circuitry, integrate novel halogen‐bonding switches, deliver proteins to the cytosol, and break, as dimers, records for uptake inhibition related to drug discovery.

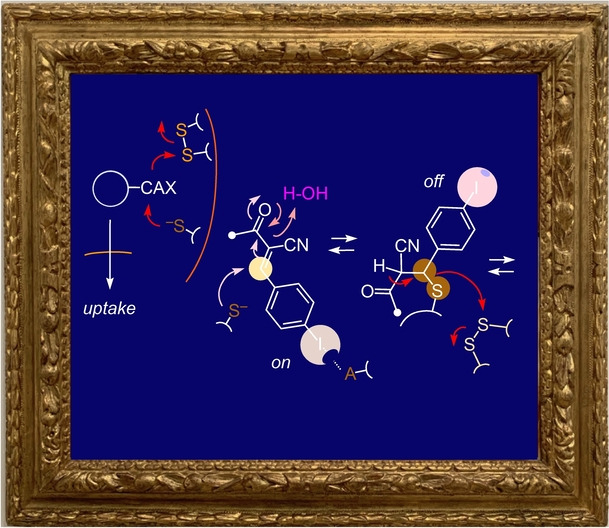

Thiol‐mediated uptake (TMU) refers to cell penetration of dynamic covalent cascade exchangers (CAXs) that can be inhibited by thiol‐reactive agents (Figure 1A).[^1^, ^2^] TMU excels in cytosolic delivery in deep tissue,^[3]^ and inhibitors are promising with regard to drug discovery, particularly as antivirals.[^4^, ^5^, ^6^, ^7^] So far, the focus has been almost exclusively on chalcogen‐centered CAXs, particularly cyclic or polymeric disulfides, e.g., **1** (Figure 1B).[^1^, ^6^] Recently, pnictogen‐centered CAXs have been introduced, including bismuth complex **2**, a powerful inhibitor of lentivirus entry.^[7]^ Here, we introduce tetrel‐centered CAXs to TMU.

**Figure 1:**
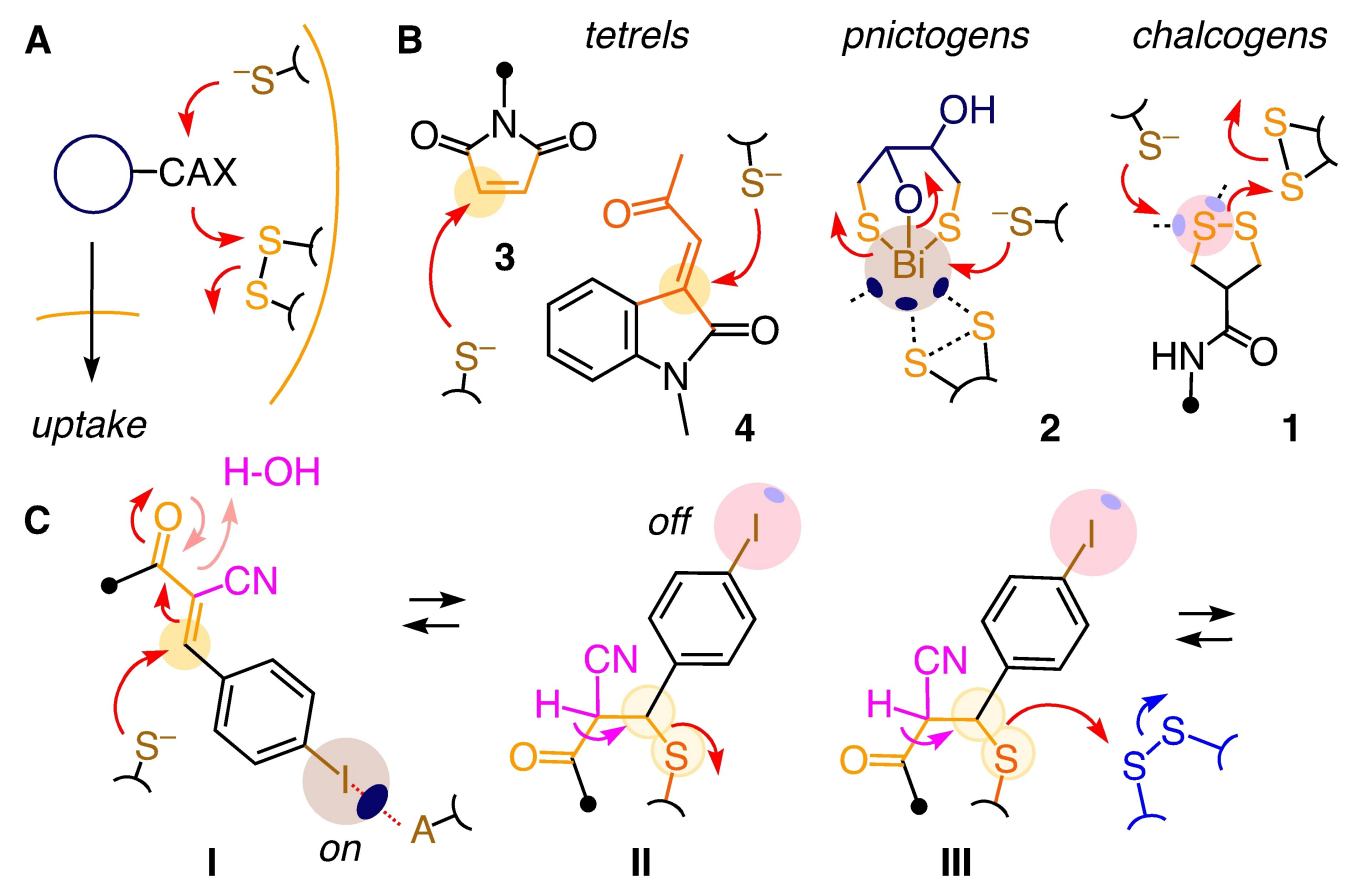
A) Graphical definition of TMU, CAX=cascade exchanger. B) Examples for CAXs with chalcogen (**1**) and pnictogen relays (**2**), and irreversible Michael acceptors **3** and **4** examined previously to enable or inhibit TMU. C) Reversible Michael acceptors **I** with halogen‐bonding switches (**I**–**II**) but only intermolecular access to disulfide exchange (**III**, •=rest of molecule).

Michael acceptors (MACs) were considered as possible CAXs because their irreversible bioconjugation to thiols is well known from covalent drugs.^[8]^ Maleimides **3** have been tested early on for TMU,^[9]^ with little follow‐up.^[10]^ Michael addition to maleimides is not dynamic.^[11]^ Half‐lives of addition products are around two weeks and not comparable with disulfide exchange cascades that occur within minutes.^[11]^ Thiol addition to the super‐cinnamaldehyde^[12]^ **4**, a potent activator of the pain receptor TRPA‐1^[12]^ and confirmed inhibitor of TMU,^[13]^ is also irreversible (Figure 1B).^[12]^ More irreversible MACs exist for chemoselective tagging of cysteine thiols.[^11^, ^14^, ^15^]

Recently, interest in dynamic‐covalent MACs intensified (Figure 1C).[^16^, ^17^, ^18^, ^19^, ^20^, ^21^] In α cyano‐cinnamates such as **I**, the cyano group increases electrophilicity and acidity at the α position of the cyano‐acetate motif in product **II**, thus enabling rapid equilibration (<30 min).^[16]^ Electron‐withdrawing *para* substituents increase the electrophilicity of **I** but not the acidity of the decoupled cyanoacetate in **II**, thus shifting the equilibrium to the side of product **II**, in direct correlation with the Hammett σ_p_[^15^, ^16^, ^17^, ^18^, ^22^] but without increasing residence time.^[23]^ For TMU, this meant that MACs like **I** could hop along cellular thiols (**I**, **II**), and disulfides could react with thiols released on‐site by retro‐Michael (**III**, Figure 1C). Their potential to enable TMU was further supported by their ability to reversibly attach to proteins,^[20]^ and enhance intracellular accumulation of PROTACs.^[21]^

MACs **5**–**14** were synthesized by adapting reported procedures (Figure 2, Scheme S1).^[16]^ MACs were stable in neutral water at room temperature for >10 h, confirming^[17]^ that hydration and retroaldol are negligible in the timeframe of cellular uptake (Figure S20). Fluorescent oligonucleotide phosphorothioates (OPS) **15** were selected as reporters of TMU^[24]^ into HeLa Kyoto (HK) cells (Figure 2C). Uptake inhibition is reported as decreasing cellular fluorescence with increasing concentration of, e.g., MAC **10** (Figure 2D). Automated high‐content high‐throughput imaging was used as described^[6]^ to simultaneously record dose response curves for inhibition and toxicity (Figure S9). Inhibitors were characterized by the concentration needed to inhibit TMU of OPS **15** by 15 % (MIC) and 50 % (IC_50_, Figure 2A, B, Table S1).

**Figure 2:**
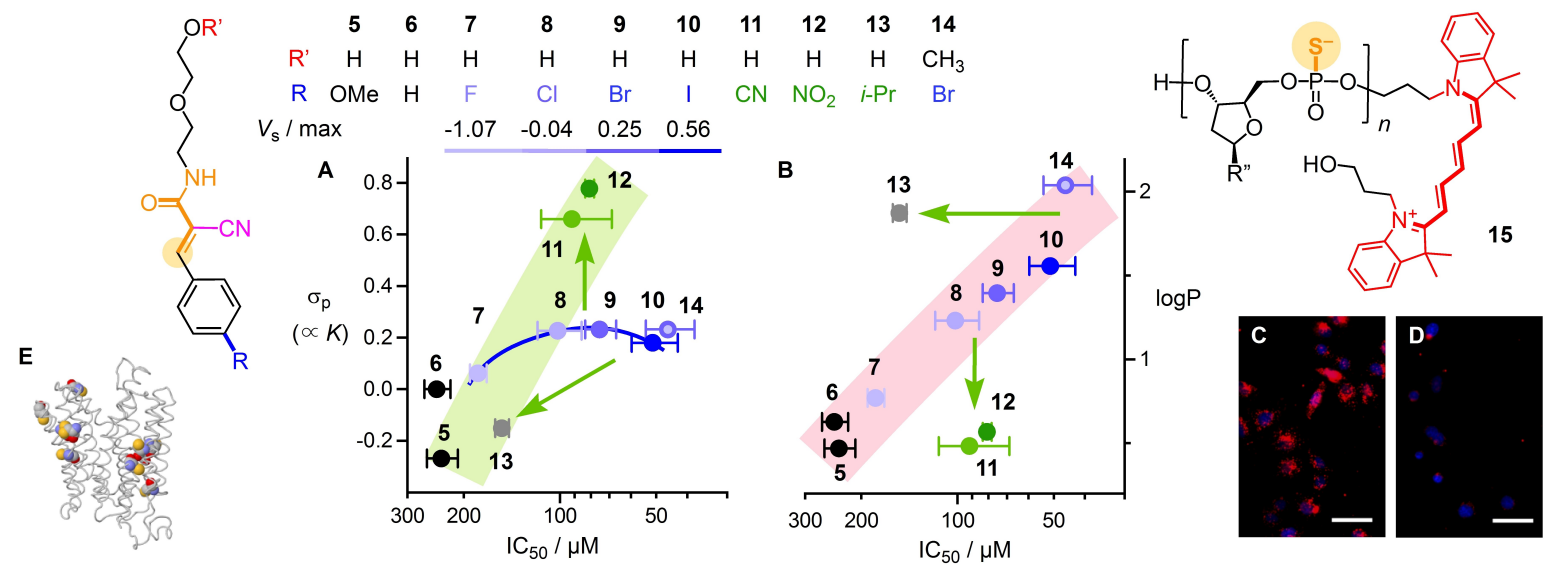
A, B) IC_50_ of Michael acceptors **5**–**14** to inhibit the uptake of OPS **15** into HK cells (500 nM; R′′=nucleobases, *n*=18: AGGTCCCCATACACCGAC), correlated to (A) σ_p_ of R and (B) logP (Chemicalize, Chemaxon). Data points are colored according to maxima on MEP surfaces of halogenated trifluoromethane (**7**–**10**, **14**; blue) or σ_p_ (**11**, **12**; green). C, D) Spinning disk confocal microscopy (SDCM) images of HK cells with **15** (red) without (C) and with (D) inhibitor **10** (200 μM; nuclei labeled with Hoechst 33342 (blue), scale bars 50 μm). (E) Predicted structure of MCT8 with Cys in CPK (C: grey, O: red, N: blue, S: yellow; SWISS‐MODEL P36021).

TMU inhibition increased with increasing Hammett σ_p_ of the *para* substituent of MACs **5**–**12** except for the halogenated **8**–**10** (Figure 2A, blue vs. green). The quasi‐linear dependence on the calculated logP of halogenated MACs **8**–**10** and methyl ether **14** was considered to explain their high activity (Figure 2B, red). However, the poor activity of **13** with high logP and low σ_p_ was inconsistent with this interpretation. Moreover, the high activity of the hydrophilic **11** and **12** with high σ_p_ confirmed that reactivity (green) rather than hydrophobicity (red) is decisive (Figure 2A, B, green arrows). Later on, the validity of this conclusion will be confirmed with MAC dimers that are exceptionally hydrophilic (logP=−2.0) and exceptionally active (IC_50_=6.0±2.0 μM, see below).

After ruling out hydrophobicity, halogen bonding^[25]^ was most likely to account for activities beyond expectations from σ_p_ (Figure 2A, green vs blue). This hypothesis was supported by increasing activity with the depth of the σ holes *V*_s_ of the trifluoromethyl counterparts of MACs **8**–**10** (Figure 2A, blue).^[25]^ Combining halogen bonds with tetrel‐centered exchange cascades was interesting because they must turn off upon addition to **I** and turn on again upon retro‐Michael of **II** (Figure 1C). Such halogen‐bonding switches could help immobilizing MAC **I** for addition of cellular thiols and then liberate the tethered MAC **II** to move on to the next site. Analogous chalcogen‐bonding switches have been essential to access twisted push‐pull probes to image membrane tension.^[26]^ Halogen bonding has been used previously to increase membrane association and permeability.[^27^, ^28^, ^29^] In cells, including HK cells, uptake inhibition by silychristin implied that MCT8, the thyroid hormone monocarboxylate transporter 8, is a target of halogen‐bond‐mediated uptake.^[28]^ MCT8 was conceivable for TMU because cysteine thiols line the transmembrane helix bundle (Figure 2E).^[30]^ However, silychristin and thyroxine, with IC_50_<10 μM for MCT8, failed to inhibit TMU of OPS **15** (Figure S11). Other halogen‐bonding proteins might thus be involved in TMU, several candidates exist in the literature.^[29]^

To elaborate on cytosolic delivery with tetrel‐centered CAXs, MAC **10** was selected because maximal contributions from halogen‐bonding cascade switches were considered the most promising to access unique properties. The fluorescently‐labeled version of MAC **10**, that is conjugate **16**, readily penetrated HK cells and accumulated mostly in cytosol and nucleus (Figures 3A, B, S12). The finestructure visible within the cytosol was characteristic of the endoplasmic reticulum (ER).^[31]^ This was interesting because ER trackers operate with irreversible thiol‐reactive agents, but dynamic covalent ER targeting has not been observed for other CAXs. Delivery to ER and nucleus but neither plasma membrane nor endolysosomes supported that MACs enter the cytosol by direct penetration and not by endocytosis. The fluorescence signal of cells treated with MAC **16** was more intense than with the original **17** ^[32]^ and control **18**, similar to thiosulfonate (CTO) **20**,^[6]^ and weaker than epidithiodiketopiperazine (ETP) **21** ^[33]^ and benzopolysulfane (BPS) **19** ^[34]^ (Figure 3E, G, red). These trends should, however, not be overinterpreted because signal intensity within cells depends strongly on CAXs (quenching), concentration, incubation time, cell culture, pH, and so on.

**Figure 3:**
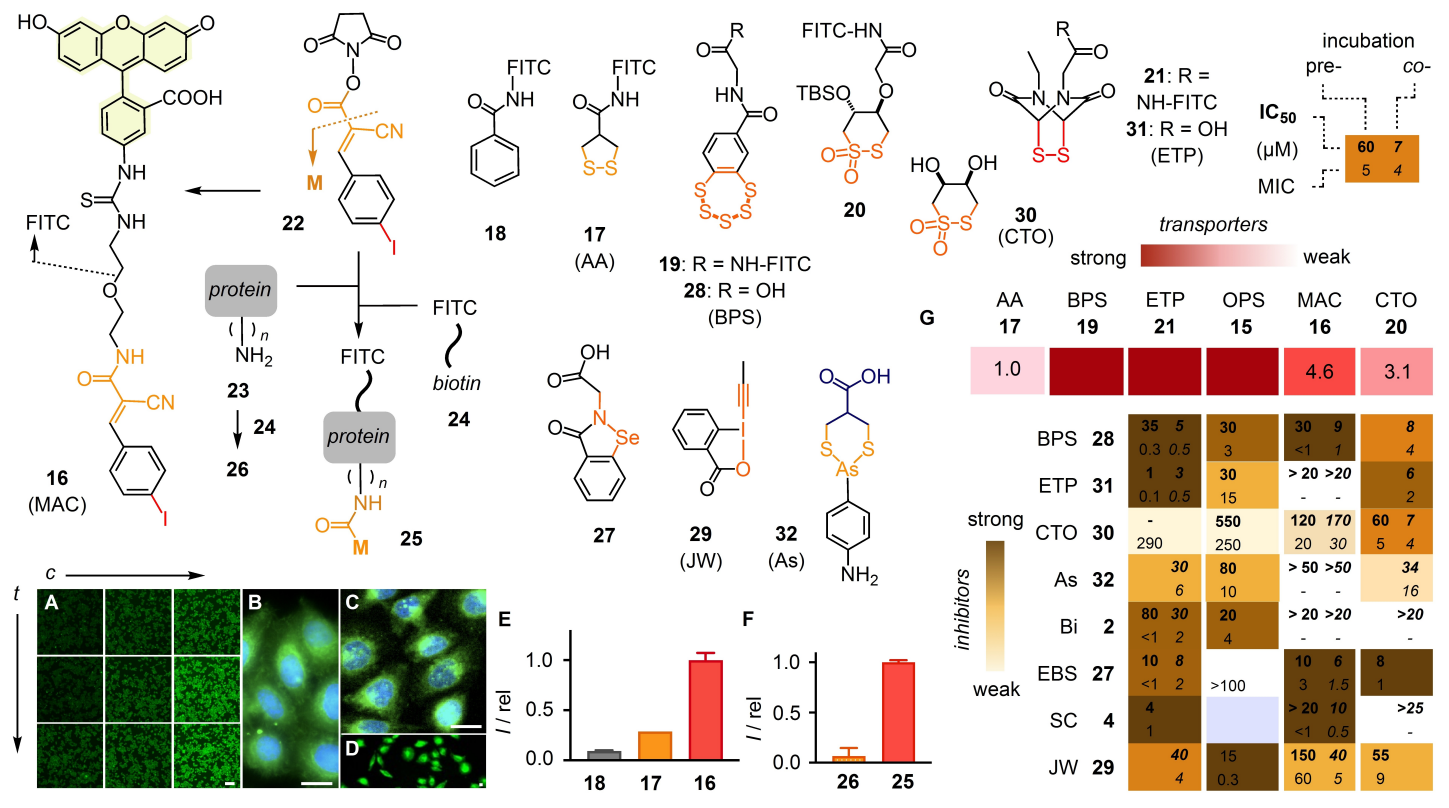
A–D) SDCM images of HK cells incubated with (A, B) **16** (A: 10, 20, 50 μM (left‐right) for 0.5, 1.0, 1.5 h (top‐down), scale bar: 200 μm; B: 50 μM, 1 h, scale bar: 20 μm) and (C, D) **25** (10 μM, 8 h, scale bars: 20 μm); blue: Hoechst 33342. E) Relative fluorescence intensity of HK cells incubated with **16** compared to **17** and **18** under identical conditions (50 μM, 1 h). F) Same with **25** compared to **26**. G) TMU heatmap with patterns from CAXs as transporters for cytosolic delivery (top, red) and as inhibitors of TMU (bottom, brown), showing uptake of fluorescent CAXs relative to **17** (top, red) and IC_50_ (top) and MIC (bottom) for the inhibition of TMU, determined under pre‐incubation (with, left) and co‐incubation conditions (without removal of inhibitors before transporter addition, right), in μM, including data from [5], [7], [13] (**21**), [6] (**20**) and [24] (**15**). ‐, not detectable; empty, not determined.

Protein delivery was explored by in situ conjugation of NHS‐esters **22** with lysines of streptavidin **23** (Figure 3). MALDI‐MS indicated the presence of up to eight MACs per streptavidin tetramer (Figure S18). Complexed with biotinylated FITC **24** for imaging, the MACylated protein **25** penetrated HK cells efficiently, while the fluorescent WT control **26** did not (Figure 3C, D, F).

Inhibition with thiol‐reactive agents is the hallmark of TMU.^[1]^ Uptake of MAC **16** was inhibited by several of the most established TMU inhibitors (Figure 3G). MIC and IC_50_ were determined using the pre‐incubation procedure, in which cells are treated with inhibitors, washed, and incubated with a transporter, or the co‐incubation procedure without the washing step (Figures S15, S16, Table S4). While quantitative MIC and IC_50_ depend on many parameters and should not be overinterpreted, color‐coded trends determined in parallel characterize transporters more reliably. In the resulting heatmap, the pattern generated by CAX **16** did not match patterns of chalcogen‐centered CAXs **15**, **20** and **21** (Figure 3G). Best was ebselen **27**, effective also against CTO **20** and the entry of SARS‐CoV‐2 lentivectors,^[6]^ but not against OPS **15**.^[24]^ Inhibition by SC **4** was meaningful because this is an excellent but irreversible Michael acceptor.^[12]^ The natural product inspired BPS is generally most active as transporter (**19**) and inhibitor (**28**).^[34]^ Less active were the irreversible **29**[^13^, ^35^] and rapidly exchanging inhibitors like **30**–**32** and **2**. This was particularly remarkable for ETP **31** because this is otherwise an excellent inhibitor.[^5^, ^6^]

The distinct pattern generated by inhibitors was important, first of all, to support that MAC **16** indeed enters cells by TMU and not by passive diffusion. Distinct selectivity patterns were understandable considering that to exchange with disulfides, MAC **16** requires an extra thiol (**III**, Figure 1C) and involves turn‐on halogen bonding (Figures 1C, 2A). It thus strengthened the emerging view of TMU as a dynamic covalent network with diverse exchange partners and pathways to enter into cells.^[1]^ To further elaborate on cascade exchange, MAC dimer **33** was designed and synthesized (Figure 4). With two tetrel‐centered relays, **33** is a true CAX, i.e., offers another exchanger upon dynamic covalent exchange. Although less likely according to precedence[^5^, ^32^] and preliminary results, the disulfide bridge in **33** could also exchange with thiols to liberate monomer **IV** with a tethered thiol for cellular disulfides or to cyclize into a “conjugate thiolactone” **V** as a caged reactivity homolog of cyclic disulfides.^[19]^

**Figure 4:**
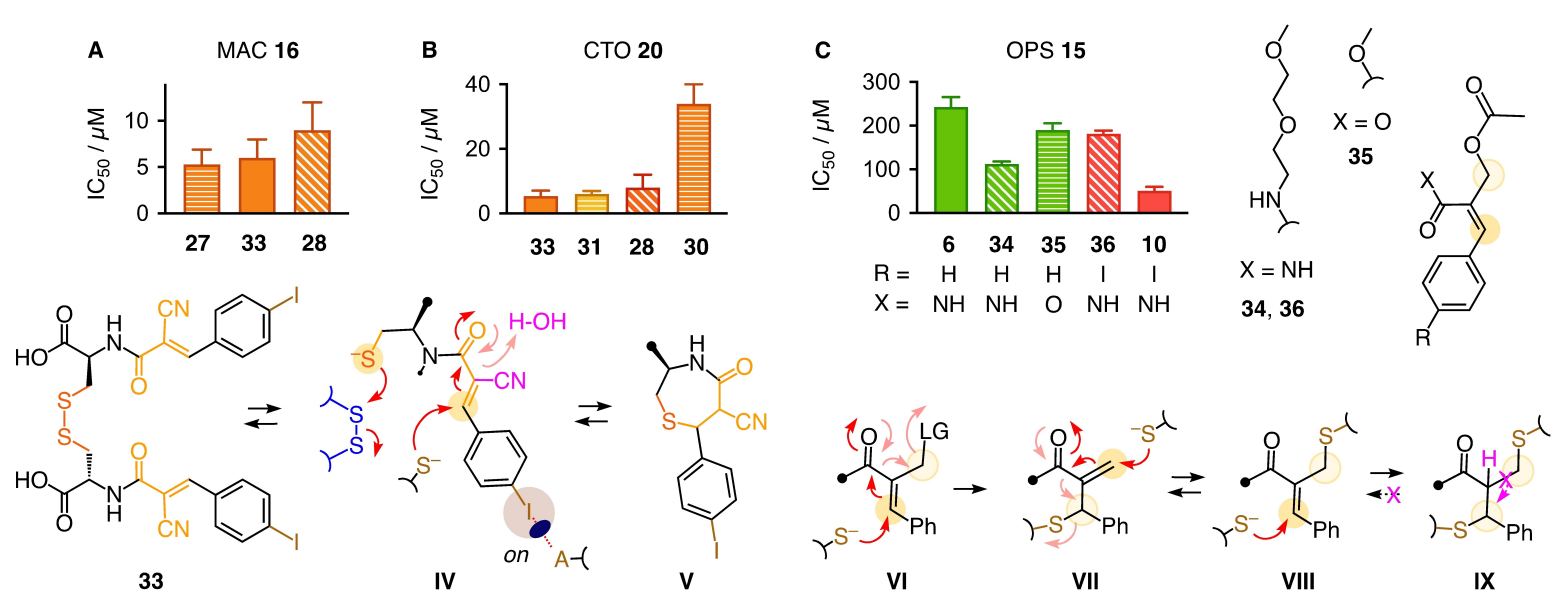
IC_50_ to inhibit TMU of A) MAC **16** by MAC **33** compared to **27** and **28**, B) CTO **20** by **33** compared to **31**, **30** and **28**, and C) OPS **15** by **34**–**36** compared to **6** and **10**, from automated high‐content high‐throughput imaging of HK cells, with possible exchange cascade intermediates **IV**–**IX**.

Dimer **33** was an outstanding inhibitor. With an IC_50_=6.0±2.0 μM, the formal “self‐inhibition” of MAC transporter **16** was almost as good as the current best, ebselen **27** and slightly better than the powerful BPS **28** (Figures 4A, 3G, S16, Table S4). For the CTO transporter **20**, MAC dimer **33** was with IC_50_=5.0±2.0 μM the absolute best, slightly better than classics such as ETP **31** or BPS **28**, as well as self‐inhibition with CTO **30** (Figures 4B, S17, Table S5). The exceptional activity of double MAC **33** compared to monomer **10** was consistent with definition and power of CAXs and thus cascade exchange accounting for TMU. The high hydrophilicity of **33** (logP=−2.0 at pH∼7) validated the conclusion from above monomer screening (Figure 2) that reactivity and not hydrophobicity accounts for activity.

An alternative approach toward tetrel‐centered CAXs was considered with dual‐reactive MACs **34**–**36** (Figure 4C). Their allylic leaving group in **VI** produces a new MAC **VII** upon Michael addition.[^36^, ^37^, ^38^, ^39^, ^40^] From intermediate **VII**, exchange cascades can unfold only if retro‐Michael thiolate release to acceptor **VIII** exceeds protonation. Irreversible double‐addition products **IX** have been used to target vicinal thiols in cells.^[36]^ To allow retro‐Michael from **IX**, the acidity of the α proton would have to increase. This strategy has been explored for equilibrium transfer alkylation of proteins,^[37]^ to build molecular walkers,^[38]^ and to label proteins site‐specifically while releasing the ligand.^[39]^

The addition of an allylic ester to cinnamate original **6** gave better inhibitors (Figure 4C, S10, Table S2). The best was amide **34**, which at similar logP shifted the IC_50_ from 240 μM of **6** to 110 μM, while ester **35** was less convincing despite more positive logP. Whereas halogen‐bond switches turned dynamic Michael acceptors **10** into efficient inhibitors of TMU (Figure 2), they weakened dynamic dual acceptors **36** (Figure 4C). Overall, dual MACs **34**–**36** were clearly less promising than MAC dimers **33** for future developments. These perspectives are attractive considering the large chemical space opened up by this study: Tetrel‐centered cascade exchangers are introduced to thiol‐mediated uptake, with focus on dynamic covalent Michael acceptors in various forms for transport, inhibition and as mechanistic tools to elucidate the complex networks at work to bring matter into cells.

## Conflict of interest

The authors declare no conflict of interest.

## Supporting information

As a service to our authors and readers, this journal provides supporting information supplied by the authors. Such materials are peer reviewed and may be re‐organized for online delivery, but are not copy‐edited or typeset. Technical support issues arising from supporting information (other than missing files) should be addressed to the authors.

Supporting Information

## Data Availability

The data that support the findings of this study are openly available in zenodo at https://doi.org/10.5281/zenodo.7164389, reference number 7164389.
