# Supplementary material for: Dynamic Covalent Michael Acceptors to Penetrate Cells: Thiol‐Mediated Uptake with Tetrel‐Centered Exchange Cascades, Assisted by Halogen‐Bonding Switches
Source: Angew Chem Int Ed Engl. 2022 Nov 17;61(51):e202213433. doi: 10.1002/anie.202213433 (PMC10098706; doi:10.1002/anie.202213433)
Supplement: Supplementary file 1 — Supporting Information [file ANIE-61-0-s001.pdf]

Angewandte Chemie
Eine Zeitschrift der Gesellschaft Deutscher Chemiker

Supporting Information

Dynamic Covalent Michael Acceptors to Penetrate Cells: Thiol-Mediated Uptake with Tetrel-Centered Exchange Cascades, Assisted by Halogen-Bonding Switches

*I. Shybeka, J. R. J. Maynard, S. Saidjalolov, D. Moreau, N. Sakai, S. Matile**

Table of Content

1. Materials and Methods S3
2. Synthesis S5
    2.1. Synthesis of MAC Inhibitors S5
    2.2. Synthesis of MAC Reporters S14
3. Cell Culture S18
4. Automated High-Content High-Throughput (AHCHT) Screening S18
    4.1. General Procedure for AHCHT Inhibitor Screening with OPS Reporters S18
    4.2. Data Analysis for AHCHT Inhibitor Screening with OPS Reporters S19
    4.3. Results for AHCHT Inhibitor Screening with OPS Reporters S25
    4.4. Cellular Uptake of MAC Reporters S30
    4.5. General Procedure for AHCHT Inhibitor Screening with MAC Reporters and Controls S32
    4.6. Data Analysis for Uptake and Inhibition of MAC Reporters and Controls S34
    4.7. Results for AHCHT Inhibitor Screening with MAC Reporters S35
    4.8. Results for AHCHT Inhibitor Screening with CTO Reporters S38
5. Protein Uptake S39
    5.1. Preparation of Streptavidin Complexes S39
    5.2. Cellular Uptake of Streptavidin Complexes S42
6. Stability of Michael Acceptors in Aqueous Media S44
7. Supporting References S45
8. NMR Spectra S47

1. Materials and Methods

As in ref. [S1]. Briefly, reagents for synthesis and commercially available final compounds were purchased from Sigma-Aldrich, Brunschwig, Alfa Aesar, Merck, TCI, Acros, Iris Biotech, and BroadPharm. Phosphate buffered saline (PBS, pH = 7.4), DMEM (GlutaMAX, 4.5 g/L D-glucose, with phenol red) medium, FluoroBrite DMEM (high D-Glucose) medium, Penicillin- Streptomycin, Fetal Bovine Serum, TrypLE Express Enzyme and V96-MicroWell platen were obtained from Thermo Fisher Scientific. μ-Plate 96-Well Black were obtained from Ibidi. Hoechst 33342 (HOE, 10 mg/mL solution in water) and propidium iodide (PI, 1.0 mg/mL solution in water) were obtained from Invitrogen by Thermo Fisher Scientific. OPS (Cy5-*A*G*G*T*C*C*C*C*A*T*A*C*A*C*C*G*A*C) were purchased from Merck KGaA and used as received. WTS was a generous gift from Prof. Thomas R. Ward (University of Basel).

UV-Vis spectra were recorded on a JASCO V-650 spectrophotometer equipped with a stirrer and a temperature controller (20 ºC) and are reported as maximal absorption wavelength λ in nm (extinction coefficient ε in M^{-1} cm^{-1}).

Column chromatography was carried out on silica gel (SiliaFlash® P60, SILICYCLE, 230 – 400 mesh). Analytical thin layer chromatography (TLC) was performed on silica gel 60 F_{254} (Merck). Reverse phase flash chromatography was performed on Biotage Isolera™ Four (eluents: CH_3CN and H_2O with 0.1% TFA). pH values were measured with a Consort C832 multi- parameter analyzer equipped with a VWR glass membrane pH electrode calibrated with Titrisol solution from Merck at pH 4.00, 7.00 and 10.01. Melting points (Mp) were measured on a Melting Point M-565 (BUCHI). IR spectra were recorded on a Perkin Elmer Spectrum 100 FT-IR spectrometer (ATR, Golden Gate, unless stated) and are reported as wavenumbers ν in cm^{-1} with band intensities indicated as br (broad), s (strong), m (medium), w (weak). NMR spectra were recorded (as indicated) either on a Bruker 400 or 500 MHz spectrometer. ^1H NMR

spectra are reported as chemical shifts (δ) in ppm relative to the internal $CHCl_3$ signal at 7.26 ppm, the internal CHD_2CN signal at 1.94 ppm or the internal $D_3CSOCHD_2$ signal at 2.50 ppm as the standard. Spin multiplicities are reported as singlet (s), doublet (d), triplet (t) and quartet (q) with coupling constants (*J*) given in Hz, or multiplet (m). Broad peaks are marked as br. ^{13}C NMR spectra are reported as chemical shifts (δ) in ppm relative to the internal $CDCl_3$ signal at 77.16 ppm, the internal CD_3CN signal at 1.32 ppm or the internal D_3CSOCD_3 signal at 39.52 ppm as the standard. ^1H and ^{13}C resonances were assigned with the aid of additional information from 1D and 2D NMR spectra (H,H-COSY, DEPT 135, HSQC and HMBC). MALDI MS analyses for the characterization of new compounds were performed with sinapic acid as a matrix using Bruker MALDI Autoflex Speed TOF/TOF. ESI-HRMS was measured on Xevo G2-S Tof (Waters). All mass data are reported as mass-per-charge ratio *m/z* (intensity in %, [assignment]). Fluorescence cellular imaging was performed using an IXM-C automated microscope from ImageXpress equipped with a Lumencor Aura III with 5 independently selectable solid-state light sources, bandpass filters and 5 objectives (4x to 60x). Sample preparation and washing on µ-Plate 96-Well Black was performed using a Plate washer Biotek EL406®.

Abbreviations. AMPSO: (*N*-(1,1-Dimethyl-2-hydroxyethyl)-3-amino-2-hydroxypropane sulphonic Acid); aq.: Aqueous; Boc: *tert*-Butoxycarbonyl; CLSM: Confocal laser scanning microscopy; DABCO: 1,4-Diazabicyclo[2.2.2]octane; DIPEA: *N,N*-Diisopropylethylamine; DMAP: 4-(Dimethylamino)pyridine; DMEM: Dulbecco's modified eagle medium; EDCI: 1-Ethyl-3-(3- dimethylamino-propyl)carbodiimide; ESI: Electrospray ionization; FBS: Fetal bovine serum; FITC: Fluorescein isothiocyanate; HATU: 1-[Bis(dimethylamino)methylene]-1H-1,2,3- triazolo[4,5-b]pyridinium 3-oxide hexafluoro-phosphate); AHCHT: Automated high-content high-throughput; HOE: Hoechst 33342; IC_{50}: Half maximal inhibitory concentration; MALDI: Matrix-assisted laser desorption/ionization;

MBH: Morita–Baylis–Hillman adduct; MICs: Minimum inhibitory concentrations; Mp: Melting point; NHS: *N*-Hydroxysuccinimide; OPS: Oligonucleotide phosphorothioate; PBS: Phosphate buffered saline; PI: Propidium iodide; quant.: Quantitative yield; R_f: Retention factor; rt: Room temperature; RV: Relative viability; sat.: Saturated; SDCM: Spinning disk confocal microscopy; SEM: Standard error of mean; TBS: *tert*-Butyldimethylsilyl; TFA: Trifluoroacetic acid; WTS: Wild-type streptavidin.

2. Synthesis

2.1. Synthesis of MAC Inhibitors

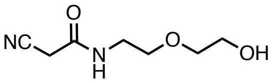

37

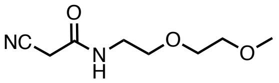

38

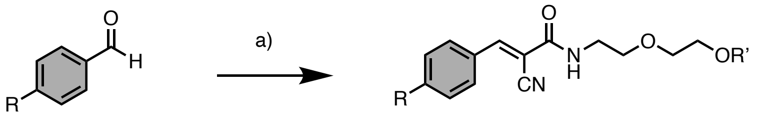

39: R = F
40: R = Br
41: R = I
42: R = CN
43: R = *i*-Pr

5: R = OMe; R' = H
6: R = H; R' = H
7: R = F; R' = H
8: R = Cl; R' = H
9: R = Br; R' = H
10: R = I; R' = H
11: R = CN; R' = H
12: R = NO_2; R' = H
13: R = *i*-Pr; R' = H
14: R = Br; R' = CH_3

Scheme S1. (a) **37**/**38**, piperidine, EtOH, 60 °C, 7-18 h, 13% (**7**), 47% (**9**), 44% (**10**), 19% (**11**), 79% (**13**), 46% (**14**).

Compounds 5, 6, 8, 12, 37, 38 were synthesized and purified according to procedures described in reference [S2].

Compound 7. The benzaldehyde **39** (100 mg, 0.81 mmol) and compound **37** (152 mg, 0.88 mmol) were mixed together with piperidine (100 µL) in EtOH (28 mL) and the mixture was stirred at 60 °C for 16 h. After the solvent was evaporated, the crude product was purified by flash column chromatography (Scorpius Silica 12 g, 35 mL/min, gradient 0 – 10% MeOH

in CH_2Cl_2) and PTLC (CH_2Cl_2/MeOH 95:5). The resulting solid was triturated with Et_2O to afford product **7** (32 mg, 13%) as a yellow solid. R_f (CH_2Cl_2/MeOH 95:5): 0.38; Mp: 76-77 °C; IR (neat): 3352 (w), 2928 (w), 2871 (w), 2215 (w), 1740 (m), 1668 (s), 1599 (s), 1510 (s), 1461 (w), 1355 (w), 1281 (w), 1234 (s), 1204 (m), 1161 (m), 1126 (m), 1067 (w), 841 (m); ^{1}H NMR (400 MHz, $CDCl_3$): 8.29 (s, 1H), 7.97 (dd, $^3J_{H\text{-}H}$ = 8.7 Hz, $^4J_{H\text{-}F}$ = 5.3 Hz, 2H), 7.19 (dd, $^3J_{H\text{-}H}$ = 8.7 Hz, $^3J_{H\text{-}F}$ = 8.7 Hz, 2H), 6.87 (br s, 1H), 3.79 (t, $^3J_{H\text{-}H}$ = 4.5 Hz, 2H), 3.75 – 3.54 (m, 6H), 2.06 (s, 1H); ^{13}C NMR (101 MHz, $CDCl_3$): 166.3 (d, $^1J_{C\text{-}F}$ = 256.8 Hz, CH), 160.4 (C), 151.7 (CH), 133.3 (d, $^3J_{C\text{-}F}$ = 9.1 Hz, CH), 128.3 (d, $^4J_{C\text{-}F}$ = 3.2 Hz, C), 117.1 (C), 116.8 (d, $^2J_{C\text{-}F}$ = 22.2 Hz, CH), 103.7 (C), 72.4 (CH_2), 69.5 (CH_2), 62.1 (CH_2), 40.5 (CH_2); HRMS (ESI, +ve) calcd for $C_{14}H_{15}FN_2O_3$ $[M+Na]^+$: 301.0959, found: 301.0950.

Compound 9. The benzaldehyde **40** (215 mg, 1.16 mmol) and compound **37** (200 mg, 1.16 mmol) were mixed together with piperidine (100 μL) in EtOH (25 mL) and the mixture was stirred at 60 °C for 15 h. After the solvent was evaporated, the crude product was purified by flash chromatography (Scorpius Silica 12 g, 35 mL/min, gradient 0 – 8% MeOH in CH_2Cl_2) to yield compound **9** (185 mg, 47%) as a yellow solid. R_f (CH_2Cl_2/MeOH 95:5): 0.38; Mp: 97-98 °C; IR (neat): 3370 (m), 2932 (w), 2879 (w), 2213 (w), 1676 (s), 1605 (m), 1583 (w), 1524 (s), 1487 (m), 1404 (w), 1351 (w), 1263 (m), 1206 (w), 1116 (s), 1080 (m), 1071 (w), 1048 (s), 1008 (w), 894 (w), 866 (w), 821 (m), 786 (w); ^{1}H NMR (400 MHz, $CDCl_3$): 8.21 (s, 1H), 7.74 (d, $^3J_{H\text{-}H}$ = 8.5 Hz, 2H), 7.58 (d, $^3J_{H\text{-}H}$ = 8.5 Hz, 2H), 7.06 (s, 1H), 3.78 – 3.71 (m, 2H), 3.70 – 3.58 (m, 6H), 2.63 (s, 1H); ^{13}C NMR (101 MHz, $CDCl_3$): 160.3 (C), 151.5 (CH), 132.6 (CH), 131.8 (CH), 130.6 (C), 127.5 (C), 116.7 (C), 104.8 (C), 72.3 (CH_2), 69.3 (CH_2), 61.7 (CH_2), 40.4 (CH_2); HRMS (ESI, +ve) calcd for $C_{14}H_{15}BrN_2O_3$ $[M+Na]^+$: 361.0159, found: 361.0158.

Compound 10. The benzaldehyde **41** (100 mg, 0.43 mmol) and compound **37** (81 mg, 0.47 mmol) were mixed together with piperidine (100 μL) in EtOH (15 mL) and the mixture was stirred at 60 °C for 16 h. After the solvent was evaporated, the crude product was purified

by flash chromatography (Scorpius Silica 12 g, 35 mL/min, gradient 0 – 10% MeOH in CH_2Cl_2). The resulting solid was washed with Et_2O to afford product **10** as a yellowish solid (80 mg, 44%). R_f (CH_2Cl_2/MeOH 95:5): 0.37; Mp: 105-106 °C; IR (neat): 3348 (m), 2926 (w), 2869 (w), 2213 (w), 1667 (s), 1601 (m), 1581 (m), 1525 (s), 1483 (m), 1399 (w), 1351 (w), 1277 (m), 1208 (w), 1190 (w), 1124 (m), 1062 (m), 1004 (w), 886 (w), 817 (w); ^{1}H NMR (400 MHz, $CDCl_3$): 8.24 (s, 1H), 7.85 (d, $^3J_{H-H}$ = 8.3 Hz, 2H), 7.64 (d, $^3J_{H-H}$ = 8.3 Hz, 1H), 6.87 (s, 1H), 3.85 – 3.76 (m, 2H), 3.70 – 3.60 (m, 6H), 2.05 (s, 1H); ^{13}C NMR (101 MHz, $CDCl_3$): 160.3 (C), 151.9 (C), 138.8 (CH), 131.8 (CH), 131.3 (C), 116.9 (C), 104.9 (C), 100.3 (C), 72.4 (CH_2), 69.5 (CH_2), 62.0 (CH_2), 40.5 (CH_2); HRMS (ESI, +ve) calcd for $C_{14}H_{15}N_2O_3I$ $[M+Na]^+$: 409.0020, found: 409.0029.

Compound 11. The benzaldehyde **42** (76 mg, 0.58 mmol) and compound **37** (100 mg, 0.58 mmol) were mixed together with piperidine (75 μL) in EtOH (10 mL) and the mixture was stirred at 60 °C for 18 h. After the solvent was evaporated, the crude product was purified by column chromatography (Scorpius Silica 12 g, 35 mL/min, gradient 0 – 4% MeOH in CH_2Cl_2) to yield compound **11** (31 mg, 19%) as a yellowish solid. R_f (CH_2Cl_2/MeOH 95:5): 0.50; Mp: 120-121 °C; IR (neat): 3469 (m), 3282 (w), 2954 (w), 2889 (w), 2873 (w), 2230 (w), 2213 (w), 1672 (s), 1599 (m), 1528 (s), 1491 (m), 1434 (w), 1278 (m), 1208 (w), 1136 (s), 1058 (m), 1025 (w), 987 (w), 959 (w), 838 (w), 680 (w); ^{1}H NMR (400 MHz, $CDCl_3$): 8.32 (s, 1H), 7.99 (d, $^3J_{H-H}$ = 8.3 Hz, 2H), 7.78 (d, $^3J_{H-H}$ = 8.3 Hz, 2H), 7.00 (s, 1H), 3.82 – 3.76 (m, 2H), 3.72 – 3.56 (m, 6H), 2.11 (s, 1H); ^{13}C NMR (101 MHz, $CDCl_3$): 159.5 (C), 150.4 (CH), 135.8 (C), 132.9 (CH), 130.7 (CH), 117.9 (C), 116.2 (C), 115.6 (C), 107.9 (C), 72.3 (CH_2), 69.3 (CH_2), 62.0 (CH_2), 40.6 (CH_2); HRMS (ESI, +ve) calcd for $C_{15}H_{15}N_3O_3$ $[M+Na]^+$: 308.1006, found: 308.1003.

Compound 13. The benzaldehyde **43** (200 mg, 1.35 mmol) and compound **37** (254 mg, 1.48 mmol) were mixed together with piperidine (100 μL) in EtOH (30 mL) and the mixture

was stirred at 60 °C for 7 h. After the solvent was evaporated, the crude product was purified by flash chromatography (Scorpius Silica 12 g, 35 mL/min, gradient 0 – 10% MeOH in CH_2Cl_2) and to afford product **13** as a colorless oil (354 mg, 79%). R_f (CH_2Cl_2/MeOH 95:5): 0.30; IR (neat): 3362 (w), 2961 (w), 2934 (w), 2213 (w), 1664 (s), 1595 (s), 1523 (s), 1463 (m), 1422 (m), 1363 (w), 1279 (m), 1212 (m), 1189 (m), 1124 (s), 1057 (s), 910 (w), 888 (w), 883 (m), 732 (m); ^{1}H NMR (400 MHz, $CDCl_3$): 8.30 (s, 1H), 7.87 (d, $^3J_{H-H}$ = 8.4 Hz, 2H), 7.35 (d, $^3J_{H-H}$ = 8.4 Hz, 2H), 6.86 (s, 1H), 3.80 (br s, 2H), 3.71 – 3.63 (m, 6H), 2.97 (h, $^3J_{H-H}$ = 6.9 Hz, 1H), 2.15 (s, 1H), 1.27 (d, $^3J_{H-H}$ = 6.9 Hz, 6H); ^{13}C NMR (101 MHz, $CDCl_3$): 160.9 (C), 154.8 (C), 153.1 (CH), 131.1 (CH), 129.6 (C), 127.5 (CH), 117.4 (C), 102.8 (C), 72.4 (CH_2), 69.6 (CH_2), 62.0 (CH_2), 40.4 (CH_2), 34.5 (CH), 23.7 (CH_3); HRMS (ESI, +ve) calcd for $C_{17}H_{22}N_2O_3$ $[M+Na]^+$: 325.1523, found: 325.1538.

Compound 14. 4-Bromobenzaldehyde **40** (100 mg, 0.54 mmol) and compound **38** (101 mg, 0.54 mmol) were mixed together with piperidine (100 µL) in EtOH (20 mL) and the mixture was stirred at 60 °C for 16 h. After the solvent was evaporated, the crude product was purified by flash chromatography (Scorpius Silica 12 g, 35 mL/min, gradient 0 – 10% MeOH in CH_2Cl_2) to yield product **14** as a colorless oil (97 mg, 46%). R_f (CH_2Cl_2/MeOH 98:2): 0.20; IR (neat): 3416 (m), 2997 (w), 2911 (w), 2196 (br), 2093 (br), 1663 (w), 1436 (m), 1312 (m), 1020 (s), 952 (m), 933 (m), 701 (m); ^{1}H NMR (400 MHz, CD_3CN): 8.16 (s, 1H), 7.85 (d, $^3J_{H-H}$ = 8.5 Hz, 2H), 7.71 (d, $^3J_{H-H}$ = 8.5 Hz, 2H), 6.98 (s, 1H), 3.65 – 3.59 (m, 4H), 3.53 – 3.49 (m, 4H), 3.33 (s, 3H); ^{13}C NMR (101 MHz, CD_3CN): 161.3 (C), 151.1 (CH), 133.4 (CH), 132.8 (CH), 132.3 (C), 127.3 (C), 117.2 (C), 107.5 (C), 72.6 (CH_2), 70.8 (CH_2), 69.6 (CH_2), 59.0 (CH_2), 40.8 (CH_3); HRMS (ESI, +ve) calcd for $C_{15}H_{17}BrN_2O_3$ $[M+Na]^+$: 375.0315, found: 375.0302.

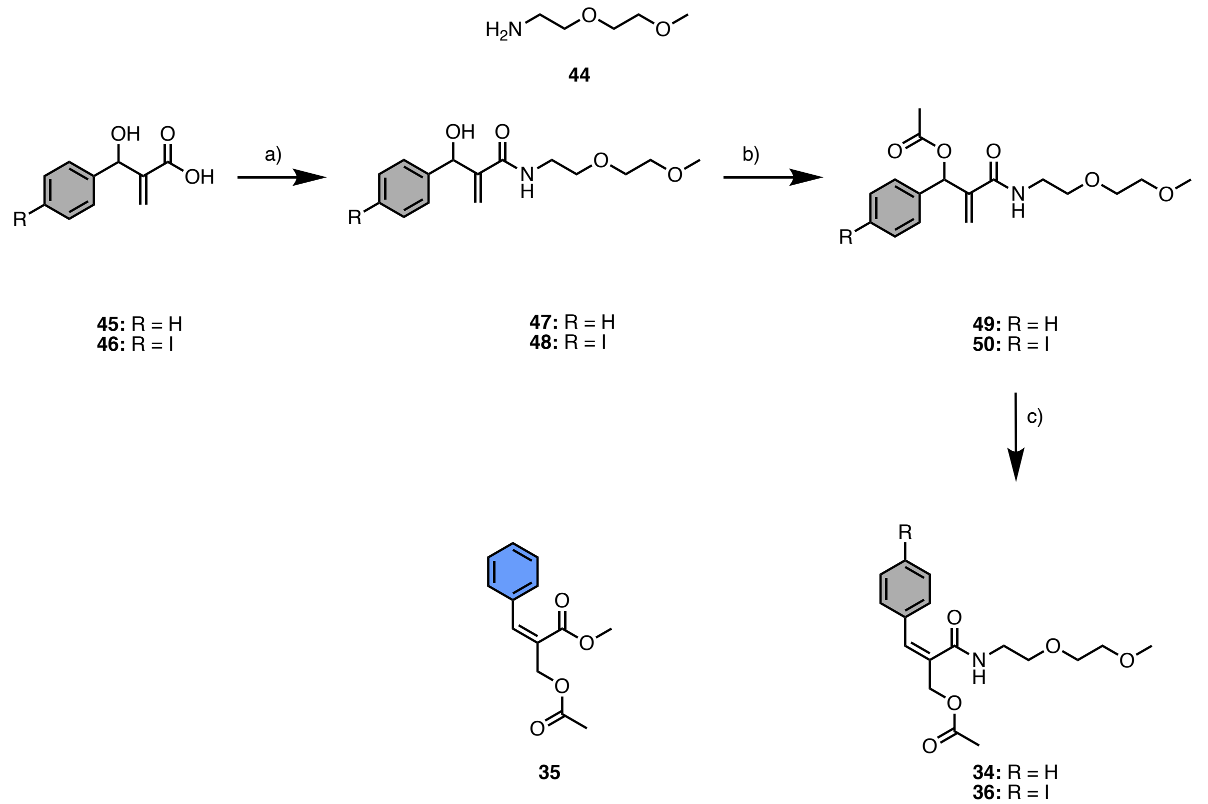

Scheme S2. (a) **44**, EDCI·HCl, DMAP, CH_2Cl_2, rt, 3-18 h, 89% (**47**), 48% (**48**); (b) Ac_2O, H_2SO_4, rt, 45 min; (c) DABCO, THF, 50 °C, 16 h – 48 h, 14% (**34**), 8% (**36**) (two steps).

Compound 44 was synthesized and purified according to procedures described in reference [S3].

Compounds 45, 46 were synthesized and purified according to procedures described in reference [S4].

Compound 47. To a solution of MBH adduct **45** (500 mg, 2.81 mmol) in CH_2Cl_2 (10 mL) were added EDCI·HCl (645 mg, 3.37 mmol) and DMAP (410 mg, 3.37 mmol) followed by a solution of amine **44** (401 mg, 3.37 mmol) in CH_2Cl_2 (2 mL). The reaction mixture was stirred at rt for 18 h. Organic phase was washed by citric acid 10% (x2), H_2O (x1), sat. solution of $NaHCO_3$ (x2), and brine (x1), dried over Na_2SO_4, filtered, and concentrated in *vacuo*. The crude product was purified by flash column chromatography (Scorpius Silica 12 g, 35 mL/min, linear gradient 0 – 7% MeOH in CH_2Cl_2) to yield **47** as a colorless oil (695 mg, 89%). R_f

(CH_2Cl_2/MeOH 95:5): 0.17; IR (neat): 3319 (m), 2920 (w), 2877 (m), 1738 (w), 1655 (m), 1612 (s), 1536 (s), 1495 (w), 1450 (m), 1353 (w), 1300 (w), 1247 (w), 1198 (w), 1100 (s), 1041 (m), 1026 (m), 948 (w), 842 (w), 762 (w), 701 (m); ^{1}H NMR (400 MHz, $CDCl_3$): 7.41 – 7.31 (m, 4H), 7.30 – 7.25 (m, 1H), 6.75 (s, 1H), 5.81 (s, 1H), 5.53 (d, $^3J_{H-H}$ = 5.3 Hz, 1H), 5.43 (d, $^4J_{H-H}$ = 0.7 Hz, 1H), 4.05 (d, $^3J_{H-H}$ = 5.3 Hz, 1H), 3.57 – 3.50 (m, 4H), 3.49 – 3.44 (m, 2H), 3.36 (s, 3H); ^{13}C NMR (101 MHz, $CDCl_3$): 168.2 (C), 145.7 (C), 141.3 (C), 128.5 (CH), 127.7 (CH), 126.4 (CH), 120.4 (CH_2), 75.0 (CH), 72.0 (CH_2), 70.3 (CH_2), 69.7 (CH_2), 59.1 (CH_3), 39.3 (CH_2).

Compound 48. To a solution of MBH adduct **46** (145 mg, 477 µmol) in CH_2Cl_2 (2 mL), EDCI·HCl (110 mg, 572 µmol) and DMAP (90 mg, 734 µmol) were added and amine **44** (90 mg, 755 µmol) in CH_2Cl_2 (1 mL) was added the last. The reaction mixture was stirred at rt for 3 h. Organic phase was washed with citric acid 10% (x2), H_2O (x1), saturated solution of $NaHCO_3$ (x2), brine (x1), dried over Na_2SO_4, filtered, and concentrated in *vacuo*. The crude product was purified by flash column chromatography (Scorpius Silica 12 g, 35 mL/min, linear gradient 0 – 10% MeOH in CH_2Cl_2) to yield **48** as a yellowish solid (93 mg, 48%). R_f (CH_2Cl_2/MeOH 95:5): 0.15; IR (neat): 3337 (m), 2881 (m), 1740 (w), 1654 (s), 1618 (s), 1579 (w), 1536 (s), 1485 (m), 1399 (w), 1355 (w), 1289 (m), 1236 (m), 1202 (w), 1104 (s), 1063 (m), 1025 (m), 1006 (m), 837 (w), 815 (w); ^{1}H NMR (400 MHz, $CDCl_3$): 7.68 (d, $^3J_{H-H}$ = 8.1 Hz, 2H), 7.15 (d, $^3J_{H-H}$ = 8.1 Hz, 2H), 6.70 (s, 1H), 5.78 (s, 1H), 5.45 (s, 1H), 5.44 (s, 1H), 3.58 – 3.55 (m, 2H), 3.54 – 3.50 (m, 4H), 3.46 (m, 2H), 3.37 (s, 3H); ^{13}C NMR (101 MHz, $CDCl_3$): 168.1 (C), 145.3 (C), 141.1 (C), 137.5 (CH), 128.4 (CH), 120.3 (CH_2), 93.3 (C), 74.8 (CH), 72.0 (CH_2), 70.3 (CH_2), 69.6 (CH_2), 59.2 (CH_3), 39.3 (CH_2).

Compound 34. To a solution of **47** (72 mg, 0.26 mmol) in acetic anhydride (1.4 mL) concentrated sulfuric acid (100 µL) was added. The mixture was stirred for 45 min and then poured into a cooled to 0 °C solution of 2 M NaOH (10 mL) and stirred for 30 min. The mixture

was extracted with CH_2Cl_2 (x3) and the combined extracts were washed with sat. aq. solution of $NaHCO_3$ (x5). The crude product of **49** (54 mg, 0.17 mmol) was used for the next step without further purification.

Allylic ester **49** (25.0 mg, 77.8 µmol) and DABCO (40.0 mg, 356 µmol) were dissolved in dry THF (1.00 mL) and stirred at 50 °C for 2 d. The reaction mixture was diluted with EtOAc and washed with water. Aqueous phase was extracted with EtOAc (x2), and the combined organic phases were dried over Na_2SO_4, filtered, and concentrated in *vacuo*. The crude product was purified by flash column chromatography (Scorpius Silica C_{18} 5.4 g, 6 mL/min, linear gradient 10 – 95% MeCN + 0.1% TFA in H_2O + 0.1% TFA) to yield product **34** as a colorless oil (4.90 mg, 14%, after two steps). IR (neat): 3291 (br), 3063 (w), 2923 (w), 2871 (w), 2820 (w), 1737 (s), 1657 (s), 1631 (s), 1530 (m), 1493 (w), 1448 (m), 1372 (m), 1354 (w), 1223 (s), 1104 (s), 1026 (m), 967 (w), 922 (w); ^{1}H NMR (500 MHz, $CDCl_3$): 7.39 – 7.30 (m, 4H), 7.33 – 7.26 (m, 1H), 6.78 (s, 1H), 6.08 (s, 1H), 4.88 (d, $^4J_{H-H}$ = 1.2 Hz, 2H), 3.48 – 3.39 (m, 4H), 3.40 – 3.32 (m, 4H), 3.30 (s, 3H), 2.10 (s, 3H); ^{13}C NMR (126 MHz, $CDCl_3$): 170.7 (C), 168.0 (C), 134.8 (C), 133.7 (C), 132.9 (CH), 128.7 (CH), 128.6 (CH), 128.6 (CH), 71.8 (CH_2), 70.3 (CH_2), 69.5 (CH_2), 66.3 (CH_2), 59.1 (CH_3), 39.3 (CH_2), 21.1 (CH_3); HRMS (ESI, +ve) calcd for $C_{17}H_{23}NO_5$ $[M+Na]^+$: 344.1469, found: 344.1452.

Compound 35 was synthesized and purified according to procedures described in reference [S5].

Compound 36. To a solution of **48** (88.0 mg, 217 µmol) in acetic anhydride (1.00 mL) concentrated sulfuric acid (100 µL) was added. The mixture was stirred for 45 min and then poured into a cold solution of 2M NaOH (10 mL) and stirred for 30 min. The mixture was extracted with CH_2Cl_2 (x3) and the combined extracts washed with sat. aq. solution $NaHCO_3$ (x5). The crude product **50** was used for the next step without further purification.

Allylic ester **50** and DABCO (250 mg, 2.24 mmol) were dissolved in dry THF (2.5 mL) and stirred at 50 °C for 16 h. The reaction mixture was diluted with EtOAc and washed with water. Aqueous phase was washed with EtOAc (x2), organic phases were combined, dried over Na_2SO_4, filtered, and concentrated in *vacuo*. The crude product was purified twice by flash column chromatography (Scorpius Silica C_{18} 20 g, 15 mL/min, linear gradient 10 – 95% MeCN + 0.1% TFA in H_2O + 0.1% TFA, then, Scorpius Silica C_{18} 5.4 g HP, 10 mL/min, linear gradient 10 – 80% MeCN + 0.1% TFA in H_2O + 0.1% TFA) to yield product **36** as a yellowish oil (8.00 mg, 8%). IR (neat): 3305 (br), 2925 (w), 2871 (w), 2816 (w), 1739 (s), 1658 (m), 1633 (m), 1508 (w), 1535 (m), 1483 (w), 1452 (w), 1370 (m), 1354 (w), 1227 (s), 1134 (w), 1103 (m), 1060 (w), 1025 (w), 1006 (w), 967 (w); ^{1}H NMR (400 MHz, $CDCl_3$): 7.79 – 7.68 (m, 2H), 7.65 (s, 1H), 7.17 – 7.08 (m, 2H), 6.95 (s, 1H), 4.92 (s, 2H), 3.69 – 3.54 (m, 8H), 2.13 (s, 3H); ^{13}C NMR (101 MHz, $CDCl_3$): 171.1 (C), 167.2 (C), 140.2 (C), 138.0 (CH), 134.2 (C), 131.0 (CH), 130.9 (CH), 95.3 (C), 72.0 (CH_2), 70.3 (CH_2), 69.7 (CH_2), 59.6 (CH_2), 59.2 (CH_3), 39.9 (CH_2), 21.1 (CH_3); HRMS (ESI, +ve) calcd for $C_{17}H_{22}INO_5$ [M+Na]$^+$: 470.0435, found: 470.0420.

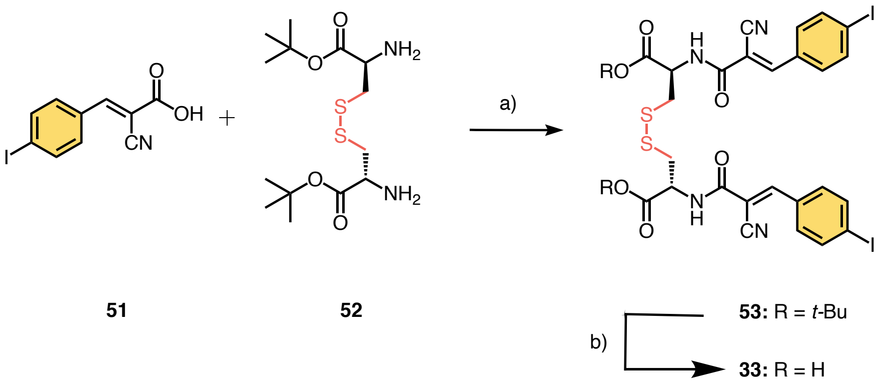

Scheme S3. (a) 1-chloro-*N,N*,2-trimethyl-1-propenylamine, DIPEA, CH_2Cl_2, 40 °C, 6 h, 5%; (b) TFA, CH_2Cl_2, rt, 2 h, quant.

Compound 51 was synthesized and purified according to procedures described in reference [S6].

Compound 53. Compound **51** (500 mg, 1.67 mmol) and 1-chloro-*N,N*,2-trimethyl-1-propenylamine (354 μL, 2.68 mmol) were mixed together in dry CH_2Cl_2 (25 mL). After stirring at rt for 45 min, a solution of amine **52** (292 mg, 685 μmol) and DIPEA (349 μL, 2.01 mmol) in CH_2Cl_2 (1 mL) was added. The reaction mixture was stirred at 40 °C for 6 h, and concentrated in *vacuo*. The crude product was purified by flash column chromatography (Scorpius Silica C18 12 g, 20 mL/min, linear gradient 30 – 90% MeCN + 0.1% TFA in H_2O + 0.1% TFA). The MeCN was evaporated and the product was extracted with EtOAc (x3). The combined organic phase was washed with brine (x1), dried over Na_2SO_4, filtered, and concentrated in *vacuo,* to result in a product **53** as a colorless oil (80 mg, 5%). The main product (64%) was the product of mono-addition. IR (neat): 3335 (br), 2324 (w), 2141 (w), 2045 (w), 1717 (m), 1668 (s), 1654 (s), 1598 (m), 1581 (m), 1536 (m), 1522 (s), 1485 (m), 1416 (m), 1405 (m), 1393 (w), 1376 (w), 1294 (m), 1188 (w), 1150 (w), 1063 (m), 1051 (w), 1039 (w), 1006 (m), 896 (w), 872 (w), 838 (w), 812 (w); ^{1}H NMR (400 MHz, $CDCl_3$): 8.19 (s, 2H), 7.81 (d, $^3J_{H-H}$ = 8.4 Hz, 4H), 7.61 (d, $^3J_{H-H}$ = 8.4 Hz, 4H), 7.22 (d, $^3J_{H-H}$ = 7.3 Hz, 2H), 4.86 (ddd, $^3J_{H-H}$ = 7.3 Hz, $^3J_{H-H}$ = 6.2 Hz, $^3J_{H-H}$ = 4.7 Hz, 2H), 3.36 (dd, $^2J_{H-H}$ = 14.2 Hz, $^3J_{H-H}$ = 4.7 Hz, 2H), 3.23 (dd, $^2J_{H-H}$ = 14.2 Hz, $^3J_{H-H}$ = 6.2 Hz, 2H), 1.50 (s, 18H); ^{13}C NMR (101 MHz, $CDCl_3$): 168.4 (C), 160.2 (C), 152.2 (CH), 138.7 (CH), 131.9 (CH), 131.1 (C), 116.3 (C), 104.5 (C), 100.6 (C), 84.0 (C), 53.9 (CH), 40.8 (CH_2), 28.1 (CH_3).

Compound 33. To a solution of **53** (10.0 mg, 10.9 μmol) in CH_2Cl_2 (0.5 mL), TFA (0.5 mL) was added. The reaction mixture was stirred at rt for 2 h. The solvent was removed *in vacuo*, resulting in the product **33** (8.6 mg, quant.). Mp: 123-124 °C; IR (neat): 3357 (br), 2976 (w), 2922 (w), 2213 (w), 1732 (s), 1674 (s), 1600 (m), 1578 (m), 1515 (s), 1487 (m), 1399 (w), 1368 (w), 1350 (w), 1251 (m), 1208 (w), 1189 (w), 1152 (s), 1121 (w), 1080 (w), 1060 (w), 1004 (m), 955 (w); ^{1}H NMR (500 MHz, DMSO-d_6): 8.83 (d, $^3J_{H-H}$ = 7.8 Hz, 2H), 8.12 (s, 2H), 7.94 (d, $^3J_{H-H}$ = 8.3 Hz, 4H), 7.67 (d, $^3J_{H-H}$ = 8.3 Hz, 4H), 4.65 (ddd, $^3J_{H-H}$ = 9.6

Hz, $^3J_{H\text{-}H}$ = 7.8 Hz, $^3J_{H\text{-}H}$ = 4.5 Hz, 2H), 3.33 – 3.25 (m, 2H), 3.09 (dd, $^2J_{H\text{-}H}$ = 13.9 Hz, $^3J_{H\text{-}H}$ = 9.6 Hz, 2H); ^{13}C NMR (126 MHz, DMSO-d_6): 171.3 (C), 161.0 (C), 150.4 (CH), 138.2 (CH), 132.6 (CH), 131.1 (C), 116.0 (C), 106.2 (C), 100.6 (C), 52.3 (CH), 38.6 (CH_2); HRMS (ESI, +ve) calcd for $C_{26}H_{20}N_4O_6S_2$ $[M+Na]^+$: 824.8806, found: 824.8823.

2.2. Synthesis of MAC Reporters

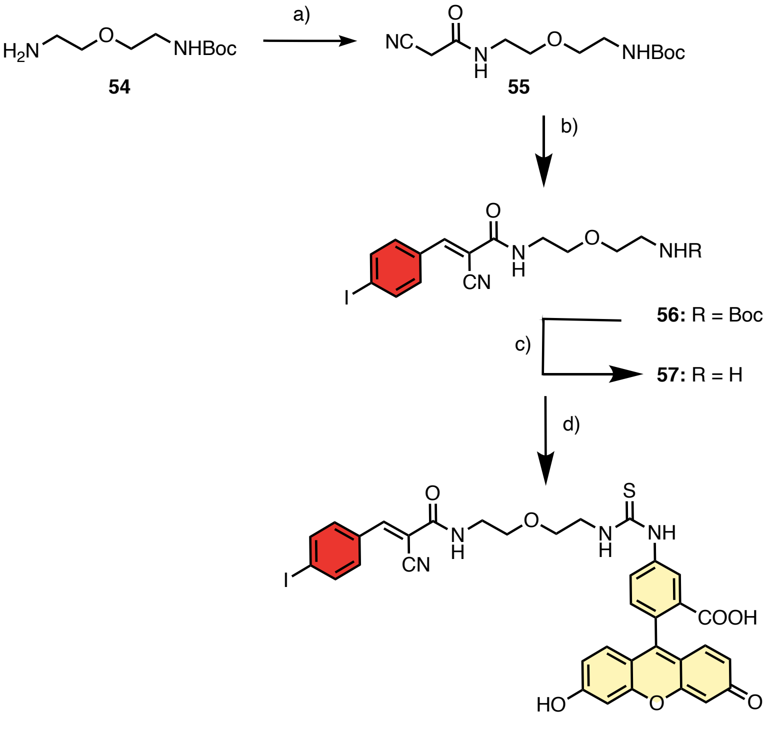

16

Scheme S4. (a) ethyl cyanoacetate, neat, rt, 18 h, quant.; (b) **41**, piperidine, EtOH, 60 °C, 5 h, 42%; (c) CH_2Cl_2/TFA, rt, 1 h; (d) DMF, TEA, fluorescein-5-isothiocyanate, rt, 3 h, 29% (two steps).

Compound 54 was synthesized and purified according to procedures described in reference [S7].

Compound 55. A mixture of **54** (150 mg, 734 µmol) and ethyl cyanoacetate (94.1 µL, 734 µmol) was stirred at rt for 18 h. Afterward, the product mixture was concentrated *in vacuo*, to afford product **55** as an orange oil (198 mg, quant.). The product **55** was used in the next

step without further purification. IR (neat): 3303 (w), 2977 (w), 2931 (w), 2876 (w), 2259 (w), 1670 (s), 1521 (s), 1457 (m), 1391 (m), 1366 (m), 1277 (m), 1250 (s), 1168 (s), 1119 (s), 1039 (w), 859 (w), 782 (w); ^{1}H NMR (400 MHz, $CDCl_3$): 6.87 (s, 1H), 4.85 (s, 1H), 3.59 – 3.48 (m, 6H), 3.41 (s, 2H), 3.35 – 3.27 (m, 2H), 1.45 (s, 9H); ^{13}C NMR (101 MHz, $CDCl_3$): 161.4 (C), 156.5 (C), 114.9 (C), 79.8 (C), 70.6 (CH_2), 69.2 (CH_2), 40.4 (CH_2), 40.1 (CH_2), 28.5 (3xCH_3), 25.9 (CH_2).

Compound 56. Compound **55** (45.0 mg, 166 µmol), and **41** (38.5 mg, 166 µmol) were mixed together with piperidine (100 µL) in EtOH (5 mL). The reaction mixture was stirred for 5 h at 60 °C. Then the solvent was evaporated, and the crude product was purified by flash column chromatography (Scorpius Silica 12 g, 45 mL/min, linear gradient 0 – 7% MeOH in CH_2Cl_2) to yield product **56** as a yellowish oil (34 mg, 42%). R_f (CH_2Cl_2/MeOH 95:5) = 0.75; IR (neat): 3344 (w), 2975 (w), 2930 (w), 2871 (w), 2213 (w), 1678 (s), 1608 (m), 1581 (m), 1523 (s), 1486 (m), 1394 (w), 1365 (w), 1275 (m), 1251 (m), 1169 (m), 1131 (m), 1063 (w), 1004 (w), 819 (w); ^{1}H NMR (400 MHz, $CDCl_3$): 8.24 (s, 1H), 7.89 – 7.81 (m, 2H), 7.68 – 7.60 (m, 2H), 6.75 (s, 1H), 4.90 (s, 1H), 3.64 – 3.59 (m, 4H), 3.55 (t, $^{3}J_{H\text{-}H}$ = 5.3 Hz, 2H), 3.34 (q, $^{3}J_{H\text{-}H}$ = 5.3 Hz, 2H), 1.44 (s, 9H); ^{13}C NMR (101 MHz, $CDCl_3$): 160.2 (C), 156.1 (C), 151.8 (CH), 138.8 (CH), 131.8 (CH), 131.3 (C), 116.8 (C), 104.9 (C), 100.3 (C), 79.6 (C), 70.4 (CH_2), 69.2 (CH_2), 40.5 (CH_2), 40.4 (CH_2), 28.5 (3xCH_3).

Compound 16. To a solution of **56** (34.0 mg, 70.1 µmol) in CH_2Cl_2 (1.00 mL) TFA (0.5 mL) was added. The reaction mixture was stirred at rt for 1 h. The solvent was removed *in vacuo*, resulting in product **57** as a TFA salt. The crude product was used in the next step without further purification. The solution of the **57** TFA salt in DMF (500 µL) was cooled down to 0 °C, and triethylamine (60.0 µL, 430 µmol), and fluorescein-5-isothiocyanate (27.3 mg, 70.1 µmol) were added. The reaction mixture was stirred at rt for 3 h. The crude product was directly purified by flash column chromatography (Scorpius Silica C18 5.4 g, 6 mL/min,

linear gradient 10 – 95% MeCN + 0.1% TFA in H_2O + 0.1% TFA). Fractions containing the desired products were lyophilized to afford product **16** as an orange solid (15.5 mg, 29%). Mp: 145-146 °C; IR (neat): 3036 (w), 2873 (w), 2576 (w), 2217 (w), 1664 (m), 1638 (m), 1578 (s), 1535 (s), 1454 (s), 1381 (m), 1272 (s), 1173 (s), 1117 (s), 1004 (w), 916 (w), 849 (w), 831 (w), 796 (w), 764 (w), 719 (w); ^{1}H NMR (500 MHz, DMSO-d_6): 10.11 (s, 1H), 9.99 (s, 1H), 8.50 (t, $^3J_{H\text{-}H}$ = 5.6 Hz, 1H), 8.27 (s, 1H), 8.12 (s, 1H), 8.08 (s, 1H), 7.96 – 7.90 (m, 2H), 7.71 (d, $^3J_{H\text{-}H}$ = 7.7 Hz, 1H), 7.69 – 7.66 (m, 2H), 7.15 (d, $^3J_{H\text{-}H}$ = 8.3 Hz, 1H), 6.66 (d, $^4J_{H\text{-}H}$ = 2.3 Hz, 2H), 6.60 – 6.53 (m, 4H), 3.69 (s, 2H), 3.62 (t, $^3J_{H\text{-}H}$ = 5.7 Hz, 2H), 3.57 (t, $^3J_{H\text{-}H}$ = 5.7 Hz, 2H), 3.48 – 3.40 (m, 2H); ^{13}C NMR (126 MHz, DMSO-d_6): 180.6 (C), 168.5 (C), 161.0 (C), 159.5 (C), 151.9 (C), 149.8 (CH), 147.2 (C), 141.3 (C), 138.2 (CH), 131.5 (CH), 131.3 (C), 129.4 (CH), 129.2 (C), 129.0 (CH), 126.5 (C), 124.0 (CH), 116.3 (C), 116.2 (CH), 112.6 (CH), 109.7 (C), 106.8 (C), 102.2 (CH), 100.3 (C), 68.4 (CH_2), 68.1 (CH_2), 43.7 (CH_2), 39.6 (CH_2); HRMS (ESI, +ve) calcd for $C_{35}H_{27}IN_4O_5S$ $[M+H]^+$: 775.0718, found: 775.0732.

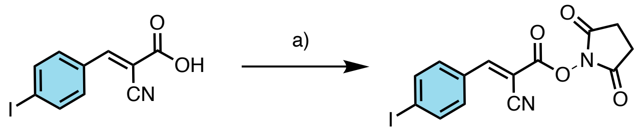

51 **22**

Scheme S5. (a) *N*-Hydroxysuccinimide, EDC·HCl, MeCN, rt, 16 h, 50%.

Compound 22. To the solution of **51** (48.0 mg, 161 μmol) in MeCN (1.6 mL), EDC·HCl (33.8 mg, 177 μmol) was added, after 10 min followed by the addition of *N*-hydroxysuccinimide (20.3 mg, 177 μmol). The reaction mixture was stirred at rt for 16 h. The organic solvent was concentrated in *vacuo* and diluted with CH_2Cl_2 (2 mL). Organic phase was washed with H_2O (x1), saturated solution of $NaHCO_3$ (x2), brine (x1), dried over Na_2SO_4, filtered, and concentrated in *vacuo*. The product was obtained as a 1/1 mixture of **51**/**22** detected by ^{1}H NMR. The product mixture was used for protein modification.

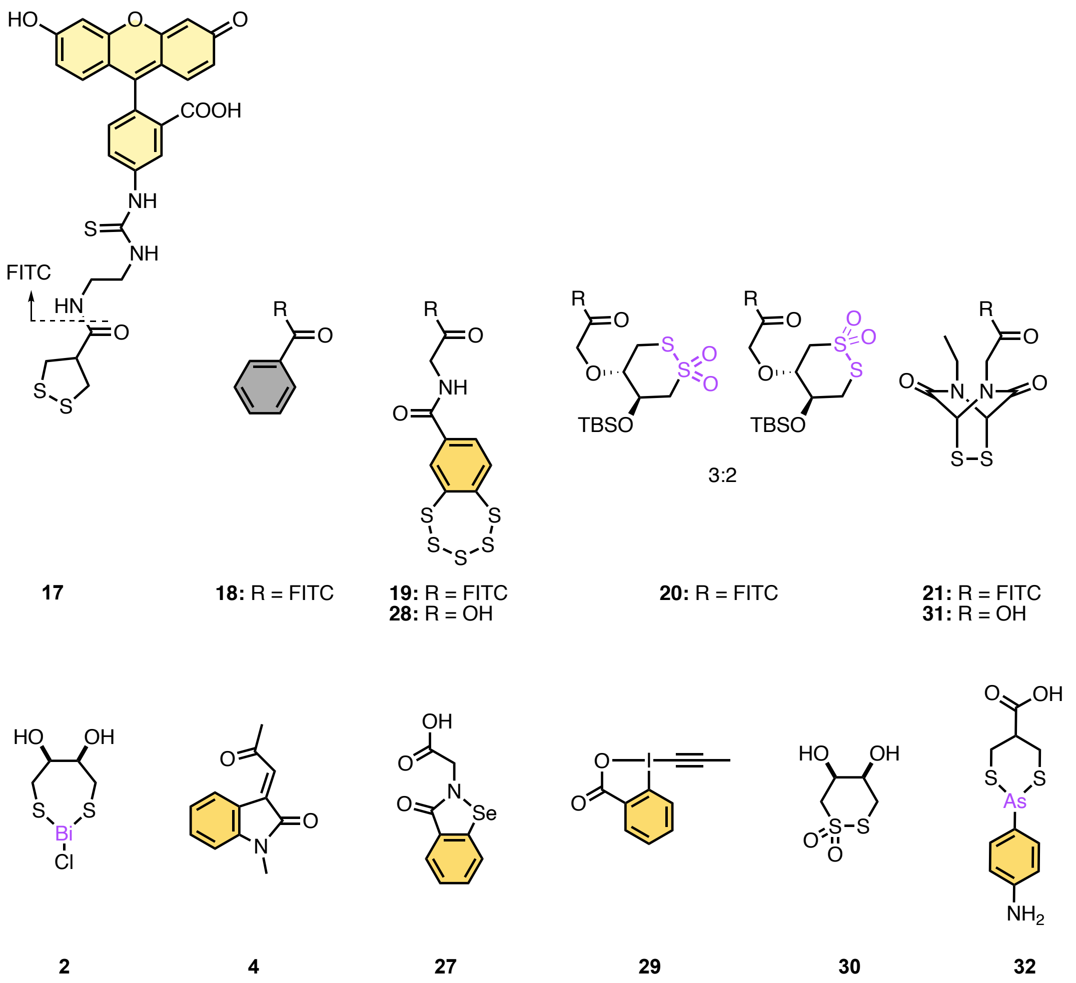

Figure S1. Structure of synthetic inhibitor and reporters.

Compounds 17, 21, 31 were synthesized and purified according to procedures described in reference [S8].

Compounds 18, 19, 28 were synthesized and purified according to procedures described in reference [S9].

Compounds 20, 4, 27, 29, 30 were synthesized and purified according to procedures described in reference [S10].

Compounds 2, 32 were synthesized and purified according to procedures described in reference [S11].

3. Cell Culture

As described in reference [S1]. Namely, human cervical cancer-derived HeLa Kyoto cells were cultured in DMEM (GlutaMAX, 4.5 g/L D-glucose, with phenol red) medium containing 10% fetal bovine serum (FBS) and 1% Penicillin/Streptomycin (PS). The cells were grown on a 25 cm^3 tissue culture flask (TPD Corporation) under 5% CO_2 humidified atmosphere at 37 °C. Cells were detached by treatment with 1.5 mL of TrypLE Express at 37 °C for 5 min, followed by the addition of 6 mL of DMEM (GlutaMAX, 4.5 g/L D-glucose, with phenol red) medium at 37 °C. The cells were resuspended in FluoroBrite DMEM + 10% FBS medium and plated according to the concentration needed.

4. Automated High-Content High-Throughput (AHCHT) Inhibitor Screening

4.1. General Procedure for AHCHT Inhibitor Screening with OPS Reporters

Co-Incubation Method. As described in reference [S10]. HeLa Kyoto cells were seeded at 8×10^4 cells/mL in FluoroBrite DMEM + 10% FBS on μ-Plate 96-well Black ibiTreat sterile (150 μL per well) and incubated at 37 °C with 5% CO_2 for 24 h. Then, the medium was removed and cells were washed with PBS (3 × 3 mL/well) and fresh FluoroBrite DMEM medium (4 × 150 μL/well) using a plate washer (Biotek EL406®), keeping a final volume of 135 μL/well. Different concentrations of the inhibitors (10× final concentration in PBS) were prepared from 50 mM stock solution in DMSO (with 0.4% final concentration of DMSO). These inhibitor solutions (10× in PBS), OPS **15** (10× in PBS), a solution of Hoechst 33342 (100 μg/mL) and Propidium Iodide (PI, 10 μg/mL) in PBS were prepared freshly in a 96-well V-bottom plate before adding to the cells. The inhibitor solutions from the V-bottom plate were added to the cells (15 μL/well) to give a final volume of 150 μL/well using an electronic multichannel pipette, and the cells were incubated for 1 h at 37 °C with 5% CO_2. After this incubation, the reporter **15** from the V-bottom plate was added (15 μL/well) to the cells at a final volume of 165 μL/well. A final concentration of reporter **15** was 500 nM except for the

control wells, where only PBS was added (15 µL/well). Cells treated with reporter **15** were incubated for 2 h at 37 °C with 5% CO_2. After that, the cells were washed with PBS and FluoroBrite DMEM using the plate washer, and the solution of Hoechst 33342 and PI from the V-bottom plate was added (15 µL/well) to the cells in a final volume of 150 µL/well. After 20 min of incubation at 37 °C with 5% CO_2, the cells were washed and kept in clean FluoroBrite DMEM. For live cell imaging, samples were kept at 37 °C with 5% CO_2 during the microscope imaging. The distribution of fluorophores in cells was analyzed using an IXM-C automated microscope. 9 images per well were acquired using a 20× objective lens, and fluorescent images were acquired in three channels (Figure S2), blue for Hoechst 33342 with 377/50 nm excitation filter and 477/60 nm emission filter, red for Cy5 with 620/50 nm excitation filter and 690/50 nm emission filter, and red for PI 531/40 nm excitation filter and 593/40 nm emission filter. Duplicates were performed for each condition.

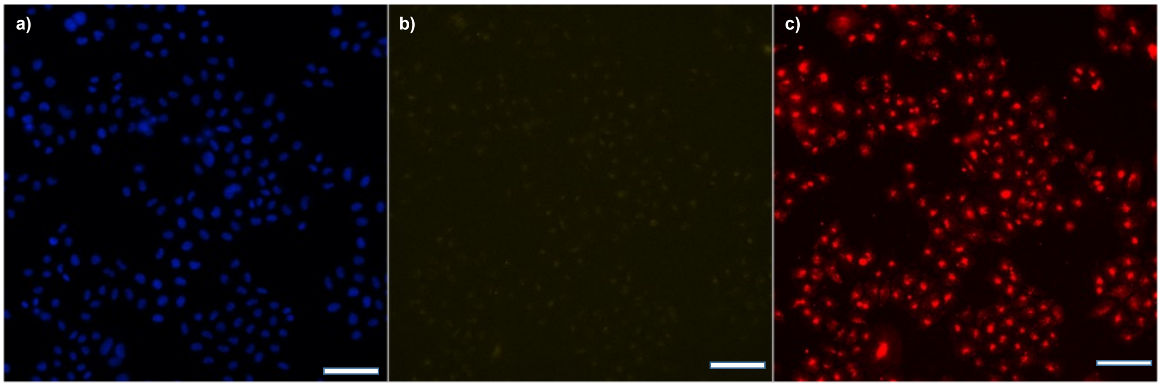

Figure S2. SDCM images of a) blue channel - Hoechst 33342 Nuclei DNA stain; b) red channel – PI; c) far-red channel - Cy5-labelled OPS **15**. Scale bar = 100 µm.

4.2. Data Analysis for AHCHT Inhibitor Screening with OPS Reporters

SDCM images resulting from the cellular uptake experiment and inhibitor screening using reporter **15** were automatically analyzed and quantified using a protocol adapted from reference [S12].

For the analysis, the nuclei and a cell body were segmented using the blue channel image. This segmentation generated the nuclei and cell masks (Figure S3).

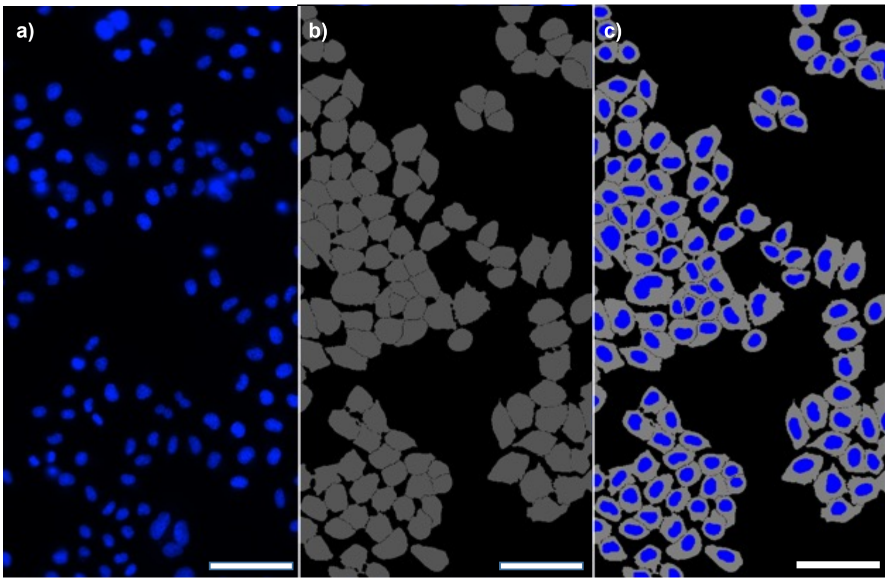

Figure S3. a) SDCM images of the blue channel - Hoechst 33342; b) cell body mask; c) overlay of nuclei and cell masks. Scale bar = 100 μm.

The cells with rounded shapes (shape factor > 0.8) were identified as dividing or dying cells, for which the reporter signal can be affected, and thus, filtered out (Figure S4).

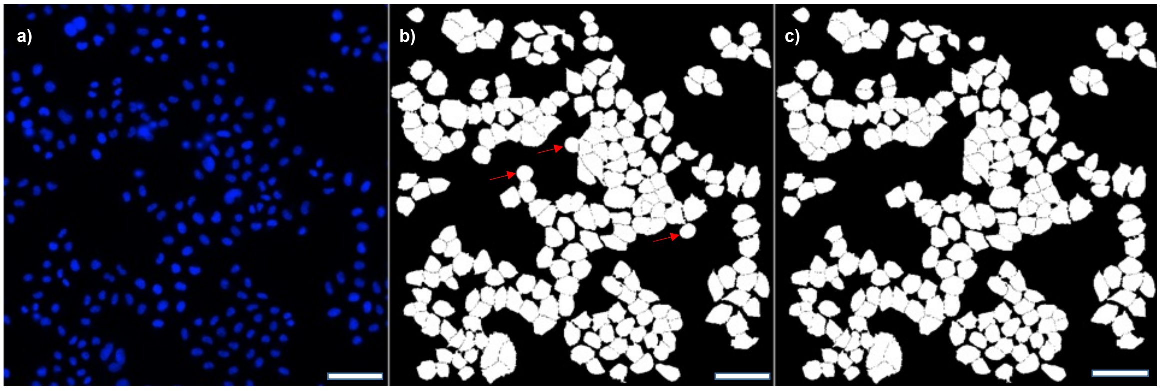

Figure S4. a) SDCM images of the blue channel - Hoechst 33342 localization in the nuclei; b) cell body mask with red arrows pointing to the rounded cells; c) cell mask after filtering. Scale bar = 100 μm.

We removed the OPS intense precipitates, that would create artifacts and perturb the proper quantification of the signal in the cell, we segmented the bright aggregates, observed in Cy5 channel (Figure S5a). The bright object mask generated was then filtered based on the maximum signal intensity with threshold of 30000 (Figure S5b). The resulting masks were then grown and all cells in contact with those were removed from the analysis (Figure S5c-e).

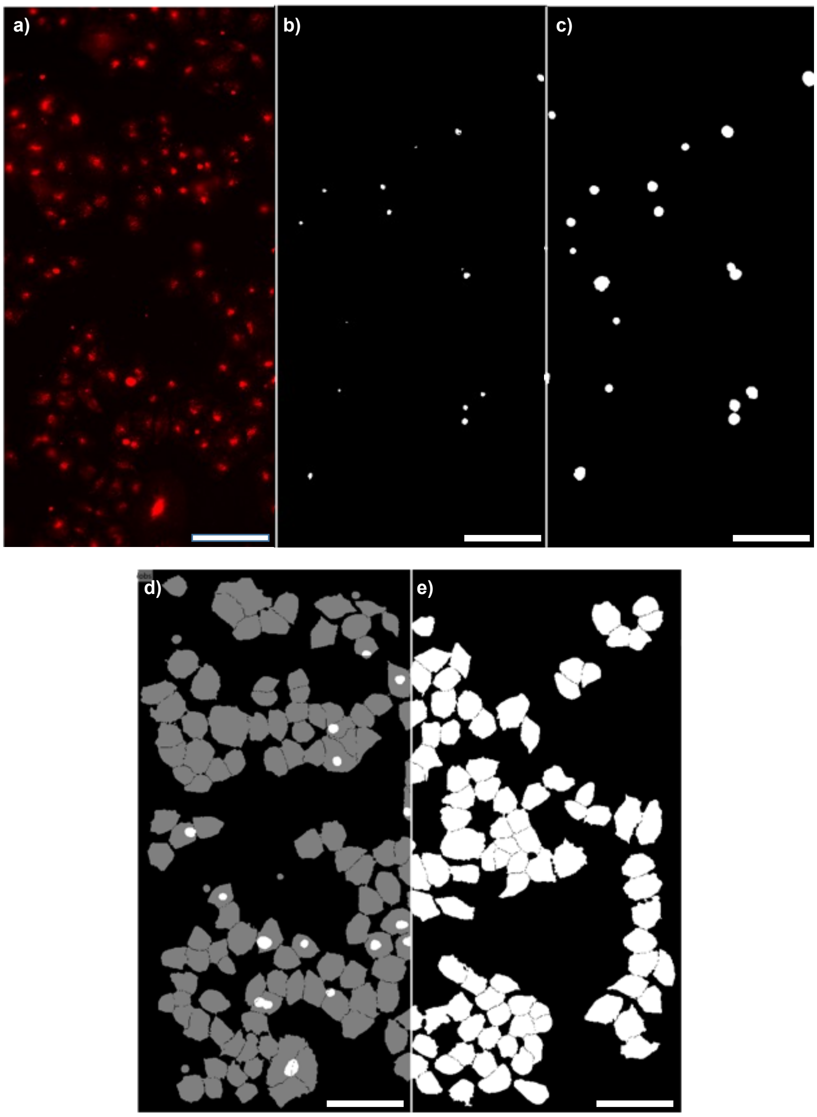

Figure S5. a) SDCM images of far-red channel OPS signal; b) segmented bright fluorescent aggregates; c) grown aggregates; d) overlay of cell mask with detected aggregates; e) final cell mask. Scale bar = 100 μm.

To quantify the fluorescence intensity of the OPS reporter, dotted structures were detected. To facilitate the detection of the OPS dots, *Top-hat* transformation of the Cy5 red channel image was applied (Figure S6b). The modified image was used to segment the round objects (Figure S6c). The round dot mask was kept only in final cell mask (Figure S6d-e).

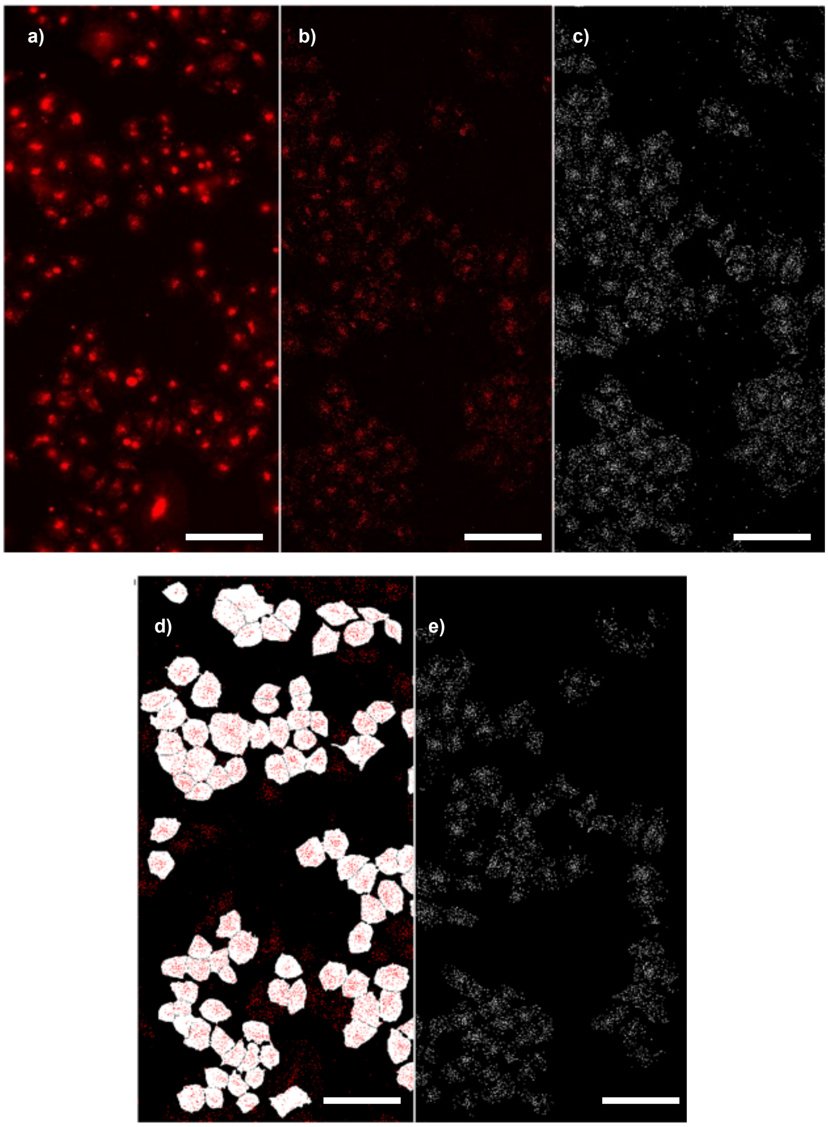

Figure S6. SDCM images of a) far-red channel with OPS **15**; b) *top-hat* transformation; c) round dot mask; d) overlay of round dot mask with cell mask; e) final round dot mask. Scale bar = 100 µm.

Background mask was generated by removing all cell mask (cell mask before filtering (Figure S3)) grown and the artifact mask from the image mask (Figure S7).

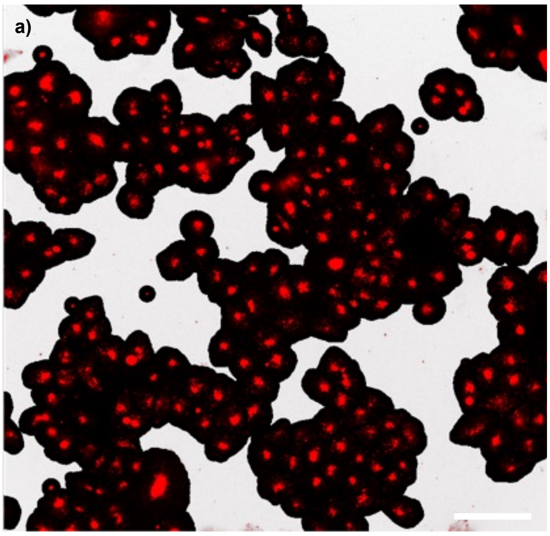

Figure S7. SDCM image of background mask (light grey). Scale bar = 100 µm.

The final masks were then applied to the far-red channel for quantification (Figure S8).

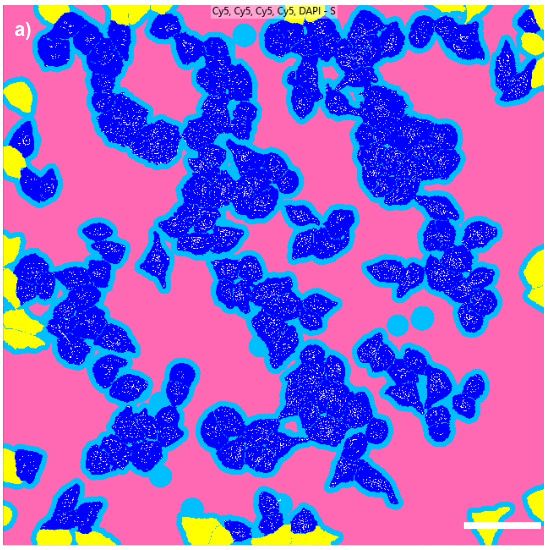

Figure S8. Final mask. White dots – OPS, dark blue – cell, light blue – Image mask in the back, yellow – border cells removed for intensity quantification but used for cell viability count, pink – background. Scale bar = 100 µm.

The fluorescent intensity per cell was quantified using total integrated intensity (I_x: sum of pixel intensity in the object) of OPS round dots for the whole image divided by the number of cells (n.c.) on the image, resulting in fluorescent intensity values per cell $I_{pc} = I_x$ /n.c.

I_{pc} values in the presence of inhibitors were normalized relative to the same values obtained without the inhibitor and without the transporter as 1 and 0, respectively. The resulting dependence of the relative fluorescent intensity values (I_{rel}) to the concentration of inhibitors ($c_{inhibitor}$) was plotted and fitted with Equation (S1) to retrieve the half maximal inhibitory concentration (IC_{50}) and the Hill coefficient (n). MIC values were estimated from the fit curve as the concentration at which 15% of uptake was inhibited.

$$I_{rel} = 1 / (1 + (IC_{50} / c_{inhibitor})^{-n}) \qquad (S1)$$

Relative cell viability (RV) for each condition in the presence of inhibitors was calculated as the count of Hoechst 33342 stained cells minus the count of PI stained cells divided by the count of Hoechst 33342 stained cells for each set of experiments. The resulting dependence of the relative cell viability (RV) to the concentration of inhibitors ($c_{inhibitor}$) was plotted and fitted with Equation (S2) to retrieve the concentration causing 50% cell growth inhibition (RV_{50}) value and the Hill coefficient (n).

$$RV = 1 / (1 + (RV_{50} / c_{inhibitor})^{-n}) \qquad (S2)$$

4.3. Results for AHCHT Inhibitor Screening with OPS Reporters

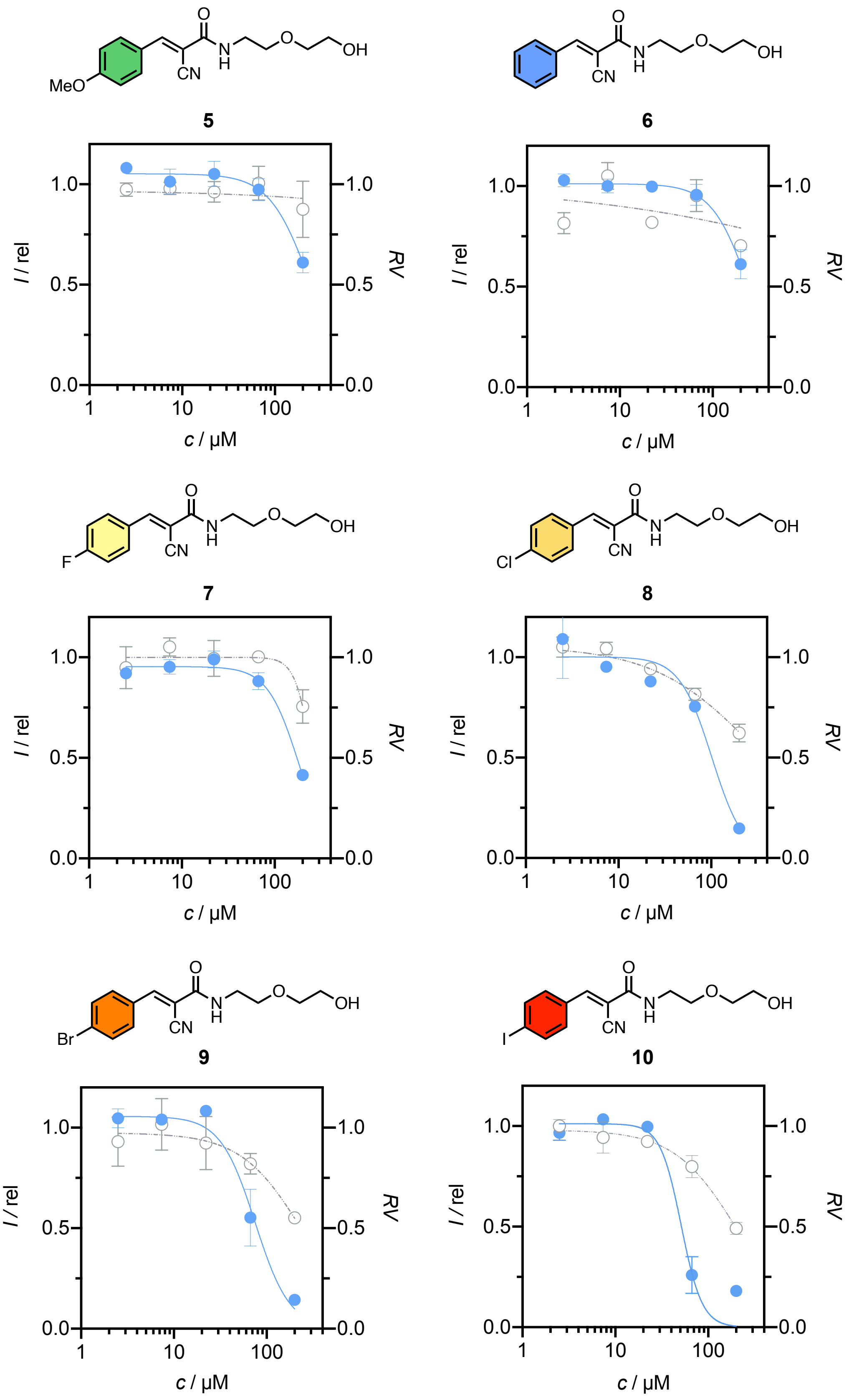

Figure S9. (continued)

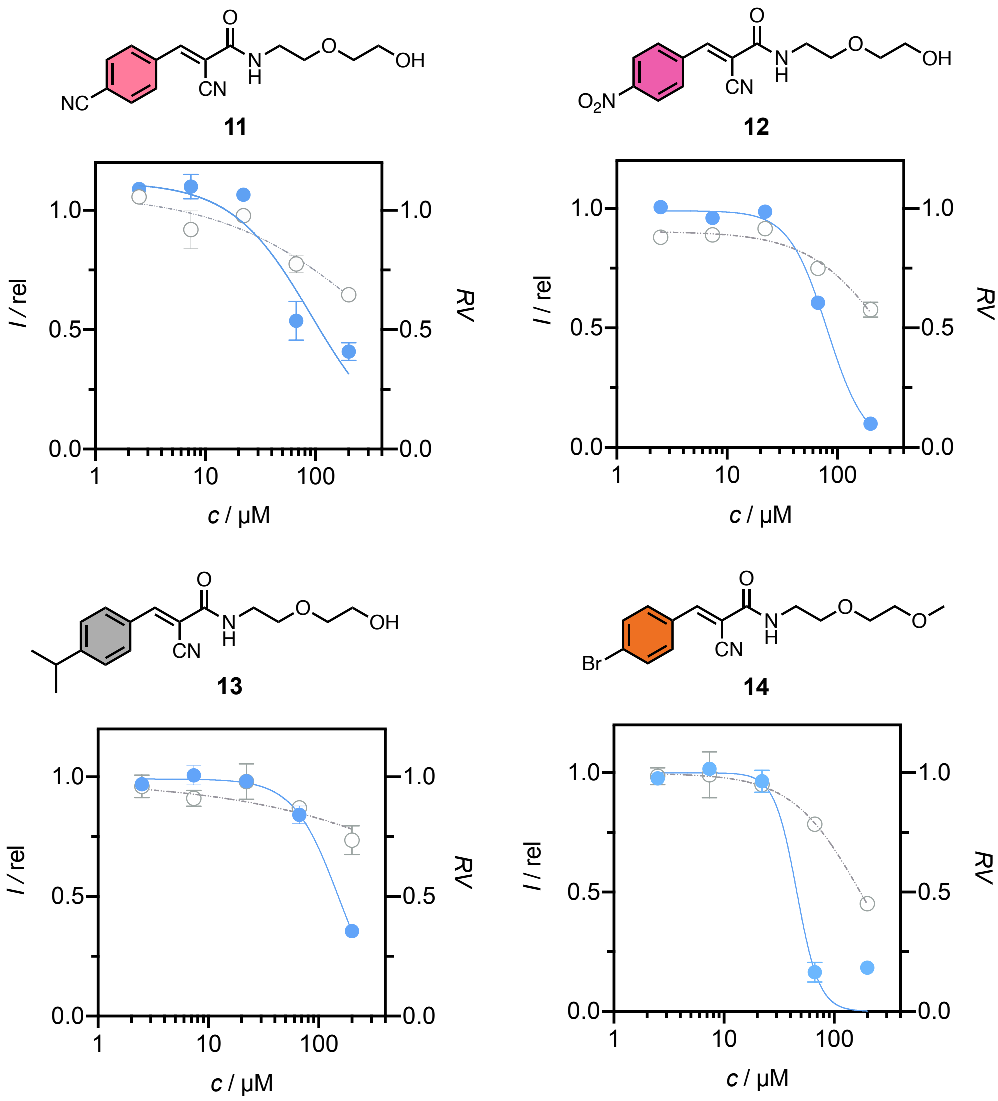

Figure S9. AHCHT data showing relative fluorescence intensity (blue circles) and relative viability (empty grey circles) of HeLa Kyoto cells after incubation with inhibitor for 1 h followed by coincubation with OPS **15** (500 nM) for 2 h.

Table S1. Inhibition of cellular uptake of OPS **15** and cell viability (coincubation experiment).

Compound	MIC[a] (μM)	IC_{50}[b] (μM)	*n* (IC_{50})[c]	RV_{50}[d] (μM)	*n* (RV_{50})[e]
**5**	120	240 ± 30	2.0 ± 0.7	>200	-
**6**	110	245 ± 25	2.2 ± 0.7	>200	-
**7**	80	180 ± 10	2.6 ± 0.6	>200	-
**8**	50	100 ± 20	2.5 ± 0.7	>200	-
**9**	40	80 ± 10	2.3 ± 0.6	>200	-
**10**	35	50 ± 10	3.9 ± 2.0	≈200	1.3 ± 0.4
**11**	35	90 ± 20	1.2 ± 0.4	>200	-
**12**	40	80 ± 5	2.5 ± 0.2	>200	-
**13**	60	150 ± 10	2.1 ± 0.2	>200	-
**14**	30	50 ± 10	4.3 ± 1.8	≈170	1.4 ± 0.3

[a]Concentration needed to reach 15% inhibition. [b]Concentration needed to reach 50% inhibition. [c]Hill coefficient for inhibition of cellular uptake. [d]Concentration needed to lower relative viability (RV) by 50%. [e]Hill coefficient for cell viability.

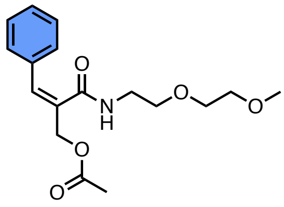

34

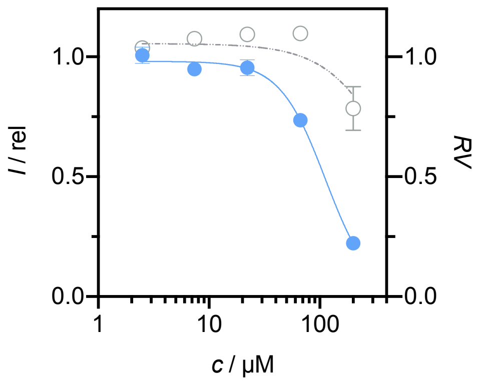

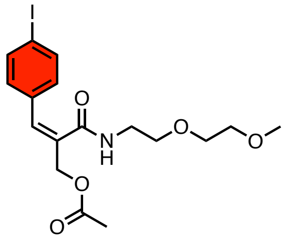

36

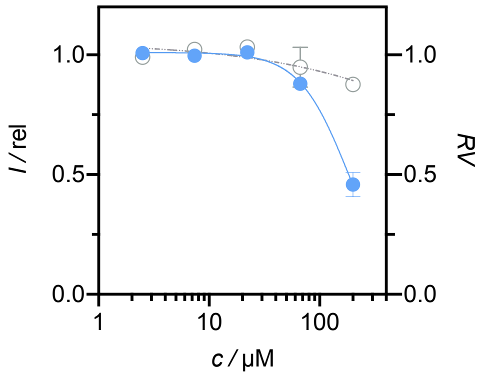

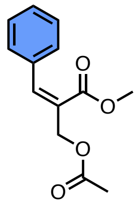

35

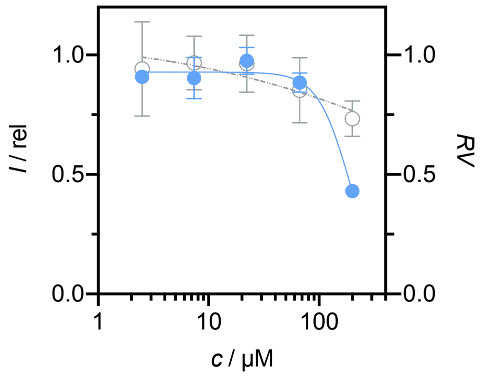

Figure S10. AHCHT data showing relative fluorescence intensity (blue circles) and relative viability (empty grey circles) of HeLa Kyoto cells after incubation with inhibitor for 1 h followed by coincubation with OPS **15** (500 nM) for 2 h.

Table S2. Inhibition of cellular uptake of OPS **15** and cell viability (coincubation experiment).

Compound	MIC[a] (µM)	IC_{50}[b] (µM)	*n* (IC_{50})[c]	RV_{50}[d] (µM)
**34**	45	110 ± 5	2.1 ± 0.2	>200
**35**	85	190 ± 15	3.0 ± 1.4	>200
**36**	80	180 ± 10	2.0 ± 0.2	>200

[a]Concentration needed to reach 15% inhibition. [b]Concentration needed to reach 50% inhibition. [c]Hill coefficient for inhibition of cellular uptake. [d]Concentration needed to lower relative viability (RV) by 50%.

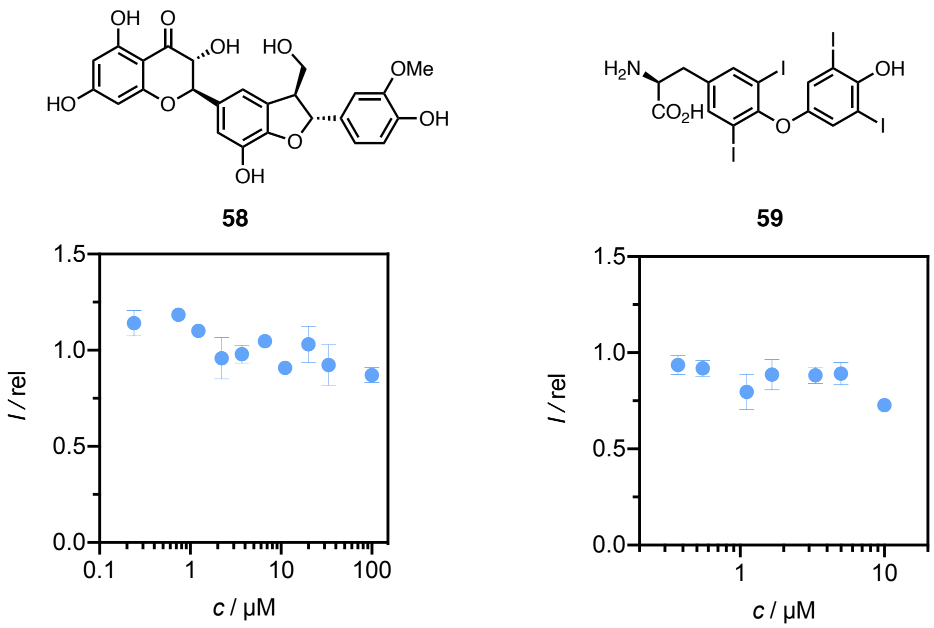

Figure S11. AHCHT data showing relative fluorescence intensity (blue circles) of HeLa Kyoto cells after incubation with inhibitors for 1 h followed by coincubation with OPS **15** (500 nM) for 2 h.

Table S3. Inhibition of cellular uptake of OPS **15** (coincubation experiment).

Compound	MIC[a] (μM)	IC_{50}[b] (μM)
**58**	>100	>100
**59**	>10	>10

[c]Concentration needed to reach 15% inhibition. [b]Concentration needed to reach 50% inhibition.

4.4. Cellular Uptake of MAC Reporters

Following the general procedure in reference [S1]. HeLa Kyoto cells were seeded at 8 $\times 10^4$ cells/mL in FluoroBrite DMEM + 10% FBS on μ-Plate 96-well Black ibiTreat sterile (150 μL per well) and incubated at 37 °C with 5% CO_2 for 24 h. Then, the medium was removed and cells were washed with PBS (3 × 3 mL/well) and fresh FluoroBrite DMEM medium (4 × 150 μL/well) using a plate washer (Biotek EL406®), keeping a final volume of 135 μL/well. Different concentrations of the reporter **16**, **17**, **18** and **19** (10× final concentration in PBS), were prepared from stock solution in DMSO, (with 1% final concentration of DMSO). These reporter solutions (10× in PBS), a solution of Hoechst 33342 (100 μg/mL) and PI (10 μg/mL) in PBS were prepared freshly in a 96-well V-bottom plate before adding to the cells. The reporter solution **16**, **17**, **18**, **19** from the V-bottom plate was added (15 μL/well) to the cells to give a final volume of 150 μL/well. Final concentrations of reporter **16** were 0, 5, 10, 20, 50 μM, of reporters **17** and **18** were 50 μM, and of reporter **19** was 10 μM. Cells treated with reporter **16** were incubated for 0.5, 1, and 1.5 h, with **17** and **18** for 1 h, and **19** for 1.5 h, at 37 °C with 5% CO_2. Afterward, the cells were washed with PBS and DMEM using the plate washer, and the solution of Hoechst 33342 and PI from the V-bottom plate was added (15 μL/well) to the cells in a final volume of 150 μL/well. After 20 min of incubation at 37 °C with 5% CO_2, the cells were washed and kept in clean FluoroBrite DMEM. For live cell imaging,

samples were kept at 37 °C with 5% CO_2 during the microscope imaging. The distribution of fluorophores in cells was analyzed using an IXM-C automated microscope. 4 images per well were acquired using 10× objective lens, and fluorescent images were acquired with three channels, blue for Hoechst 33342 with 377/50 nm excitation filter and 477/60 nm emission filter, green for FITC transporter with 475/34 nm excitation filter and 536/40 nm emission filter, and red for PI 531/40 nm excitation filter and 593/40 nm emission filter. SDCM images resulting from the cellular uptake experiment using FITC labeled reporters **16**, **17**, **18**, and **19** were automatically analyzed and quantified as described in the protocol in section 4.6.

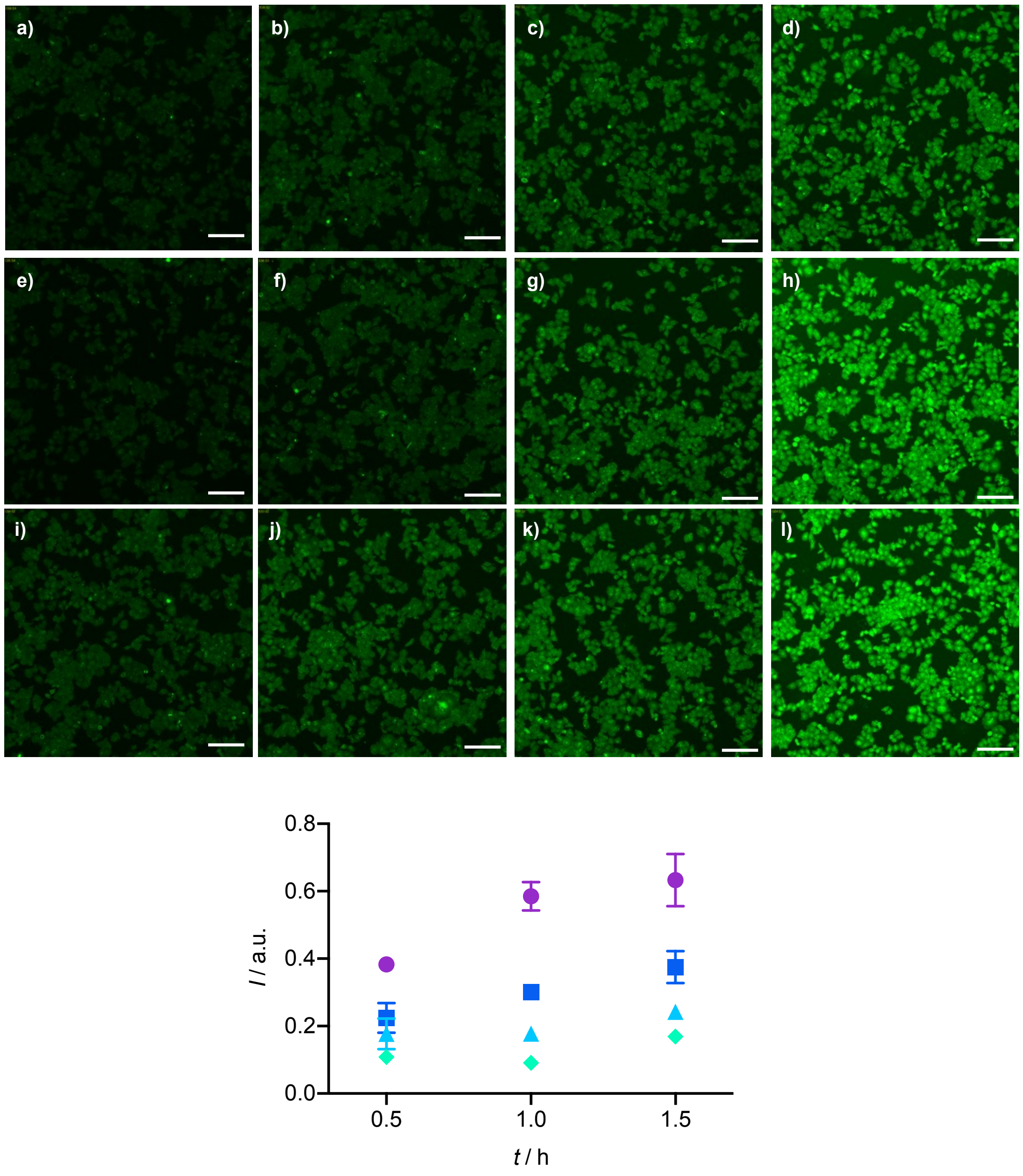

Figure S12. SDCM images of MAC **16** of HeLa Kyoto cells after 5, 10, 20 and 50 µM incubation for 0.5 h (a–d), 1 h (e–h), or 1.5 h (i–l) at 37 °C, scale bar = 200 µm. AHCHT data showing corrected to the background fluorescence intensity obtained from a) – i) with 5 µM (turquoise), 10 µM (light blue), 20 µM (dark blue) or 50 µM (purple).

4.5. General Procedure for AHCHT Inhibitor Screening with MAC Reporters and Controls

Pre-Incubation Method. As described in reference [S1]. HeLa Kyoto cells were seeded at 8 ×10^4 cells/mL in FluoroBrite DMEM + 10% FBS on µ-Plate 96-well Black ibiTreat sterile (150 µL per well) and incubated at 37 °C with 5% CO_2 for 24 h. Then, the medium was removed and cells were washed with PBS (3 × 3 mL/well) and fresh FluoroBrite DMEM medium (4 × 150 µL/well) using a plate washer (Biotek EL406®), keeping a final volume of 135 µL/well. Different concentrations of the inhibitors (10× final concentration in PBS) were prepared from stock solution in DMSO. These inhibitor solutions (10× in PBS), reporters **16**, **17**, **18** (10× in PBS), and reporter **20** (10x in DMEM), a solution of Hoechst 33342 (100 µg/mL in PBS) and PI (10 µg/mL in PBS) were prepared freshly in a 96-well V-bottom plate before adding to the cells. The inhibitor solutions from the V-bottom plate were added to the cells (15 µL/well) to give a final volume of 150 µL/well using an electronic multichannel pipette, and the cells were incubated for 1 h at 37 °C with 5% CO_2. After this incubation, cells were washed again with PBS and DMEM using the plate washer (in the way mentioned above), and reporter **16**, or **20** from the V-bottom plate was added (15 µL/well) to the cells at a final volume of 150 µL/well. A final concentration of reporter **16** was 10 µM, and **20** was 5 µM except for the control wells, where only PBS was added (15 µL/well). Cells treated with reporter **16** or **20** were incubated for 30 min, at 37 °C with 5% CO_2. Afterward, the cells were washed with PBS and DMEM using the plate washer, and the solution of Hoechst 33342 and PI from the V-bottom plate was added (15 µL/well) to the cells in a final volume of 150 µL/well. After 20

min of incubation at 37 °C with 5% CO_2, the cells were washed and kept in clean FluoroBrite DMEM. For live cell imaging, samples were kept at 37 °C with 5% CO_2 during the microscope imaging. The distribution of fluorophores in cells was analyzed using an IXM-C automated microscope. 4 images per well were acquired using a 10× objective lens, and fluorescent images were acquired with three channels, blue for Hoechst 33342 with 377/50 nm excitation filter and 477/60 nm emission filter, green for FITC transporter with 475/34 nm excitation filter and 536/40 nm emission filter, and red for PI 531/40 nm excitation filter and 593/40 nm emission filter.

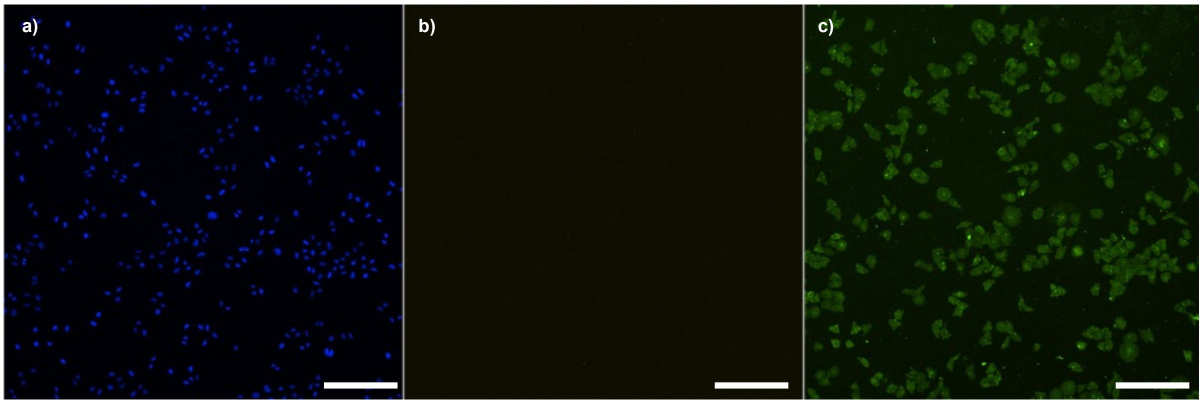

Figure S13. SDCM images of a) blue channel - Hoechst 33342 localization in the nuclei; b) red channel – PI; c) green channel – FITC labeled reporter **16** (10 µM). Scale bar = 200 µm.

Co-Incubation Method. The overall process is the same as a pre-incubation method, except for the washing process of cells after incubation with inhibitors. After incubation with inhibitors for 1 h, the transporter was added immediately to the cells without washing the plates.

4.6. Data Analysis for Uptake and Inhibition of MAC Reporters and Controls

SDCM images resulting from the cellular uptake experiment and inhibitor screening using FITC labeled reporters **16**, **17**, **18**, and **20** were automatically analyzed and quantified as described protocol in reference [S10]. Briefly, the analysis was similar to that described in section 4.2. The cells with rounded shapes, reporter precipitates, border objects were removed. Here the segmentation process was done using only cell mask and background mask (Figure S15).

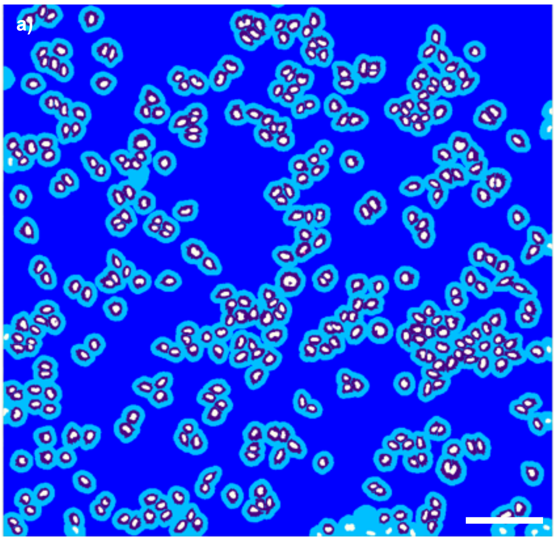

Figure S14. Final mask. White - nuclei, violet - cytosol, light blue – removed objects, dark blue - background. Scale bar = 200 µm.

The fluorescent intensity per cell was quantified using the average intensity of FITC reporters in cytosol after background subtraction. The normalization and data extraction were performed as described in section 4.2.

4.7. Results for AHCHT Inhibitor Screening with MAC Reporters

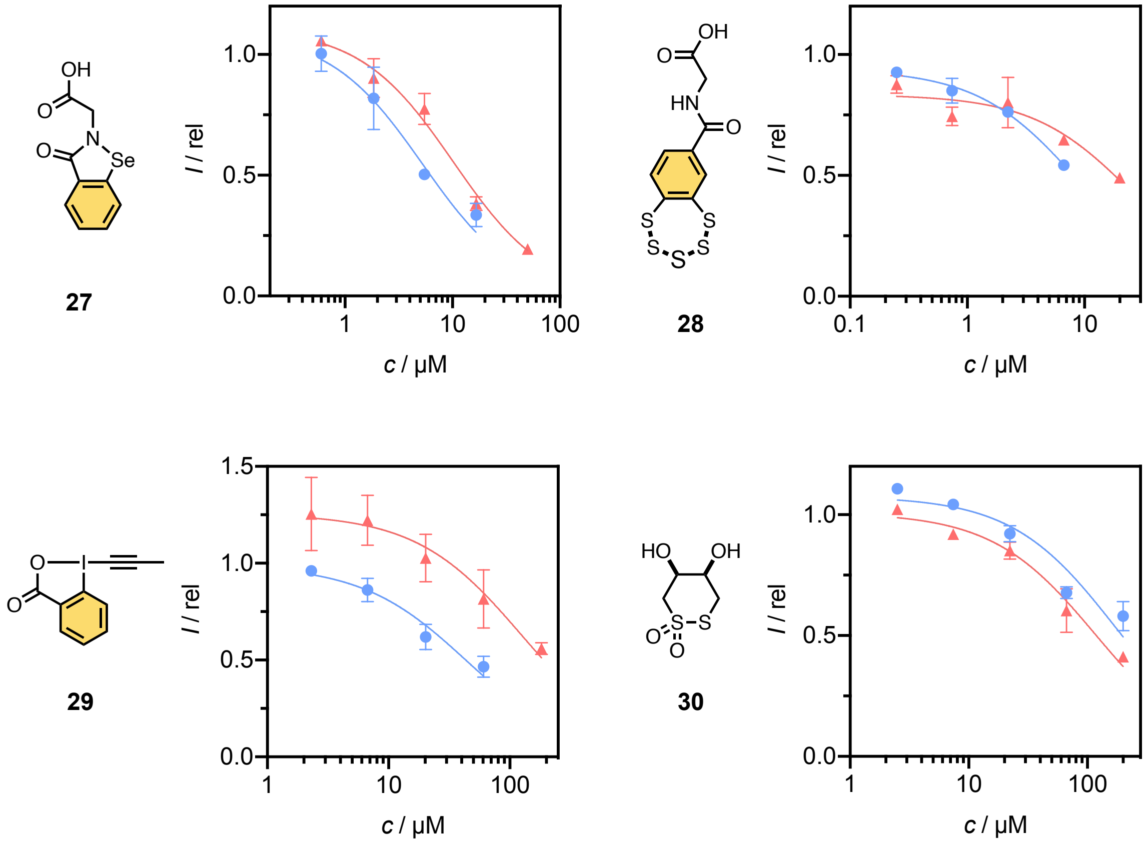

Figure S15. AHCHT data showing relative fluorescence intensity of HeLa Kyoto cells after incubation with each compound for 1 h followed by coincubation (blue circles) and preincubation (red triangles) with MAC **16** (10 µM) for 30 min. RV was reported in the reference [S10].

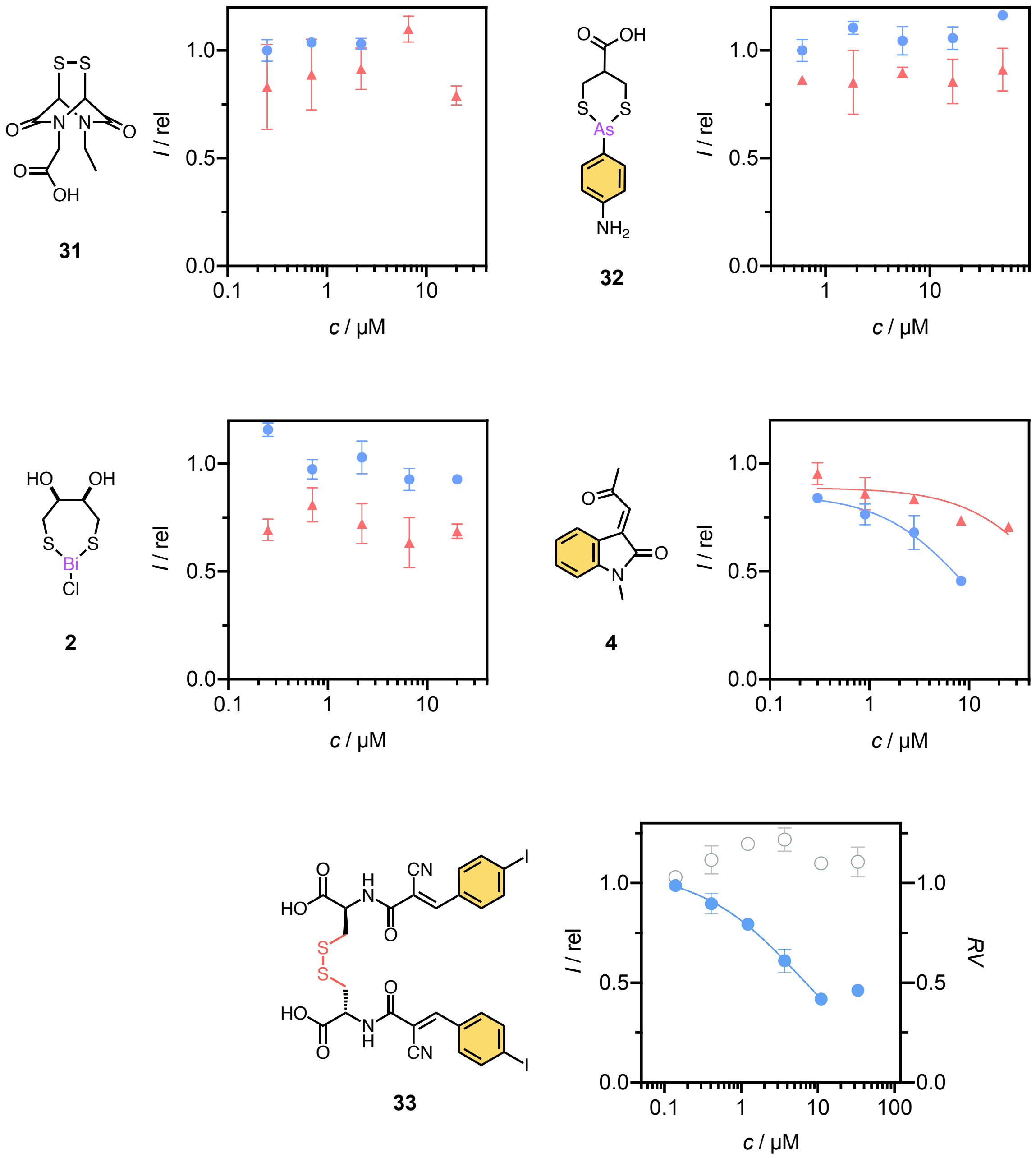

Figure S16. AHCHT data showing relative fluorescence intensity of HeLa Kyoto cells after incubation with each compound for 1 h followed by coincubation (blue circles), preincubation (red triangles), and relative viability (empty grey circles), with MAC **16** (10 μM) for 30 min. RV was reported in the reference [S10].

Table S4. Inhibition of cellular uptake of MAC reporter **16**.

Compound	Cond[a]	MIC[b,c] (µM)	IC_{50}[c,d] (µM)
**27**	C	1.5	6 ± 2
**28**	C	1	9 ± 3
**29**	C	5	40 ± 10
**30**	C	30	170 ± 40
**31**	C	>20	>20
**32**	C	>50	>50
**2**	C	>20	>20
**4**	C	0.5	10 ± 3
**33**	C	<1	6 ± 2
**27**	P	3	10 ± 2
**28**	P	<1	30 ± 10
**29**	P	60	150 ± 50
**30**	P	20	120 ± 20
**31**	P	>20	>20
**32**	P	>50	>50
**2**	P	>20	>20
**4**	P	<1	>25

[a]Conditions; P = pre-incubation, C = co-incubation. [b]Concentration needed to reach 15% inhibition. [c]Hill coefficient n = 1 was used. [d]Concentration needed to reach 50% inhibition.

4.8. Results for AHCHT Inhibitor Screening with CTO Reporters

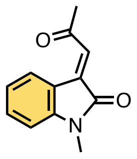

4

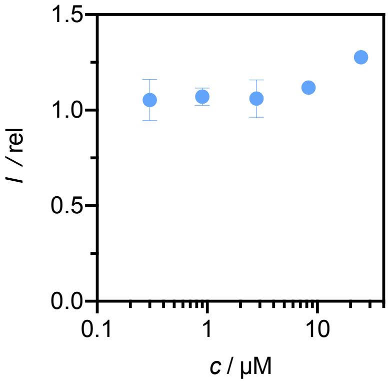

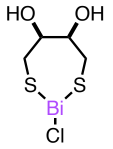

2

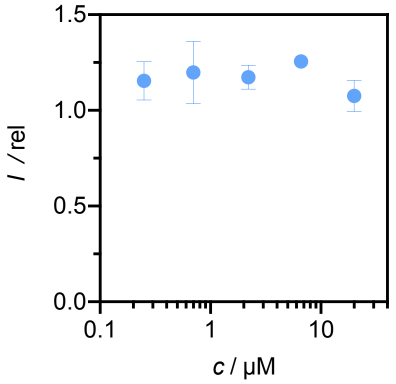

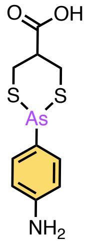

32

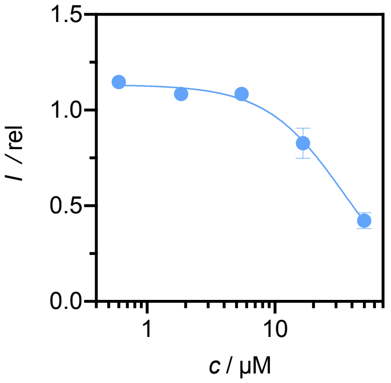

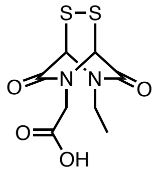

31

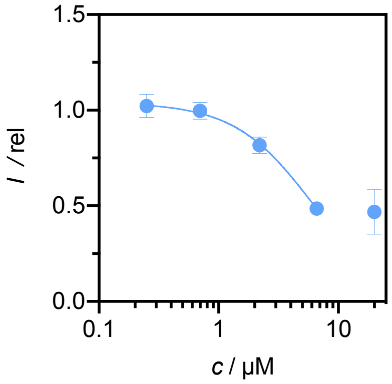

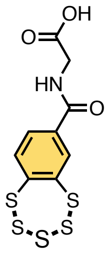

28

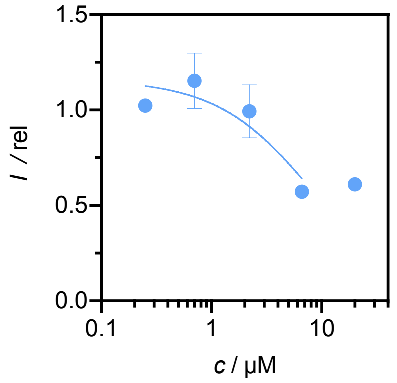

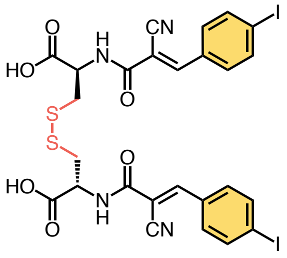

33

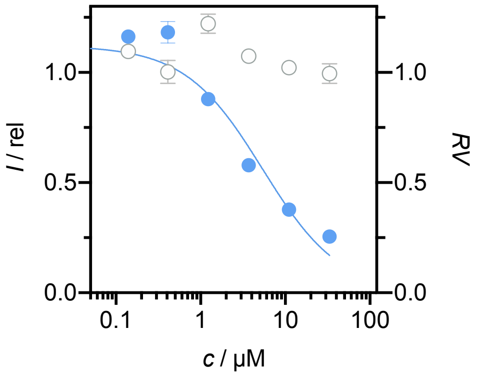

Figure S17. AHCHT data showing relative fluorescence intensity of HeLa Kyoto cells after incubation with each compound for 1 h followed by coincubation (blue circles), and relative viability (empty grey circles) with compound **20** (5 μM) for 30 min. RV was reported in the reference [S10]. Inhibition with compound **30** was reported in the reference [S10].

Table S5. Inhibition of cellular uptake of CTO reporter **20** (coincubation experiment).

Compound	MIC[a] (μM)	IC_{50}[b] (μM)	*n* (IC_{50})[c]
**31**	2	6 ± 1	1.3 ± 0.4
**32**	16	34 ± 6	1.4 ± 0.4
**33**	1.5	5 ± 2	1.0 ± 0.3
**2**	>20	>20	-
**28**	4	8 ± 4	1
**4**	>25	>25	-

[a]Concentration needed to reach 15% inhibition. [b]Concentration needed to reach 50% inhibition. [c]Hill coefficient for inhibition of cellular uptake.

5. Protein Uptake

5.1. Preparation of Streptavidin Complexes

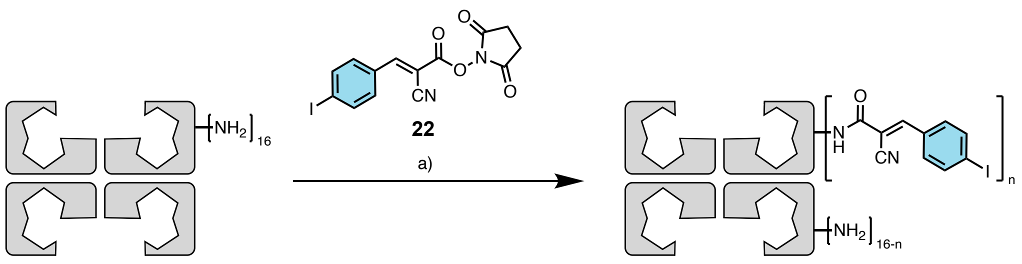

23 **60**

Scheme S6. (a) **22**, $NaHCO_3$ buffer, rt, 16 h.

Complex 60. As in reference [S13]. To the solution of **23** (WTS tetramer, 30 µM in freshly prepared 0.1 M $NaHCO_3$ buffer, pH = 8.3) 20 eq. of compound **22** (freshly prepared 20 mM in DMSO) was added. The resulting mixture was shaken (1000 rpm) for 16 h at rt. Sample was purified by passing through a desalting column equilibrated with PBS (PD midiTrap G-25, cytiva), and concentrated in a centrifugal cutoff filter (Amicon® Ultra 2 mL, 30 K). The final concentration of **60** was calculated by Bradford assay. Number of functionalized lysines up to n ≈ 8 per WTS tetramer, was determined using MALDI-TOF MS (Figure S19). M + ≈220 peaks can be attributed to sinapic acid adducts, M + ≈65 peaks to cyanoacetamide conjugates (retro-aldol product).

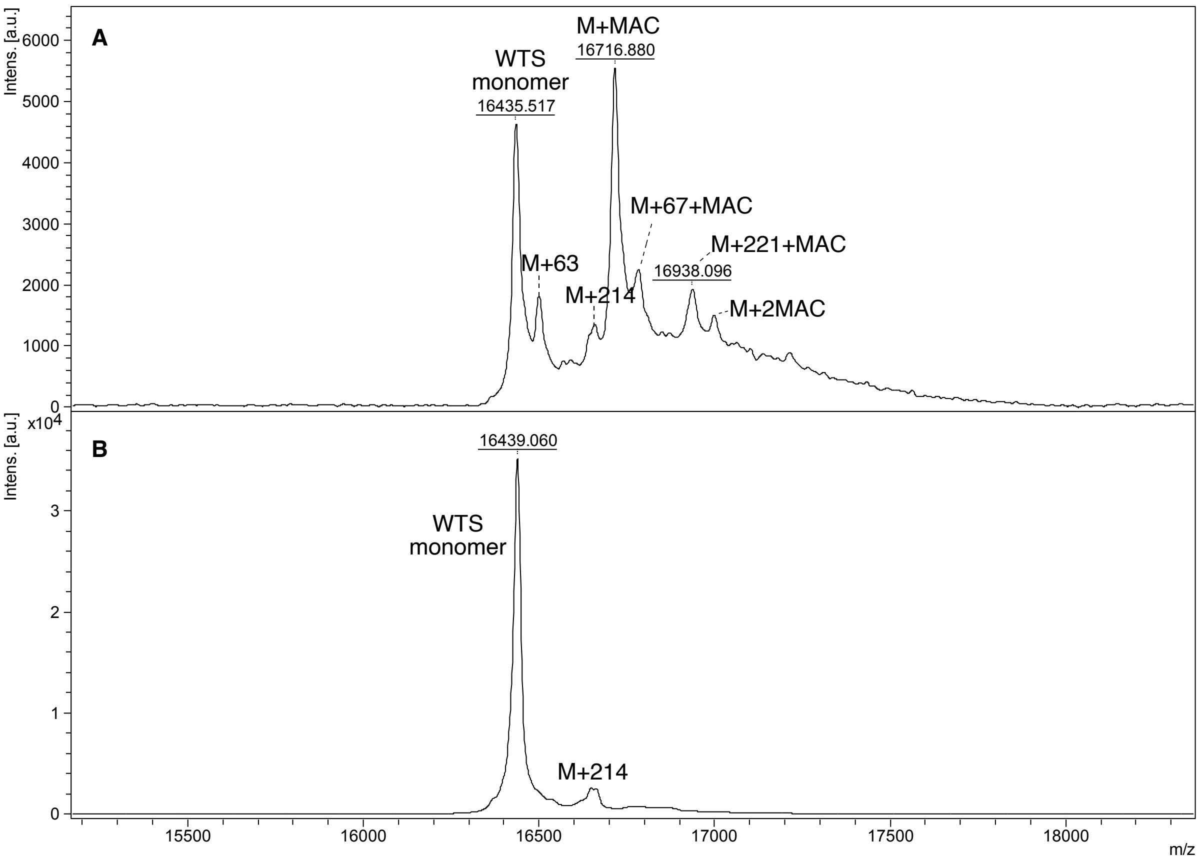

Figure S18. MALDI analysis of (A) MAC conjugate **60** after incubation with **22** for 16 h; (B) nonmodified WTS **23**. M+67: M+MAC – hydration/retro-Aldol MS fragment (Figure S20).

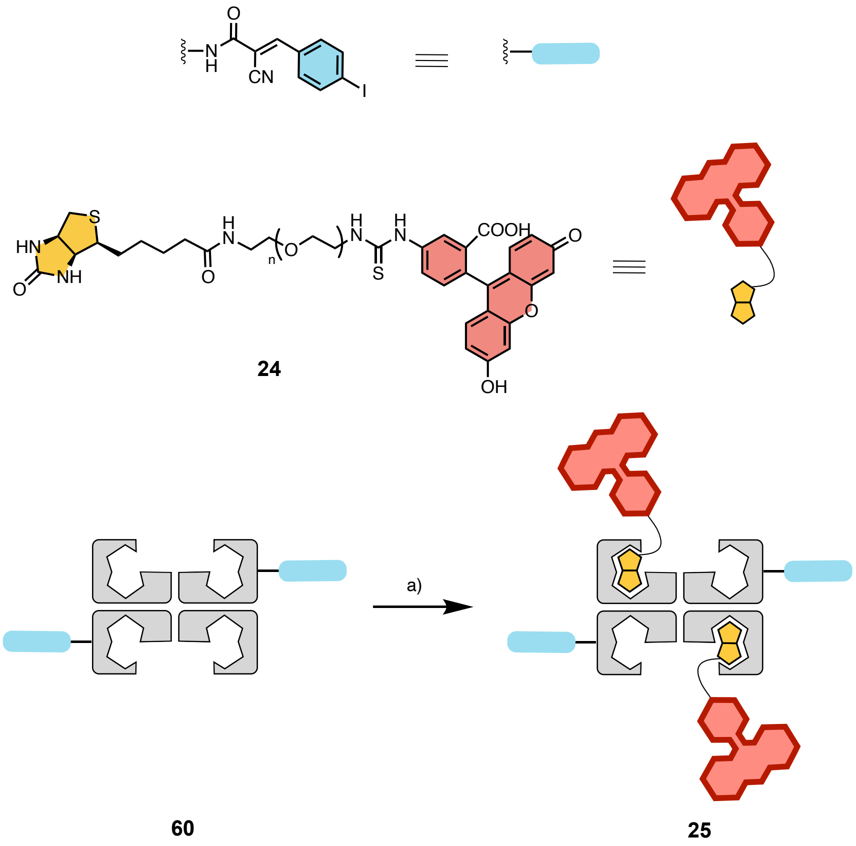

Scheme S7. (a) **24**, PBS, rt, 10 min.

Complex 25. To a solution of **60** (10.6 µM in PBS), 2 eq. of the solution of fluorescein-PEG-biotin **24** (n ≈ 8, 2 mM in H_2O) was added. The mixture was shaken (1000 rpm) for 10 min at rt, filtered through a centrifugal cutoff filter (Amicon® Ultra 0.5 mL, 30 kDa) and washed 4 times with PBS buffer (0.4 mL each), until the filtrate became colorless. The mixture was further concentrated to give 190 µM stock solutions in PBS buffer (pH 7.4).

Complex 26. To a solution of **23** (8 µM in PBS), 2 eq. of the solution of **24** (2 mM in H_2O) was added. The mixture was shaken (1000 rpm) for 10 min at rt. Excess of **24** was removed using centrifugal cutoff filter (Amicon® Ultra 0.5 mL, 30 kDa) and washed 4 times with PBS buffer (0.4 mL each), until the filtrate became colorless. The mixture was further concentrated to give 160 µM stock solutions in PBS buffer (pH 7.4).

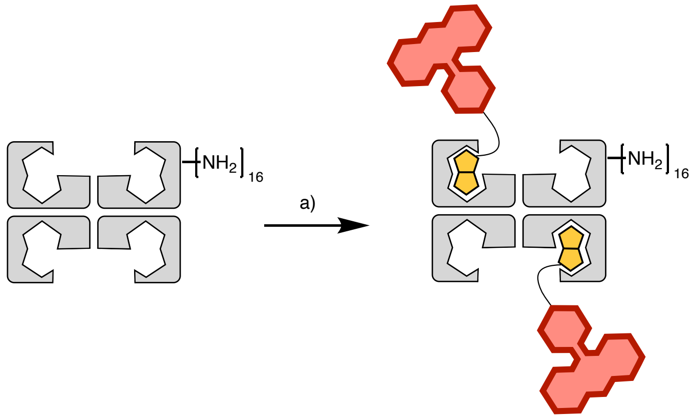

23 **26**

Scheme S8. (a) **24**, PBS, rt, 10 min.

5.2. Cellular Uptake of Streptavidin Complexes

Following the general procedure in reference [S1]. HeLa Kyoto cells were seeded at 8 $\times 10^4$ cells/mL in FluoroBrite DMEM + 10% FBS on µ-Plate 96-well Black ibiTreat sterile (150 µL per well) and incubated at 37 °C with 5% CO_2 for 24 h. Then, the medium was removed, and cells were washed with PBS (3 × 3 mL/well), and then with fresh FluoroBrite DMEM medium (4 × 150 µL/well) using a plate washer (Biotek EL406®), keeping a final volume of 135 µL/well. Different concentrations of the protein complex **25** and **26** (10× final concentration in PBS), a solution of Hoechst 33342 (100 µg/mL) and PI (10 µg/mL) in PBS were prepared freshly in a 96-well V-bottom plate before adding to the cells. The protein complex solution **25** and **26** from the V-bottom plate was added (15 µL/well) to the cells in a final volume of 150 µL/well. A final concentration of protein complex **25** and **26** were 10 µM except for the control wells, where only PBS was added (15 µL/well). Cells treated with protein complexes **25** and **26** were incubated for 4 and 8 h, at 37 °C with 5% CO_2. After that, the cells were washed with PBS and DMEM using the plate washer, and the solution of Hoechst 33342 and PI from the V-bottom plate was added (15 µL/well) to the cells in a final volume of 150 µL/well. After 20 min of incubation at 37 °C with 5% CO_2, the cells were washed and kept in clean FluoroBrite DMEM. For live cell imaging, samples were kept at 37 °C with 5% CO_2

during the microscope imaging. The distribution of fluorophores in cells was analyzed using an IXM-C automated microscope. 9 images per well were acquired using 20× objective lens, and fluorescent images were acquired with three channels, blue for Hoechst 33342 with 377/50 nm excitation filter and 477/60 nm emission filter, green for FITC fluorophore with 475/34 nm excitation filter and 536/40 nm emission filter, and red for PI 531/40 nm excitation filter and 593/40 nm emission filter.

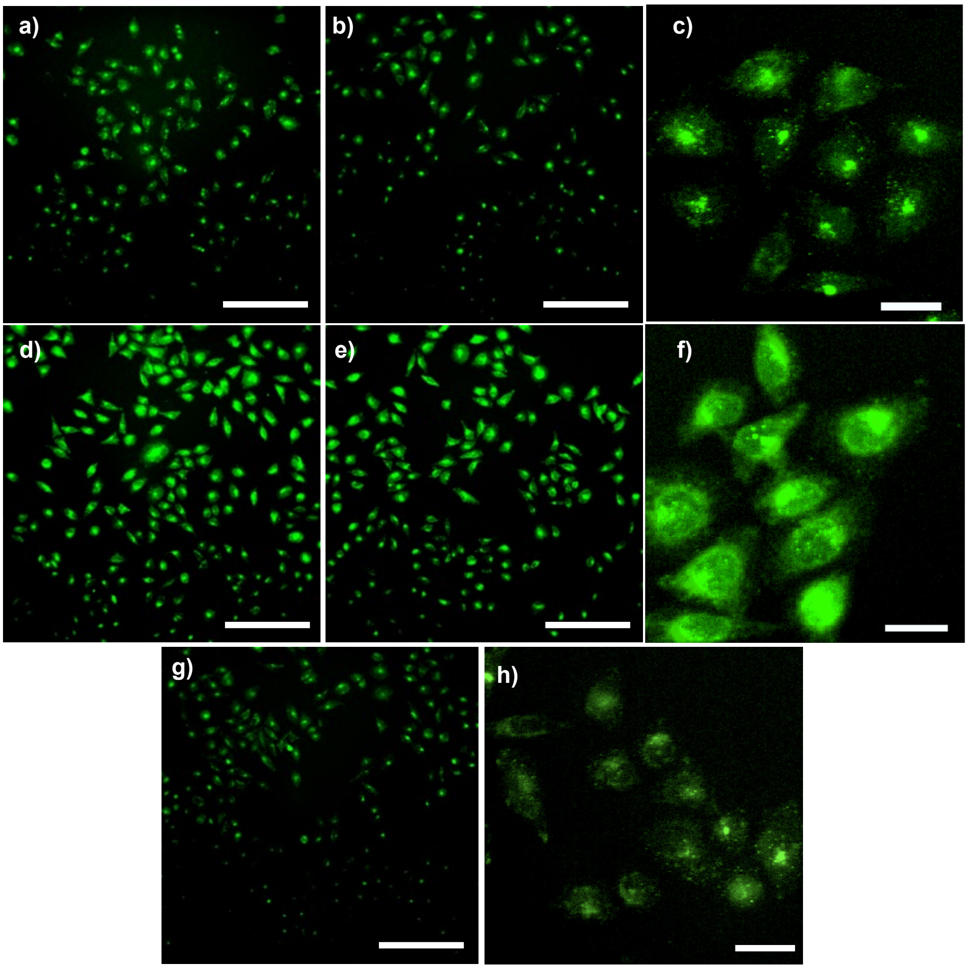

Figure S19. CLSM images of HeLa Kyoto cells after incubation with 10 μM of nonmodified complex **26** for 4 h (a) and 8 h (b, c), or 10 μM of MA labeled complex **25** for 4 h (d) and 8 h (e, f) at 37 °C, negative control with cells treated with only PBS (g, h). Scale bars = 200 μm (a, b, d, e, g) or 25 μm (c, f, h).

6. Stability of Michael Acceptors in Aqueous Media

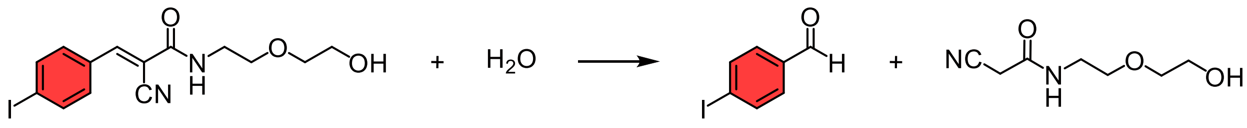

10 **41** **37**

Scheme S9. Potential hydrolysis of compound **10** in aqueous medium.

The compound **10** was chosen as a representative Michael acceptor scaffold. The stability was monitored by UV spectroscopy following the absorbance peak at 320 nm in 2 different buffers, *i.e.* PBS (pH = 7.4) and AMPSO (10 mM AMPSO, 100 mM NaCl, pH = 8.7). The absorbance of compound **10** (5 µM, in indicated buffer) was measured for 10 hours with an interval of 30 minutes. Additional measurement was performed after 72 h in buffer. The whole set of data was normalized and summarized in Figure S20.

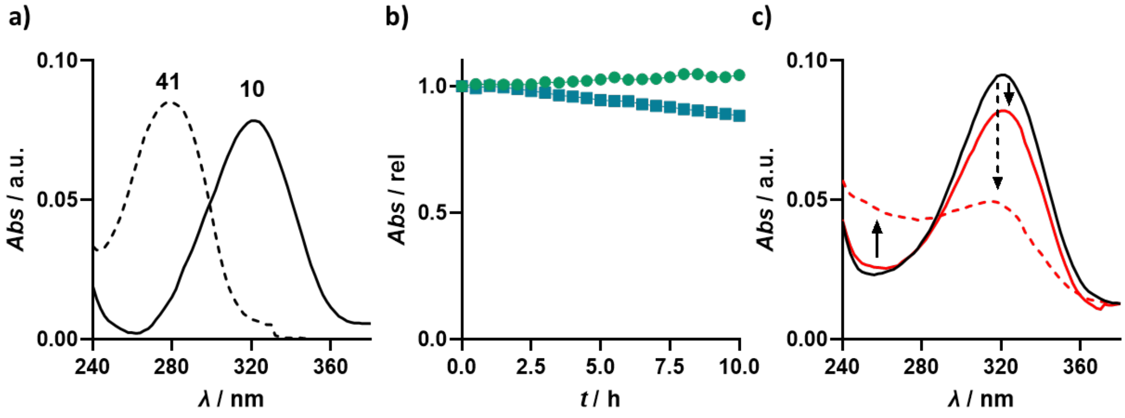

Figure S20. Stability of **10** in aqueous medium: a) UV spectra of compounds **41** (dashed) and **10** (solid) in PBS; b) normalized absorbance of **10** at 320 nm in 2 different buffers (green line, PBS, pH = 7.4; blue line, AMPSO, pH = 8.7); c) spectra of **10** at 0 h (black line) and 72 h in PBS (red, solid line) and in AMPSO (red, dashed line).

7. Supporting References

[S1] Y. Cheng, A.-T. Pham, T. Kato, B. Lim, D. Moreau, J. López-Andarias, L. Zong, N. Sakai, S. Matile, *Chem. Sci.* **2021**, *12*, 626–631.

[S2] Y. Zhong, Y. Xu, E. V. Anslyn, *Eur. J. Org. Chem.* **2013**, 5017–5021.

[S3] A. G. Neo, J. Díaz, S. Marcaccinib, C. F. Marcos, *Org. Biomol. Chem.* **2012**, *10*, 3406-3416 .

[S4] Y. Zi , M. Lange, P. Schüler, S. Krieck, M. Westerhausen, I. Vilotijevic, *Synlett.* **2020**, *31*, 575-580.

[S5] P. H. Mason, N. D. Emslie, *Tetrahedron* **1994**, *50*, 12001–2008.

[S6] A.Yasin, V.S. Nair, S. A. Aravindh, S. M. Sarkar, M. Hatamimoslehabadi, S. Mitra, M. H. A. Rahim, J. La, I. S. Roqan, M. M. Yusoff, C. S. Yelleswarapu, R. Jose*, Mater. Chem. C.* **2020**, *8*, 8546–8559.

[S7] R. Herges, A. Dikmans, U. Jana, F. Köhler, P. G. Jones, I. Dix, T. Fricke, B. König, *Eur. J. Org. Chem*. **2002**, 3004–3014.

[S8] L. Zong, E. Bartolami, D. Abegg, A. Adibekian, N. Sakai, S. Matile, *ACS Cent. Sci.* **2017**, *3*, 449–453.

[S9] Y. Cheng, L. Zong, J. López-Andarias, E. Bartolami, Y. Okamoto, T. R. Ward, N. Sakai, S. Matile, *Angew. Chem. Int. Ed*. **2019**, *58*, 9522–9526.

[S10] T. Kato, B. Lim, Y. Cheng, A.-T. Pham, J. Maynard, D. Moreau, A. I. Poblador-Bahamonde, N. Sakai, S. Matile, *JACS Au* **2022**, *2*, 839–852.

[S11] B. Lim, T. Kato, C. Besnard, A. I. Poblador Bahamonde, N. Sakai, S. Matile, *JACS Au* **2022**, *2*, 1105-1115.

[S12] Q. Laurent, R. Martinent, D. Moreau, N. Winssinger, N. Sakai, S. Matile, *Angew. Chem. Int. Ed.* **2021**, *60*, 19102–19106.

[S13] J. López-Andarias, J. Saarbach, D. Moreau, Y. Cheng, E. Derivery, Q. Laurent, M. González-Gaitán, N. Winssinger, N. Sakai, S. Matile, *J. Am. Chem. Soc.* **2020**, *142*, 4784–4792.

The original data that support the findings of this study are openly available: https://doi.org/10.5281/zenodo.7164389.

8. NMR Spectra

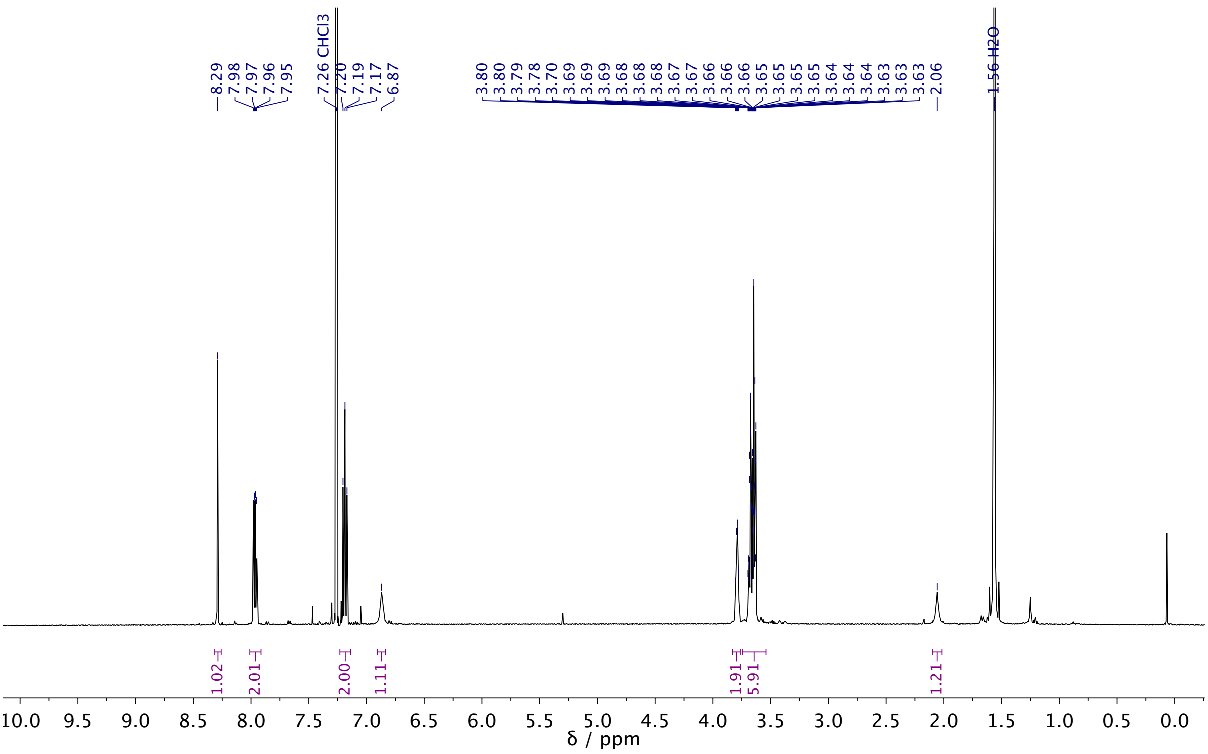

Figure S21. 400 MHz ^1H NMR spectrum of **7** in $CDCl_3$.

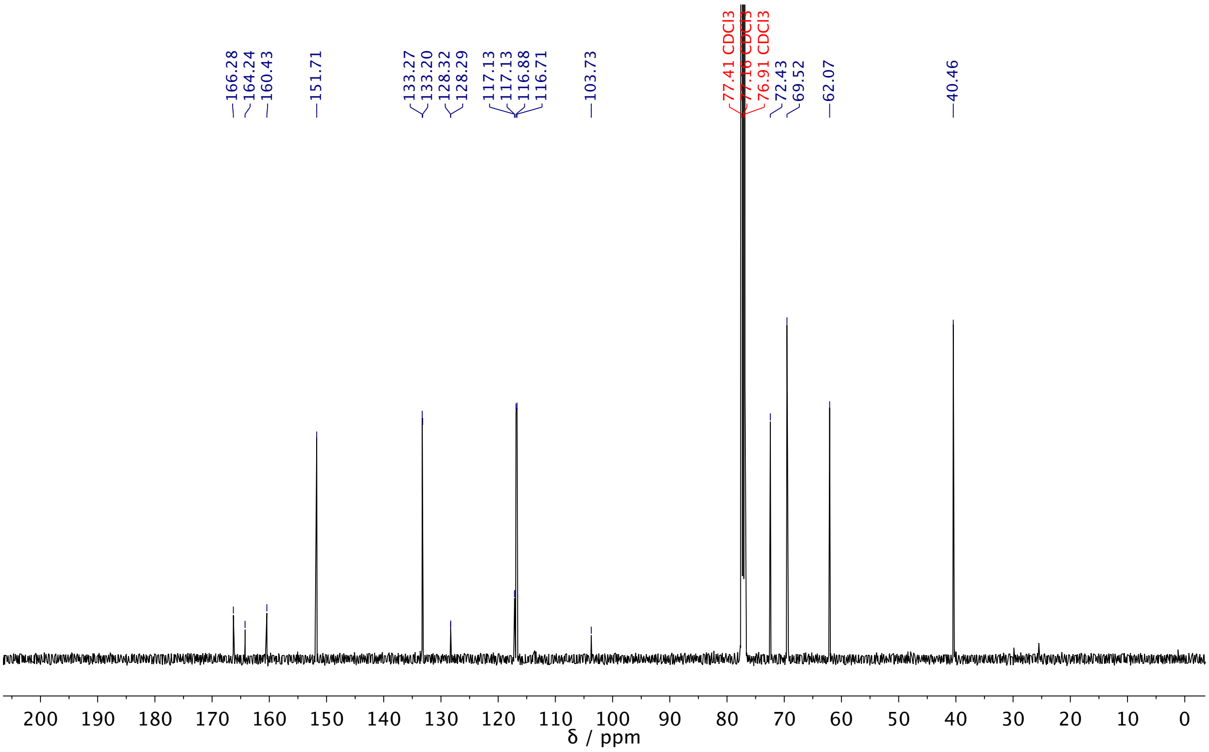

Figure S22. 101 MHz ^{13}C NMR spectrum of **7** in $CDCl_3$.

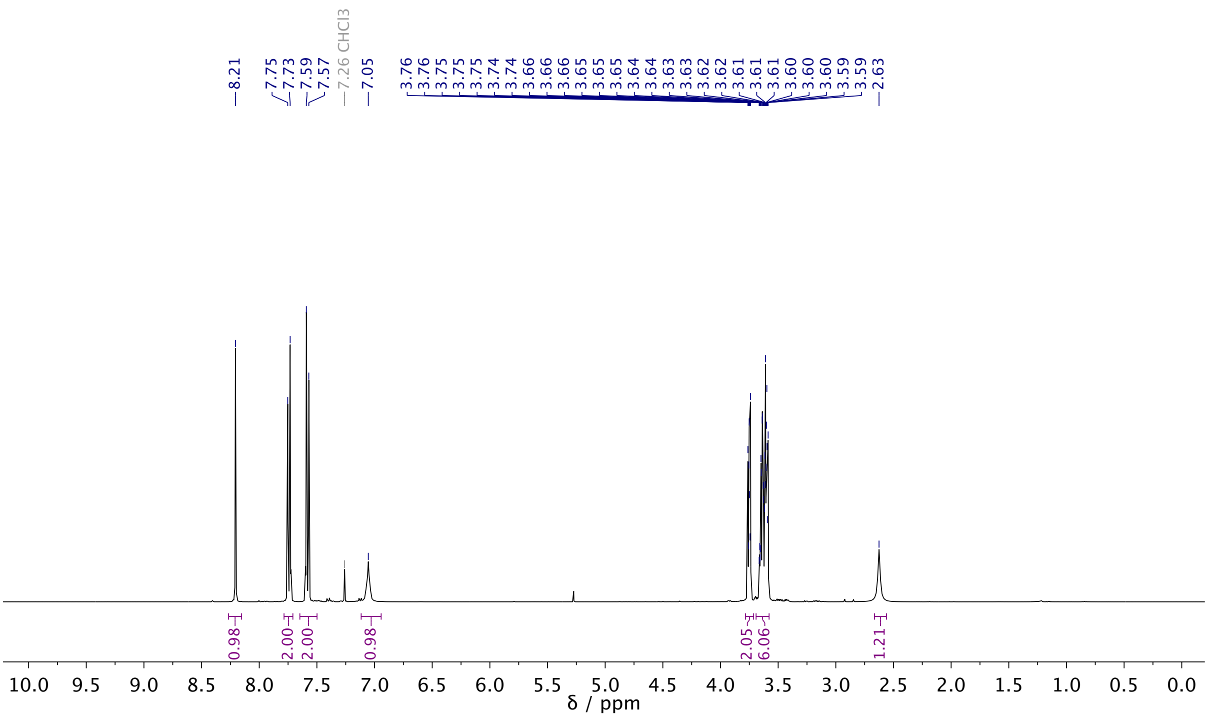

Figure S23. 400 MHz ^1H NMR spectrum of **9** in $CDCl_3$.

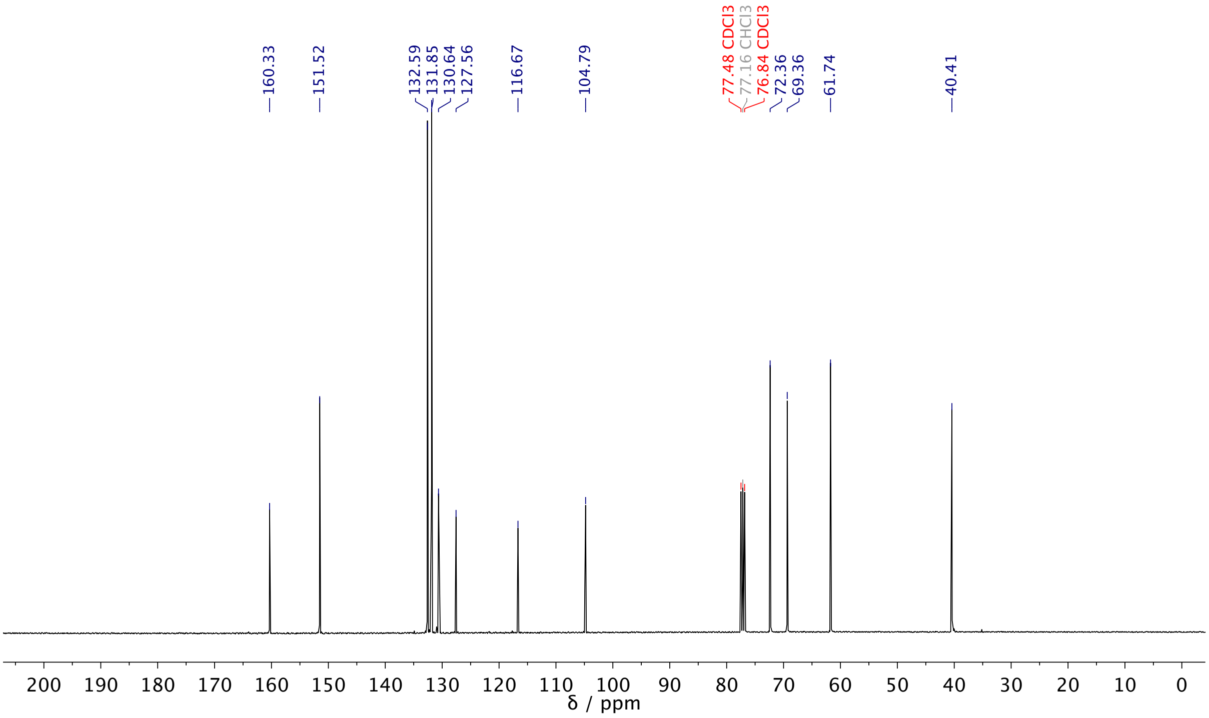

Figure S24. 101 MHz ^{13}C NMR spectrum of **9** in $CDCl_3$.

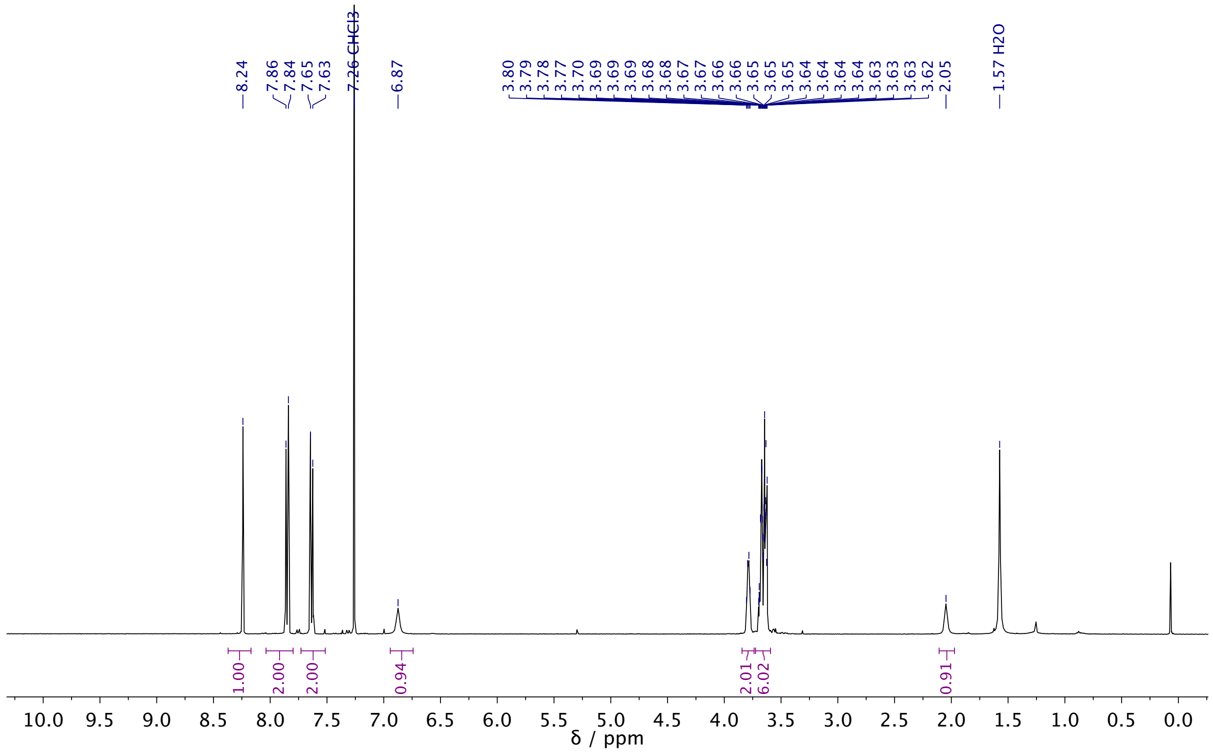

Figure S25. 400 MHz ^{1}H NMR spectrum of **10** in $CDCl_3$.

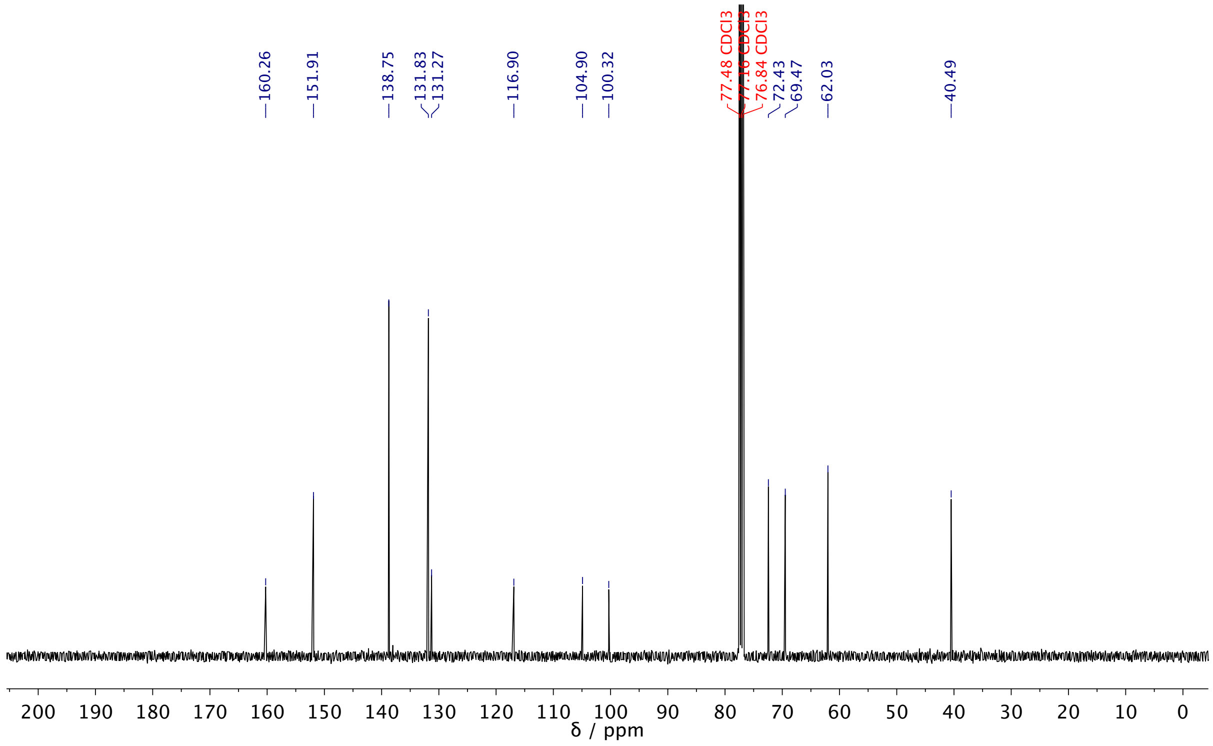

Figure S26. 101 MHz ^{13}C NMR spectrum of **10** in $CDCl_3$.

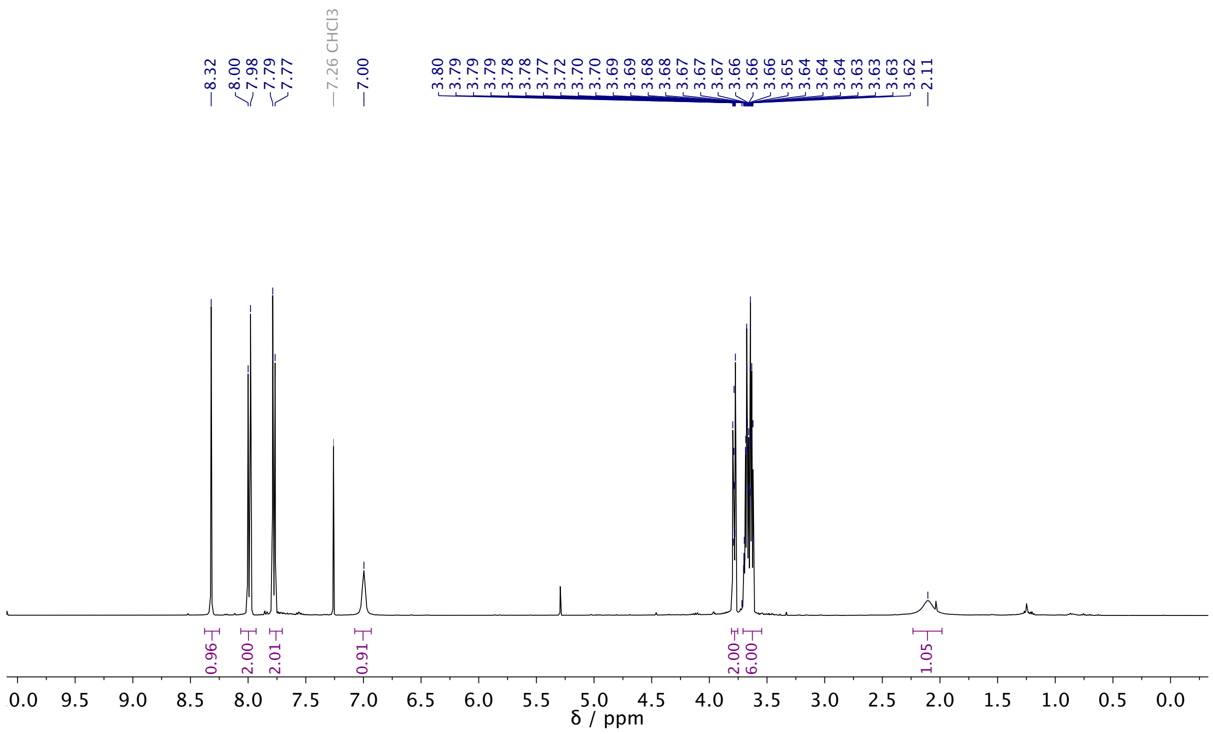

Figure S27. 400 MHz ^{1}H NMR spectrum of **11** in $CDCl_3$.

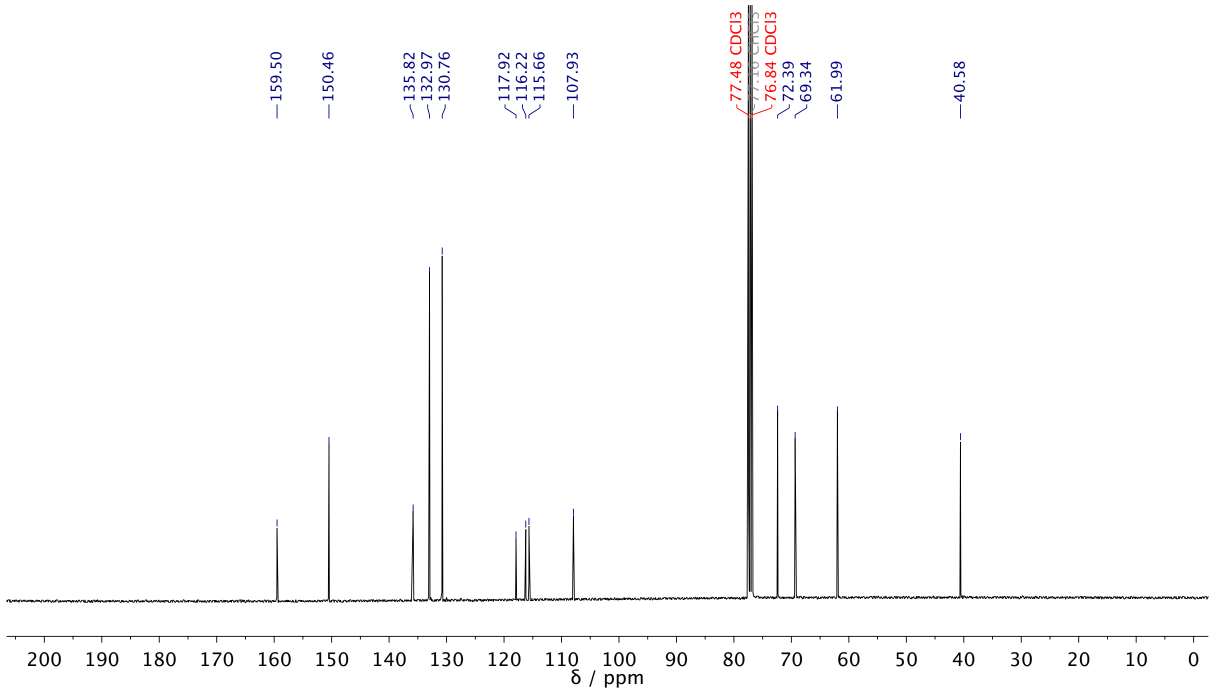

Figure S28. ^{13}C NMR spectrum of **11** in $CDCl_3$.

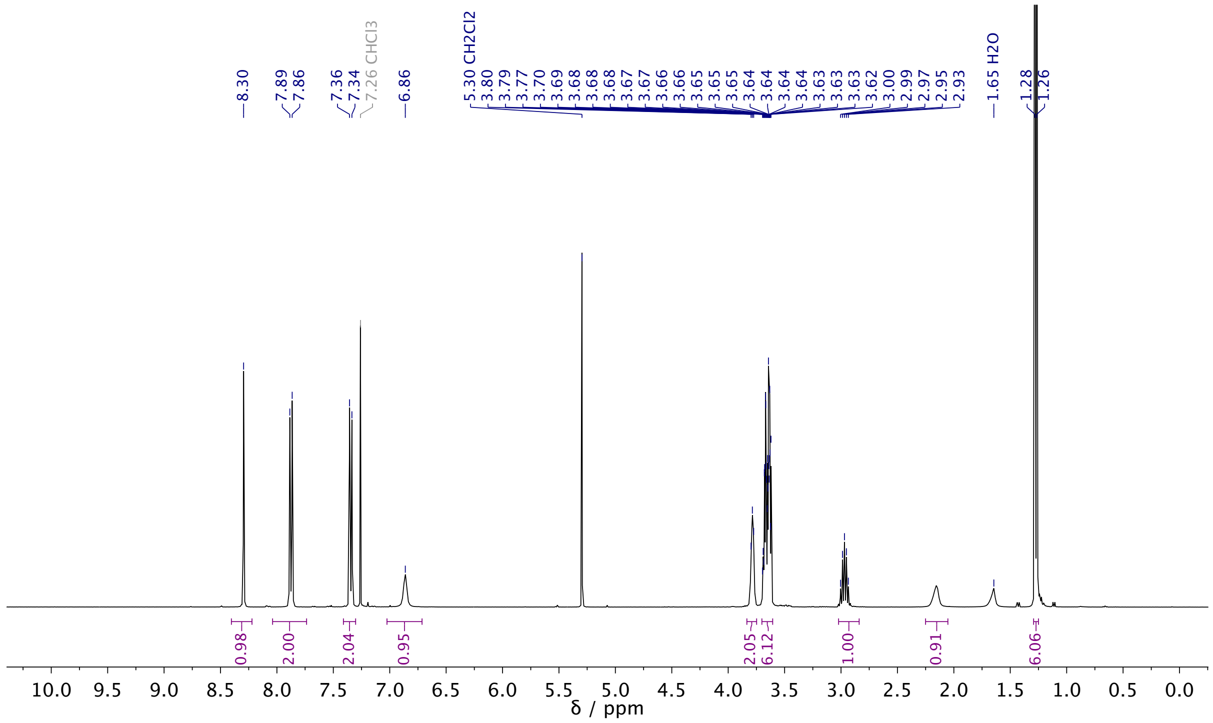

Figure S29. ^{1}H NMR spectrum of **13** in $CDCl_3$.

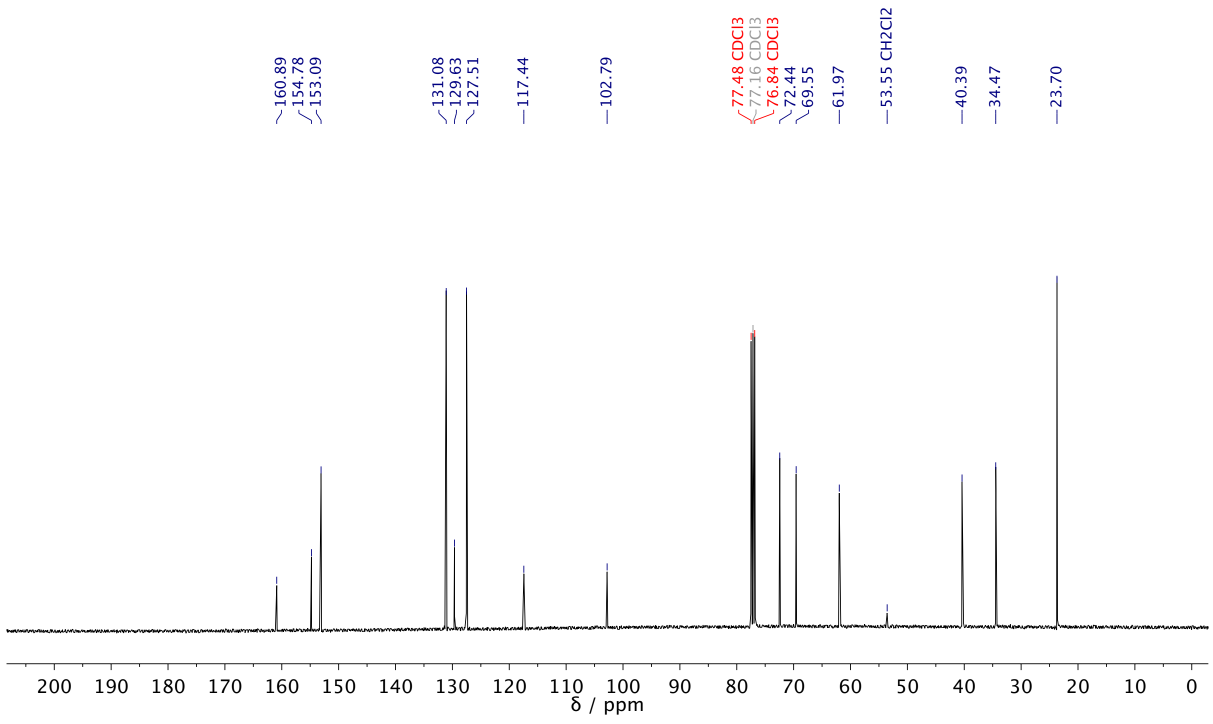

Figure S30. 101 MHz ^{13}C NMR spectrum of **13** in $CDCl_3$.

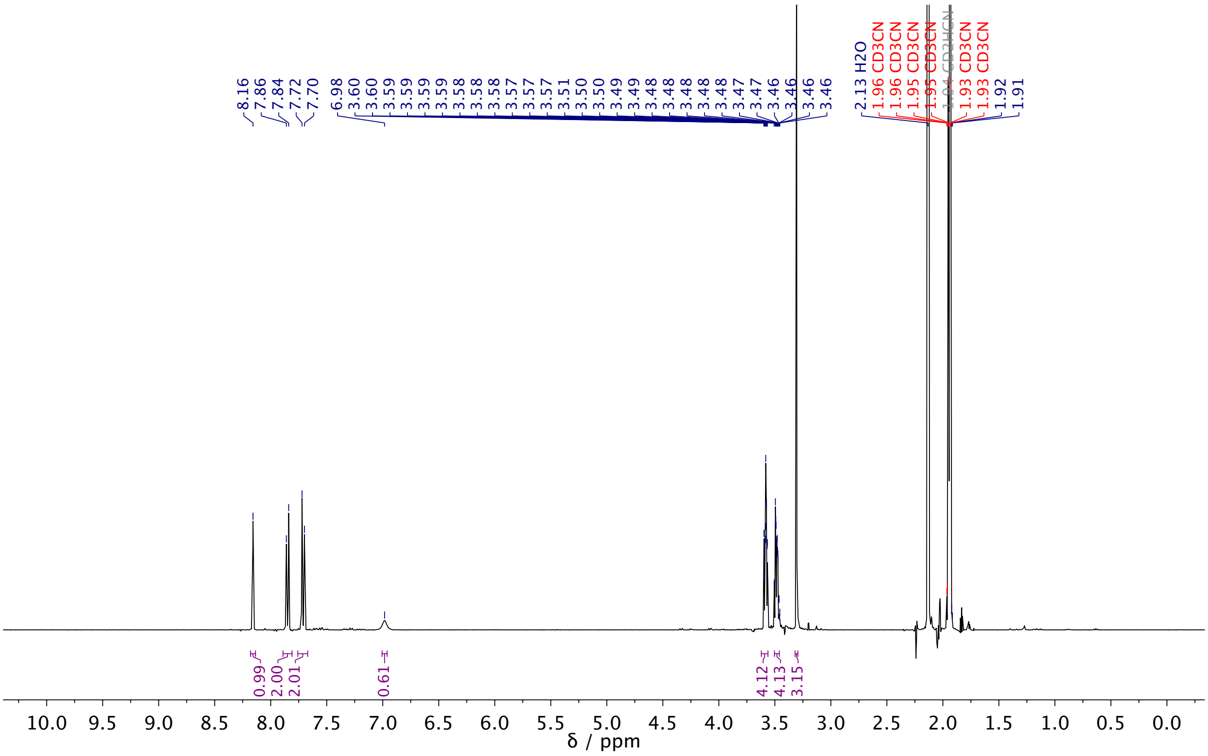

Figure S31. 400 MHz ^{1}H NMR spectrum of **14** in CD_3CN.

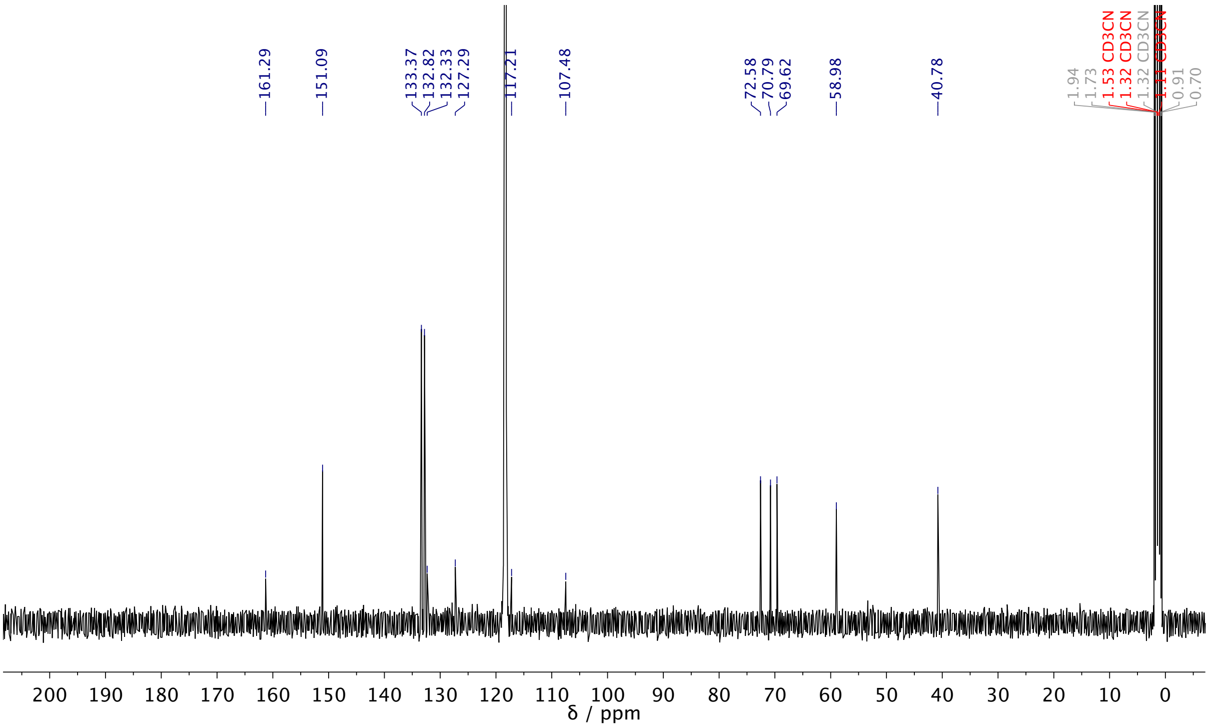

Figure S32. 101 MHz ^{13}C NMR spectrum of **14** in CD_3CN.

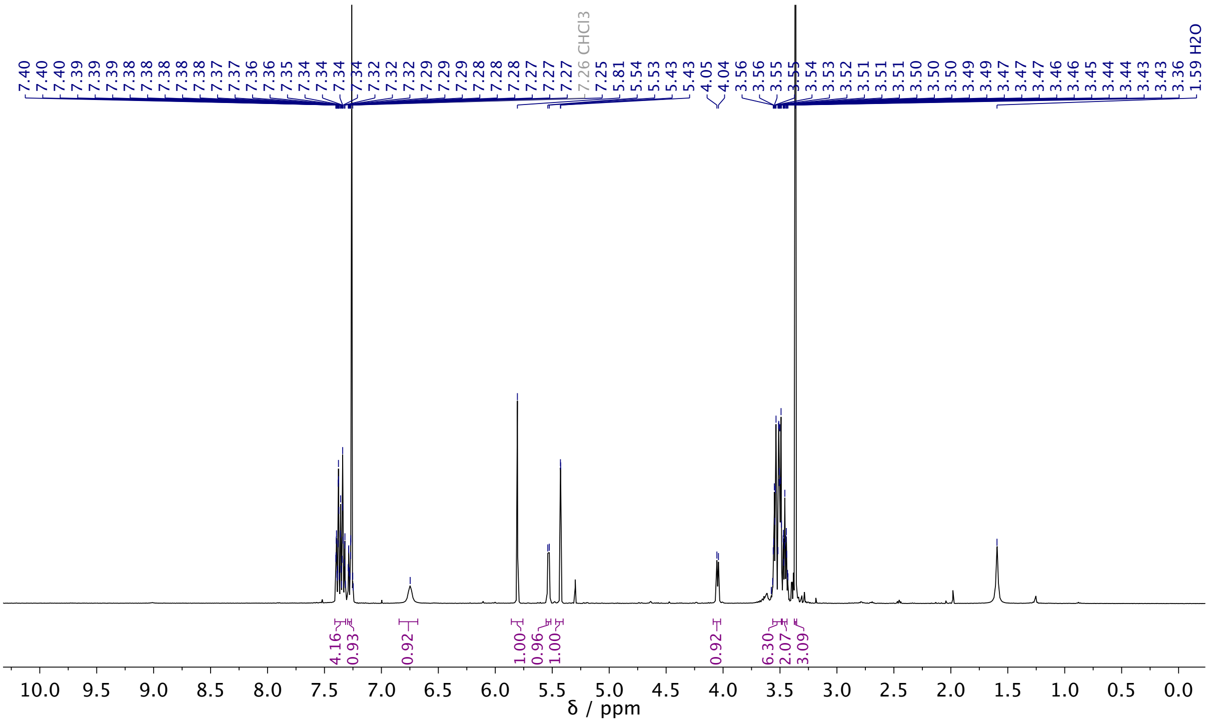

Figure S33. 400 MHz ^{1}H NMR spectrum of **47** in $CDCl_3$.

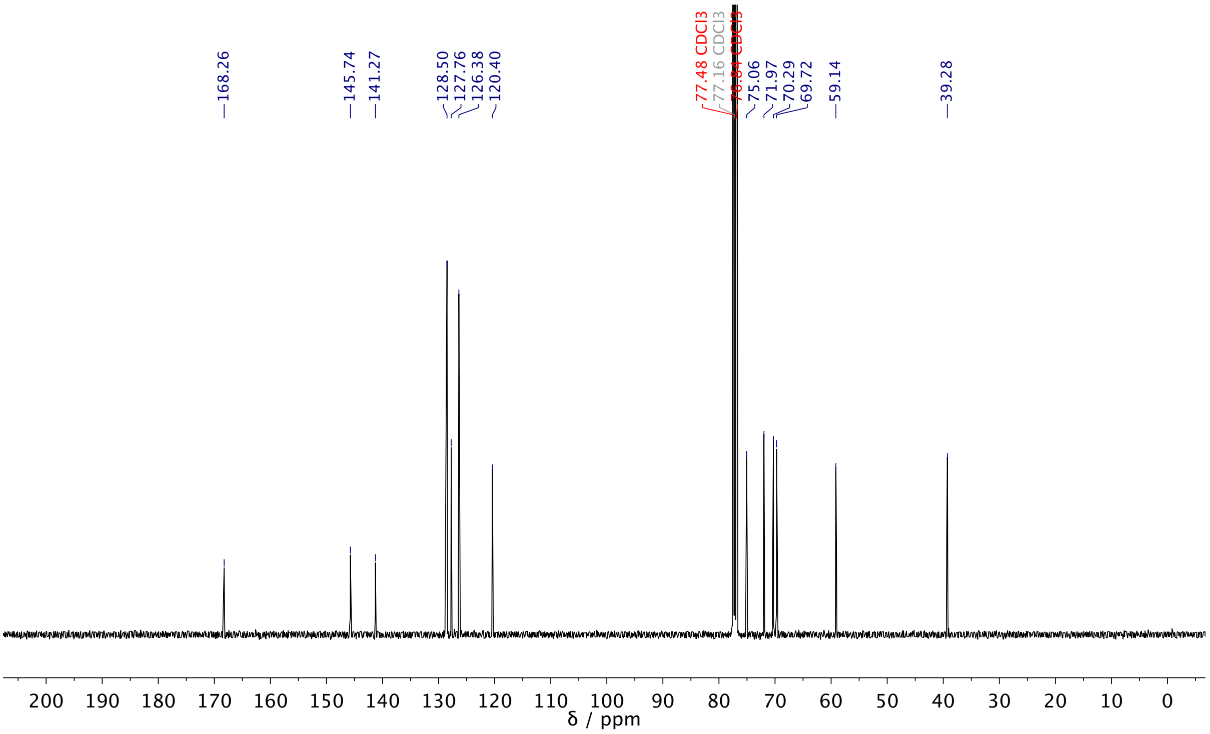

Figure S34. ^{13}C NMR spectrum of **47** in $CDCl_3$.

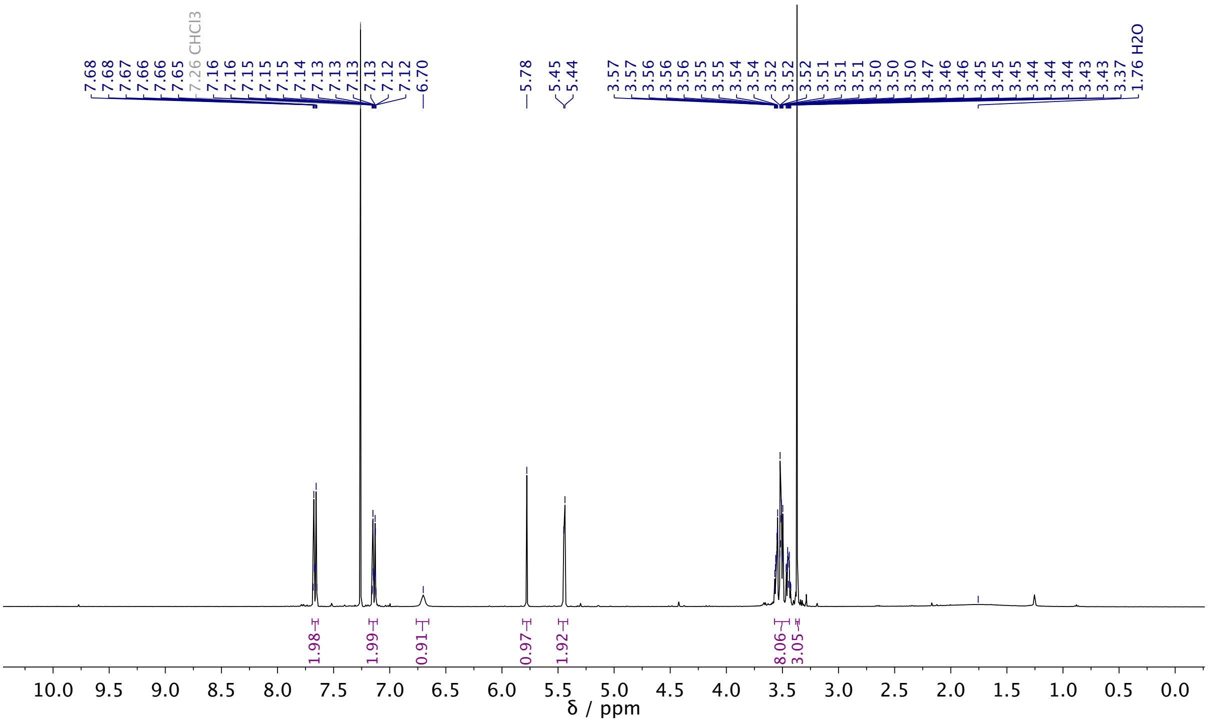

Figure S35. 400 MHz ^{1}H NMR spectrum of **48** in $CDCl_3$.

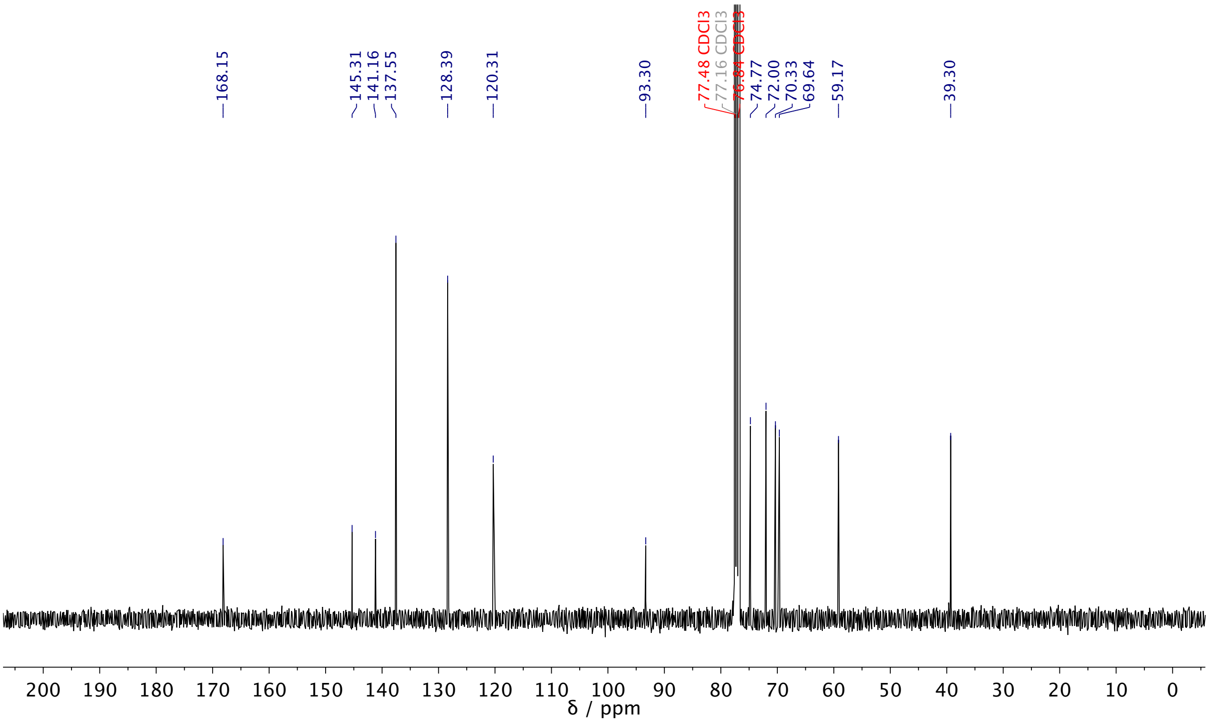

Figure S36. 101 MHz ^{13}C NMR spectrum of **48** in $CDCl_3$.

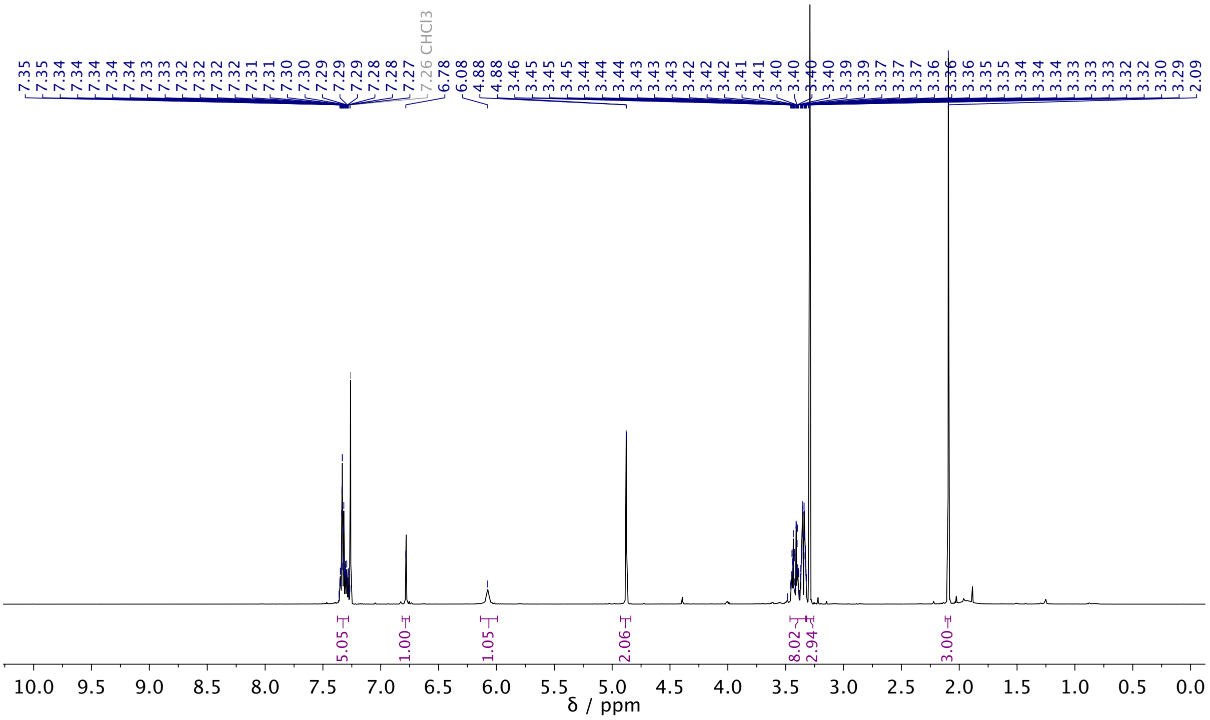

Figure S37. 400 MHz ^{1}H NMR spectrum of **34** in $CDCl_3$.

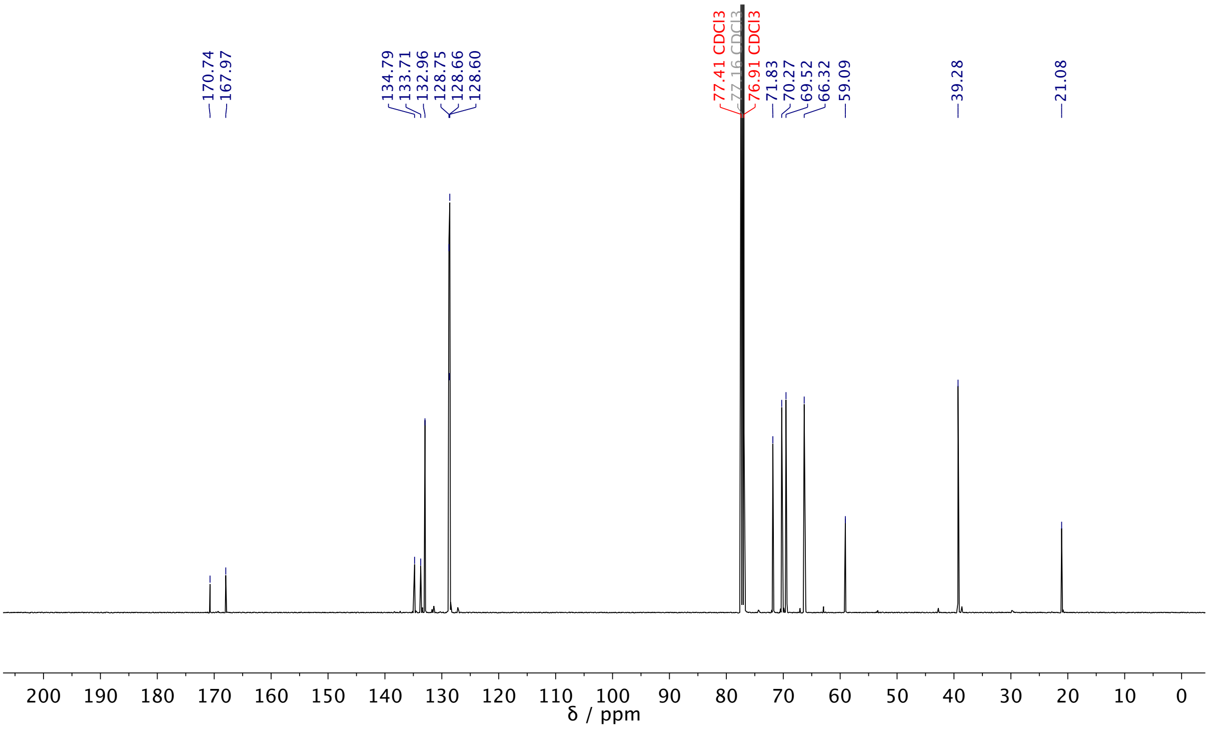

Figure S38. 101 MHz ^{13}C NMR spectrum of **34** in $CDCl_3$.

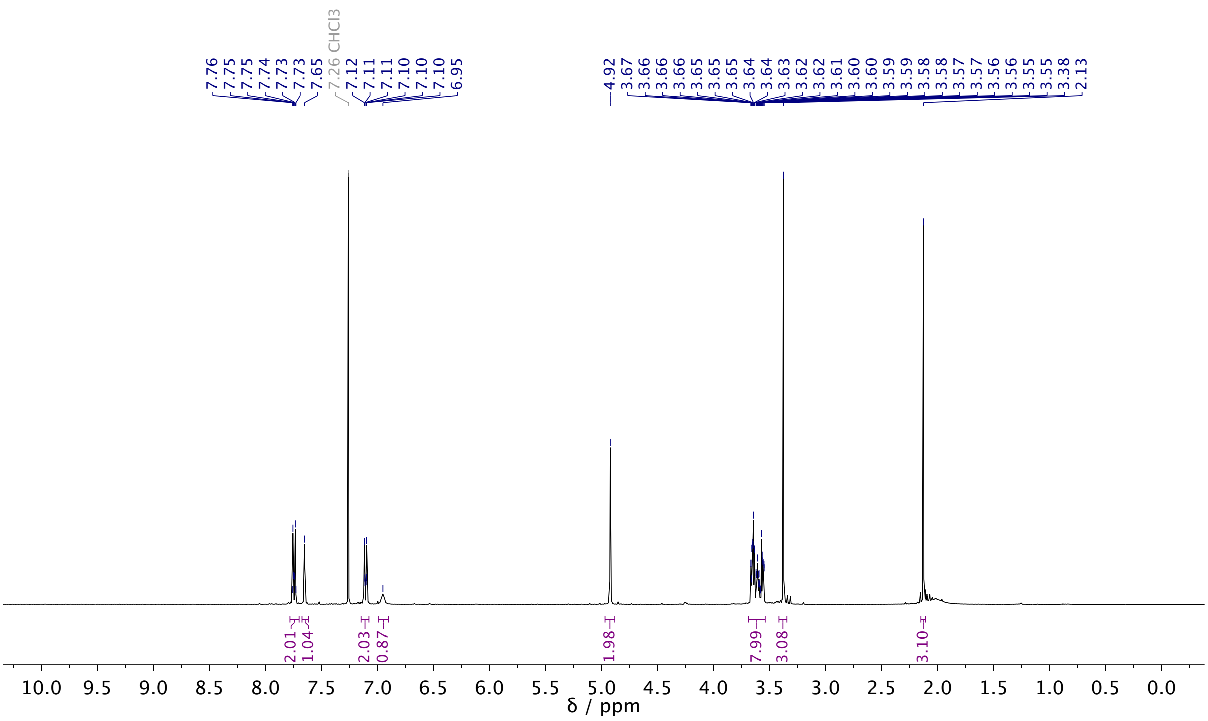

Figure S39. 400 MHz ^{1}H NMR spectrum of **36** in $CDCl_3$.

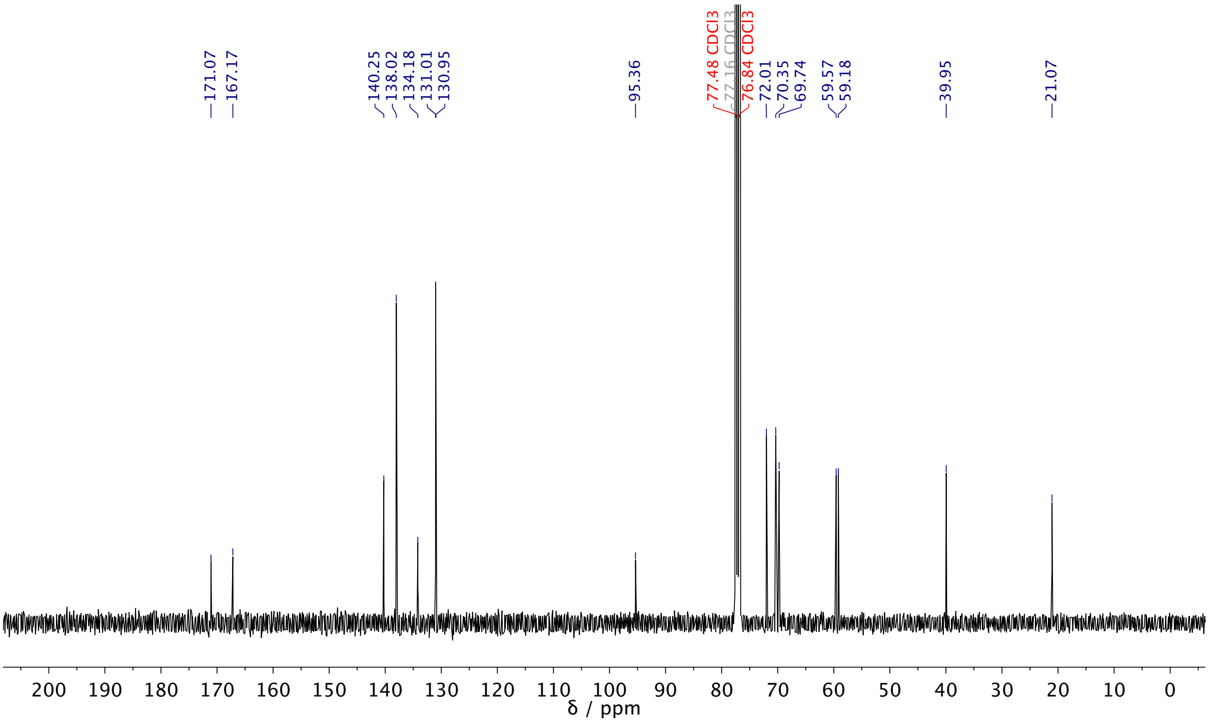

Figure S40. 101 MHz ^{13}C NMR spectrum of **36** in $CDCl_3$.

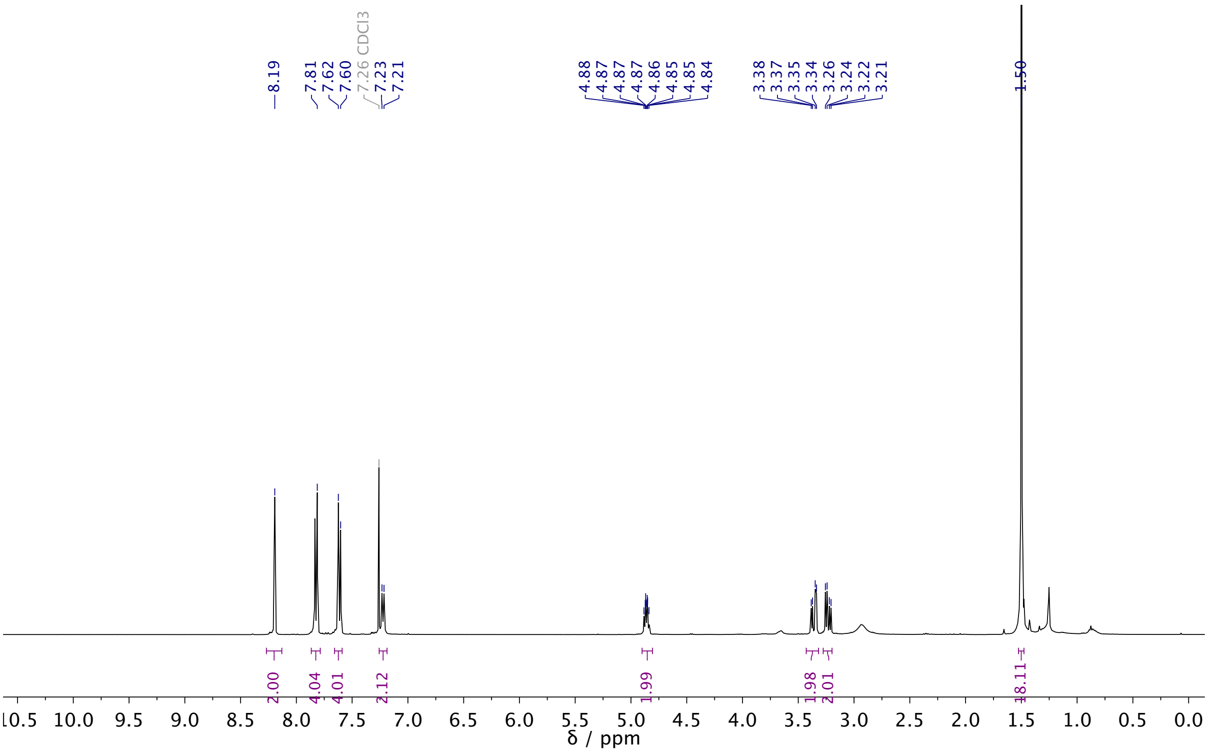

Figure S41. 400 MHz ^{1}H NMR spectrum of **53** in $CDCl_3$.

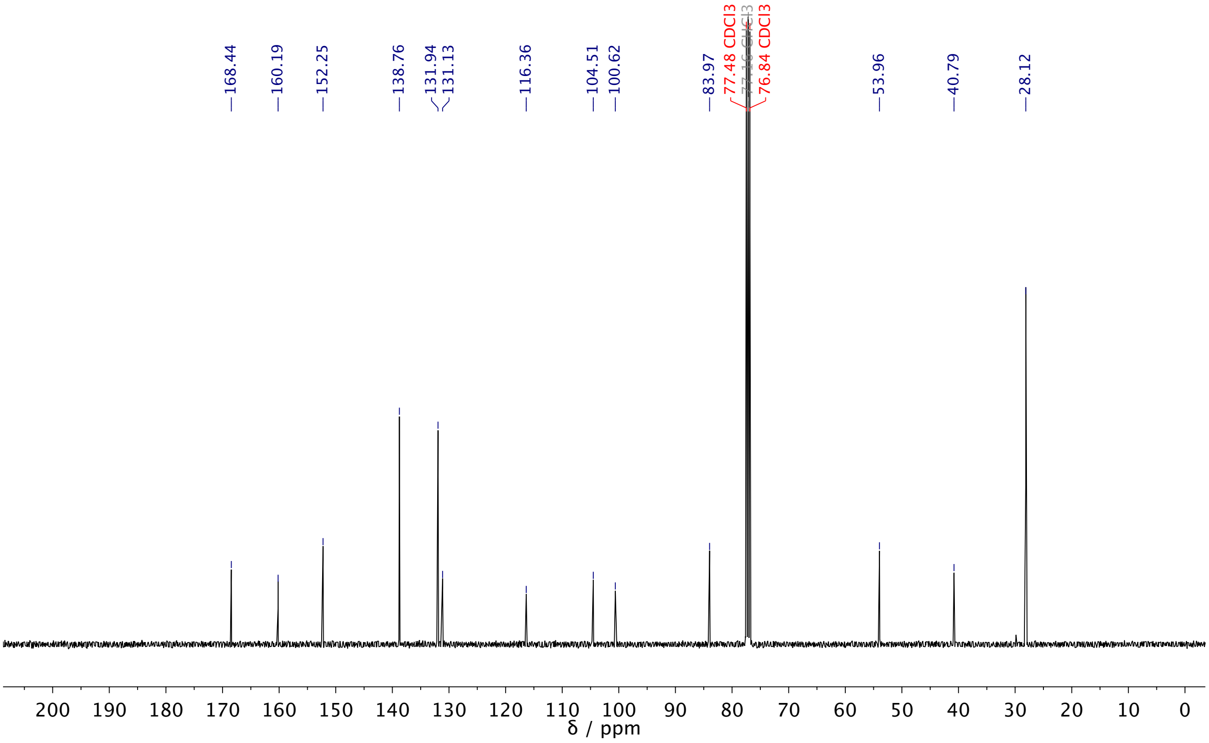

Figure S42. 101 MHz ^{13}C NMR spectrum of **53** in $CDCl_3$.

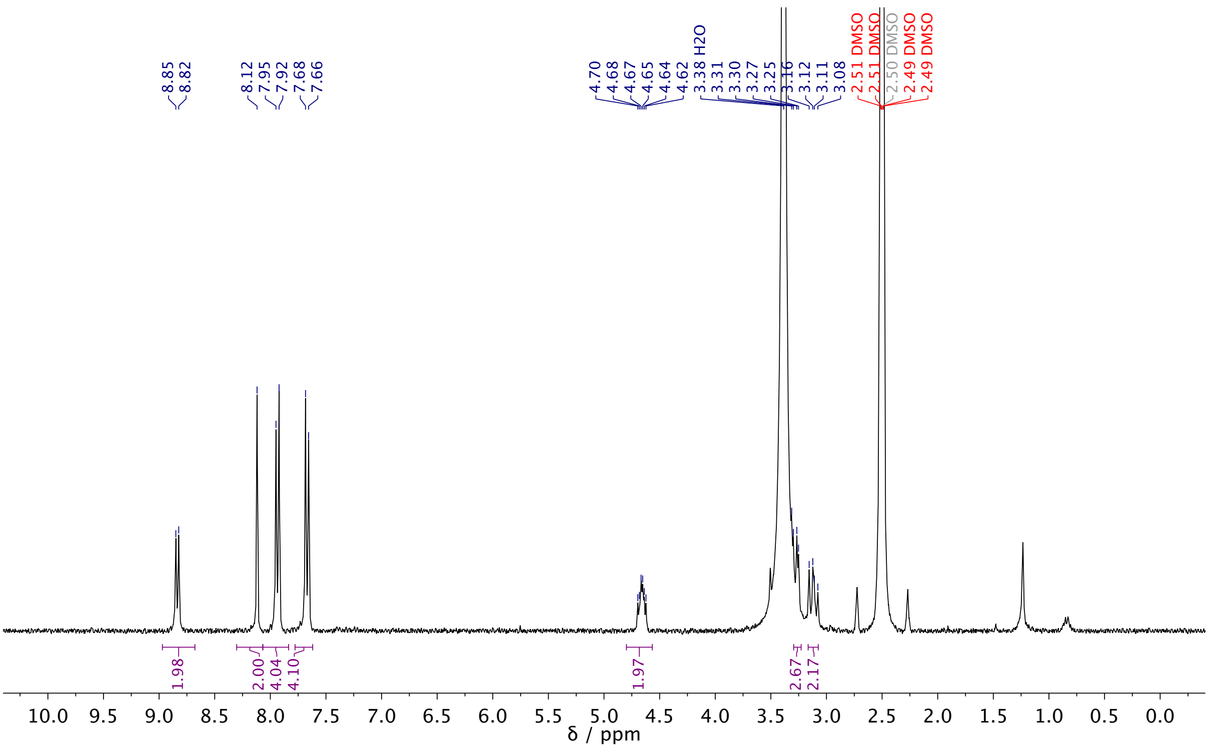

Figure S43. 500 MHz ^{1}H NMR spectrum of **33** in DMSO-d_6.

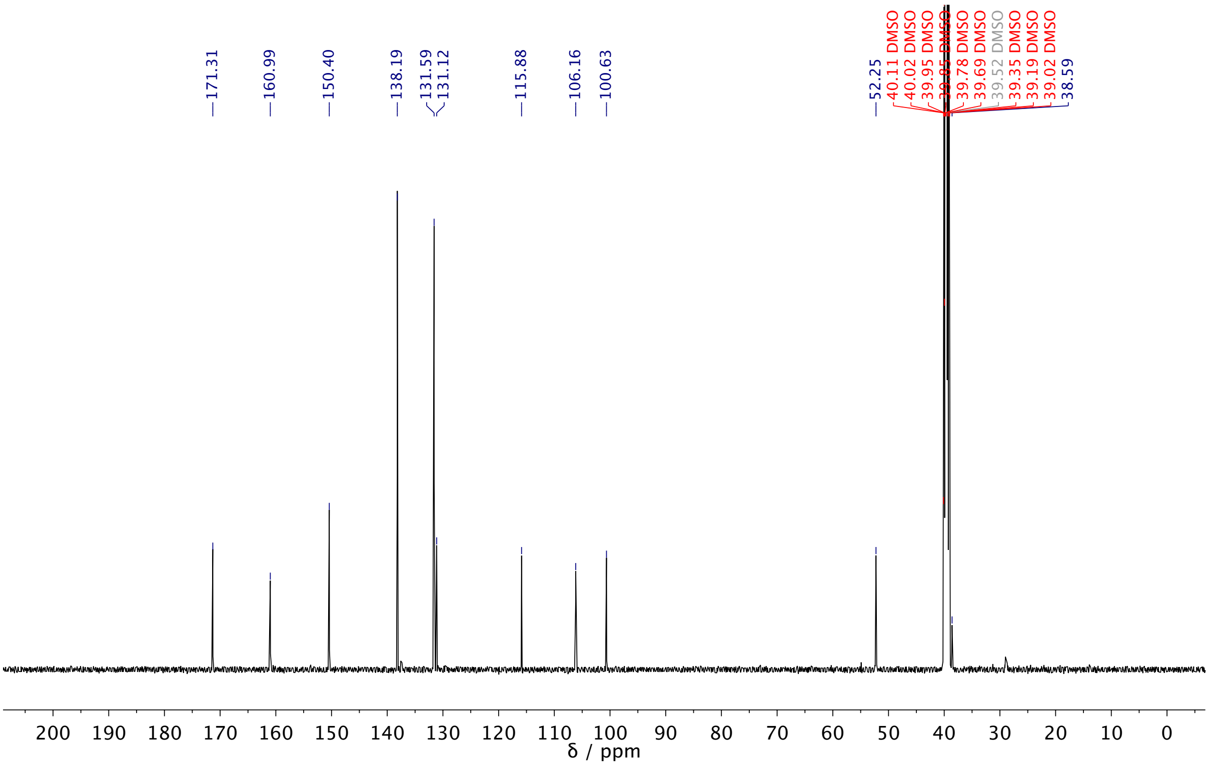

Figure S44. 126 MHz ^{13}C NMR spectrum of **33** in DMSO-d_6.

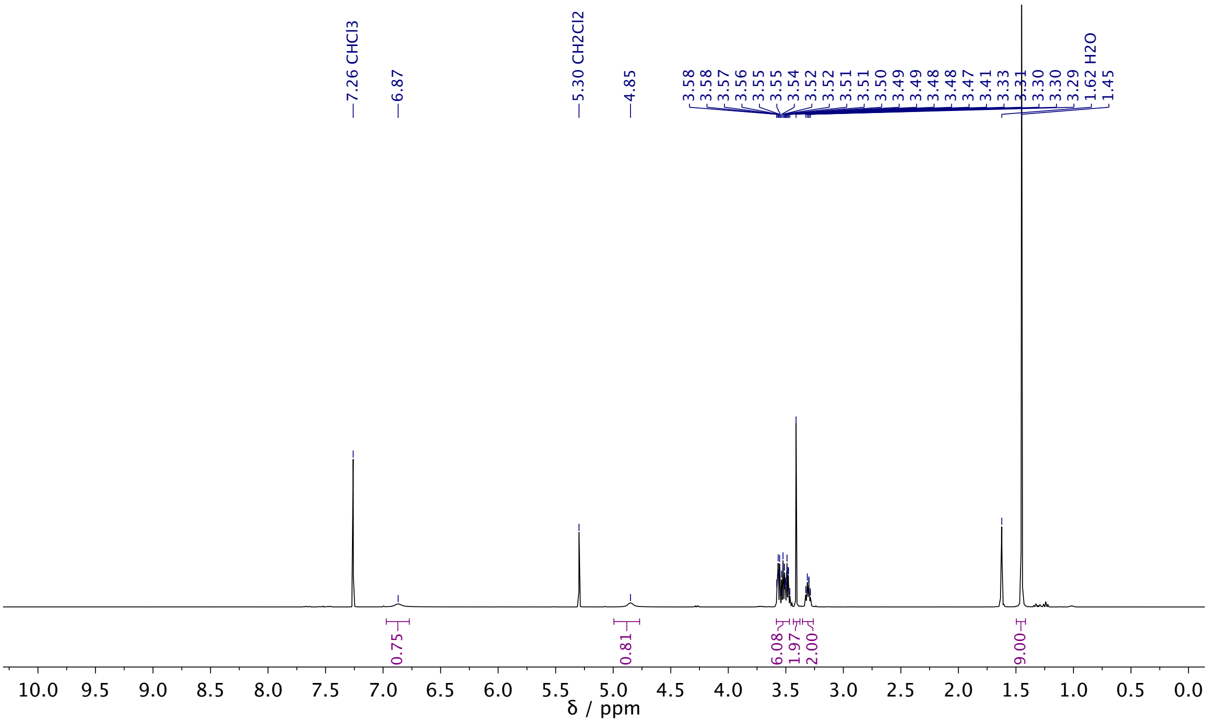

Figure S45. 400 MHz ^{1}H NMR spectrum of **55** in $CDCl_3$.

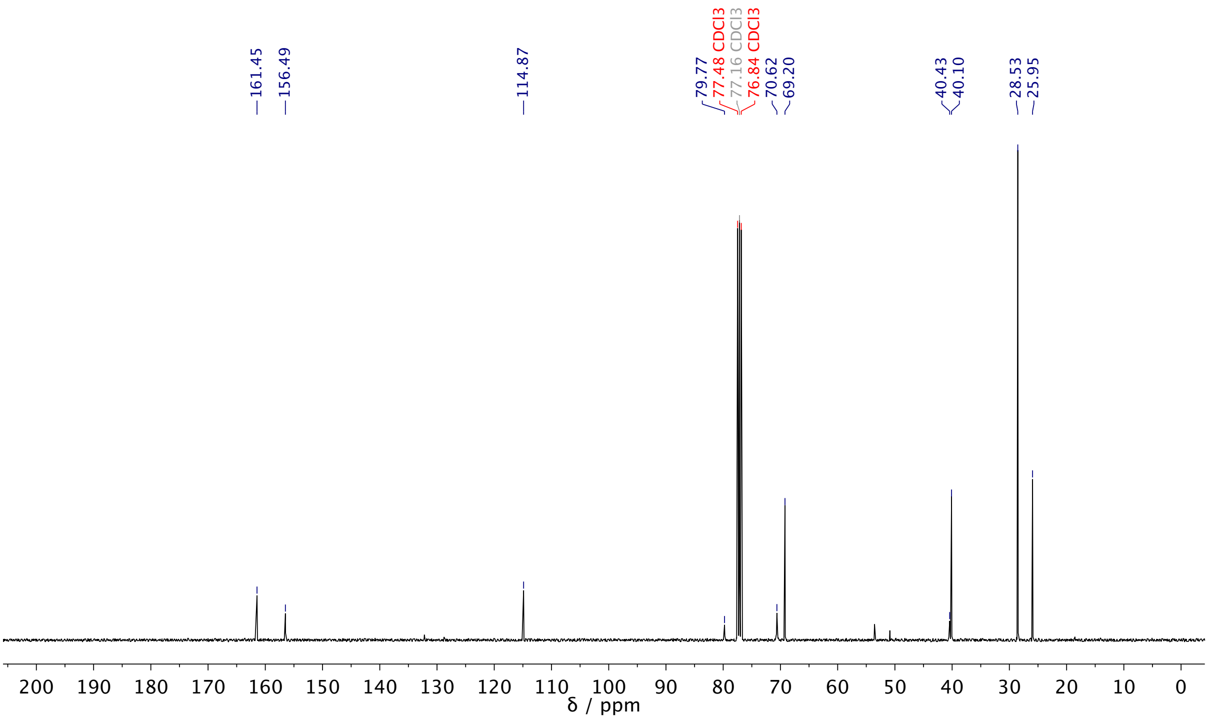

Figure S46. 101 MHz ^{13}C NMR spectrum of **55** in $CDCl_3$.

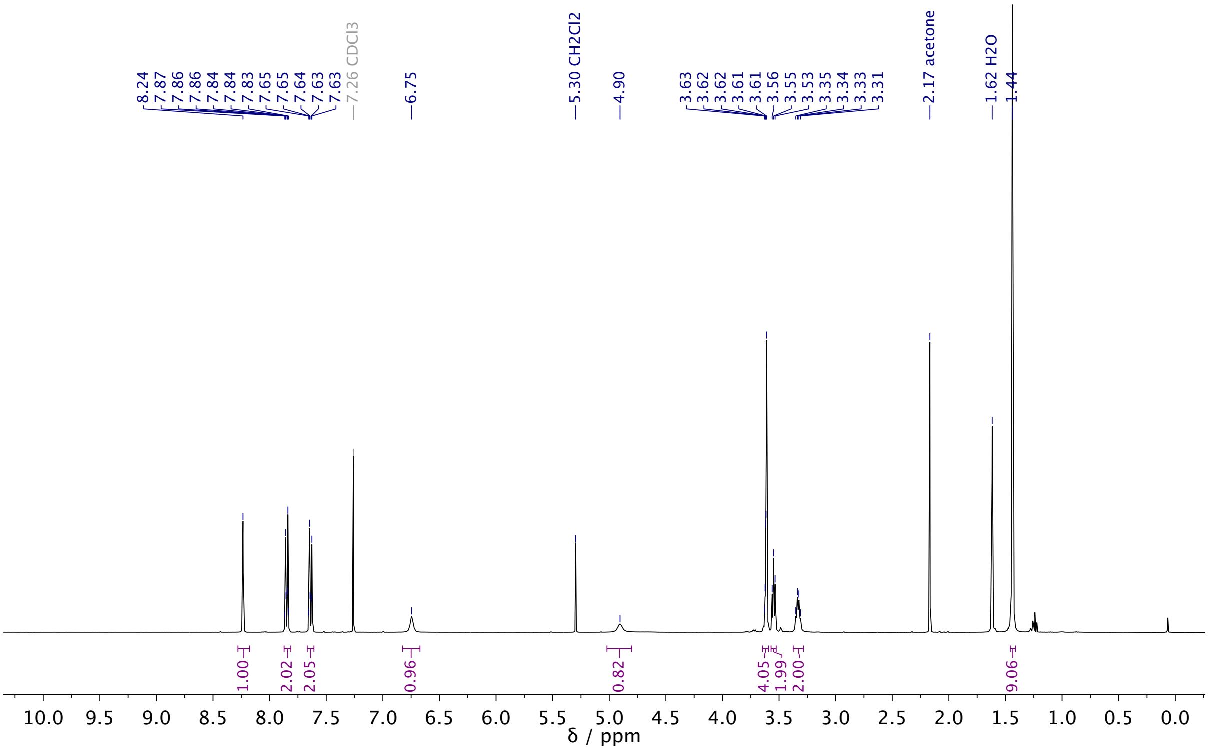

Figure S47. 400 MHz ^{1}H NMR spectrum of **56** in $CDCl_3$.

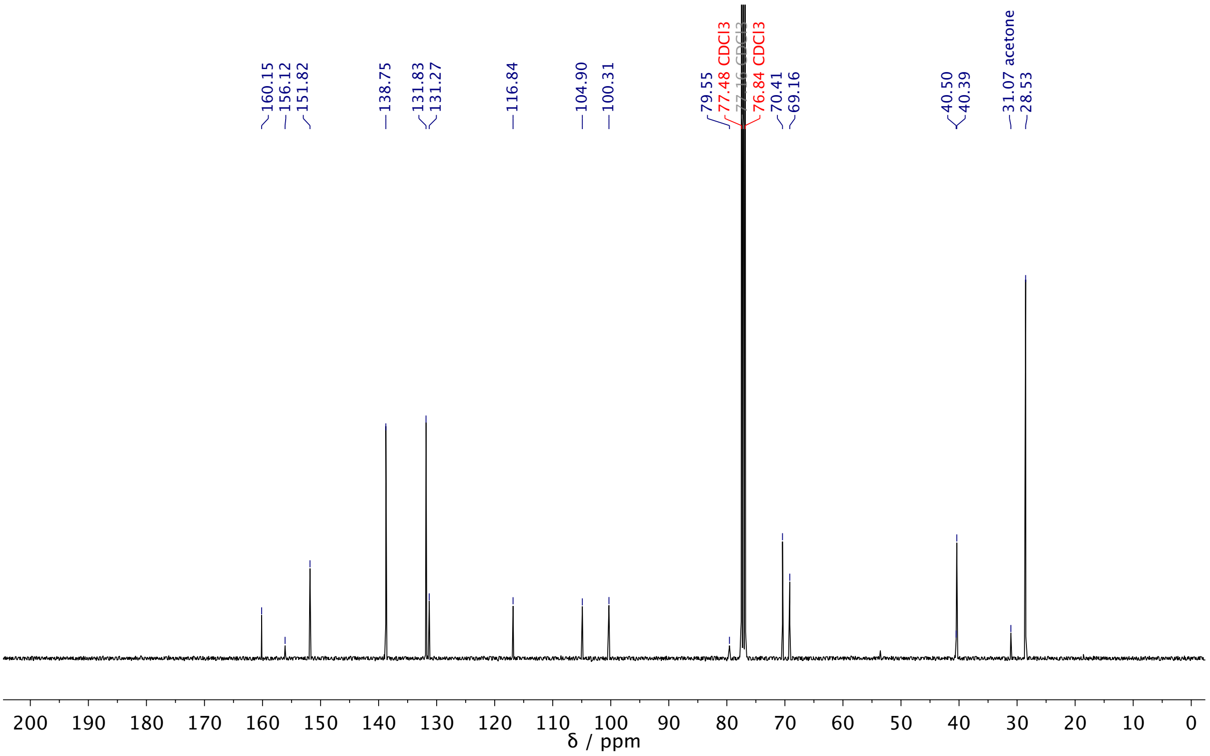

Figure S48. 101 MHz ^{13}C NMR spectrum of **56** in $CDCl_3$.

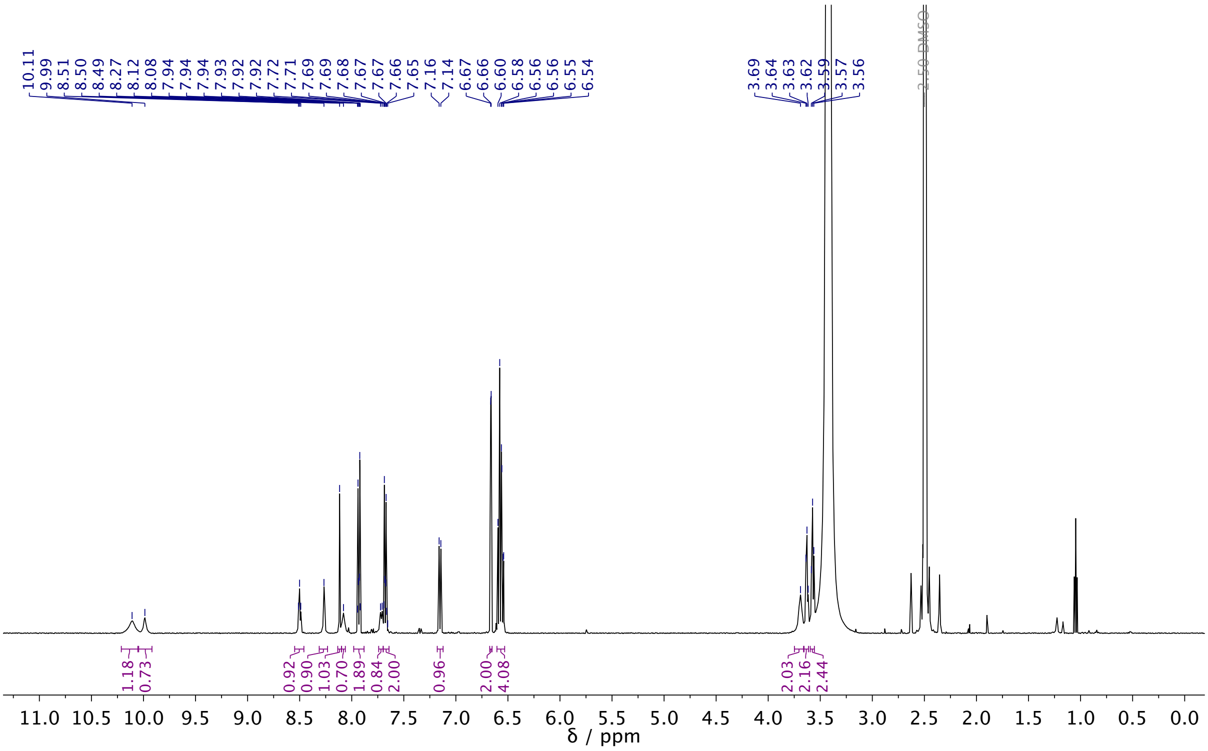

Figure S49. 500 MHz ^{1}H NMR spectrum of **16** in DMSO-d_6.

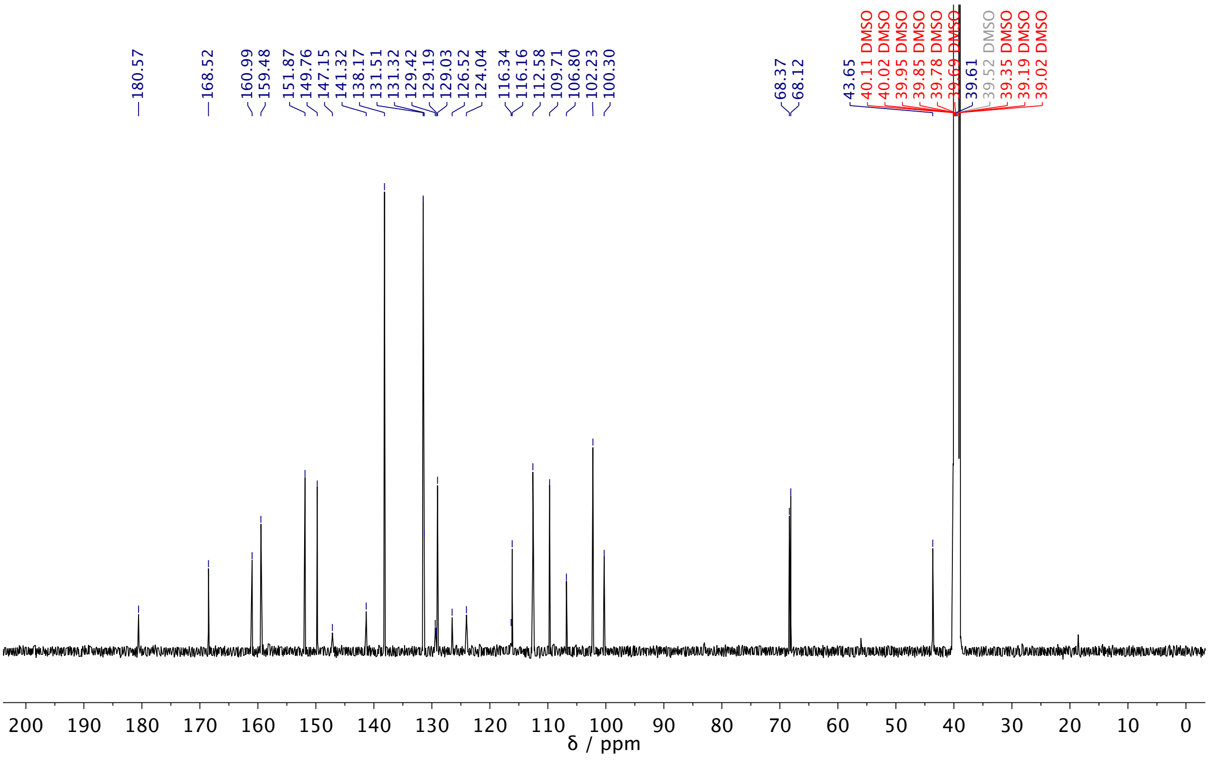

Figure S50. 126 MHz ^{13}C NMR spectrum of **16** in DMSO-d_6.
